# QuantifyPolarity, a new tool-kit for measuring planar polarized protein distributions and cell properties in developing tissues

**DOI:** 10.1242/dev.198952

**Published:** 2021-09-07

**Authors:** Su Ee Tan, Weijie Tan, Katherine H. Fisher, David Strutt

**Affiliations:** 1 Department of Biomedical Science, University of Sheffield, Western Bank, Sheffield S10 2TN, UK; 2 School of Computer Science, University of Nottingham, Jubilee Campus, Wollaton Road, Nottingham NG8 1BB, UK

**Keywords:** Planar polarity, PCP, Image analysis, Cell geometry

## Abstract

The coordination of cells or structures within the plane of a tissue is known as planar polarization. It is often governed by the asymmetric distribution of planar polarity proteins within cells. A number of quantitative methods have been developed to provide a readout of planar polarized protein distributions. However, previous planar polarity quantification methods can be affected by variation in cell geometry. Hence, we developed a novel planar polarity quantification method based on Principal Component Analysis (PCA) that is shape insensitive. Here, we compare this method with other state-of-the-art methods on simulated models and biological datasets. We found that the PCA method performs robustly in quantifying planar polarity independently of variation in cell geometry and other image conditions. We designed a user-friendly graphical user interface called QuantifyPolarity, equipped with three polarity methods for automated quantification of polarity. QuantifyPolarity also provides tools to quantify cell morphology and packing geometry, allowing the relationship of these characteristics to planar polarization to be investigated. This tool enables experimentalists with no prior computational expertise to perform high-throughput cell polarity and shape analysis automatically and efficiently.

## Abstract (precis)

**Summary:** We present a novel planar polarity quantification method based on Principal Component Analysis that performs robustly in quantifying planar polarity independently of variation in cell geometry and other image properties.

## INTRODUCTION

Planar polarity is crucial for coordinating the behavior of cells to generate highly organized structures at the tissue and organ level. It governs oriented cell divisions and rearrangements that specify tissue shape and generates global alignment of external structures such as *Drosophila* wing hairs, reptilian scales, mammalian hair and cilia, and stereocilia bundles in the ear (Devenport, 2016; Butler and Wallingford, 2017).

**Fig. 1.**
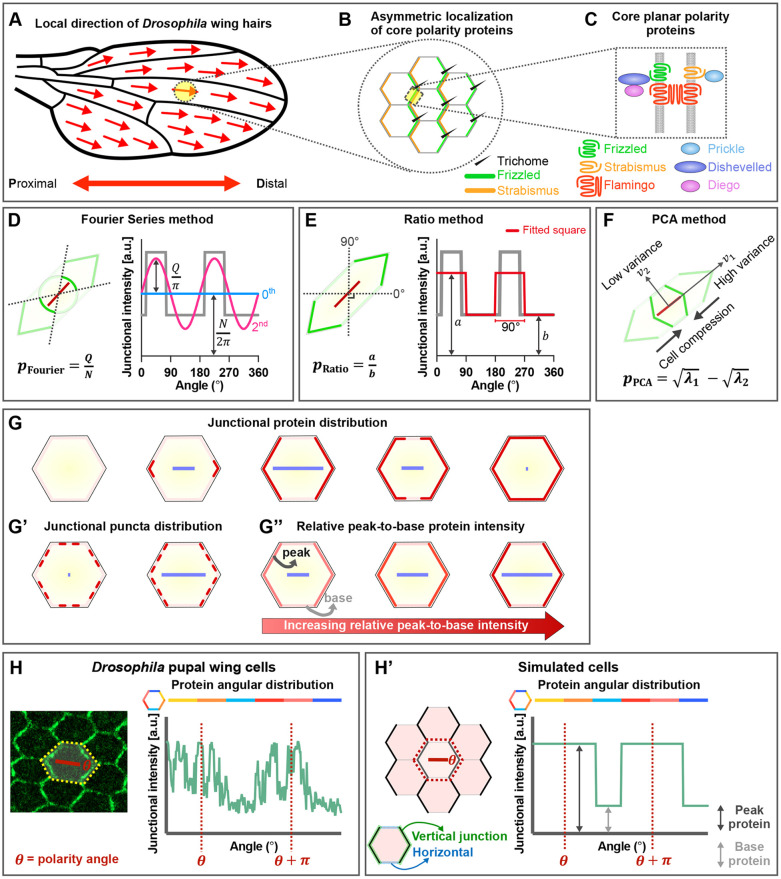
**Methods for quantitation of planar polarity.** (A) Cartoon of *Drosophila* wing blade. Red arrows indicate local hair orientation. (B) Core planar polarity proteins asymmetrically localize on opposite cell edges, with trichomes forming from the distal edge colocalizing with Frizzled (green) and opposite Strabismus (orange). Only the localizations of Frizzled and Strabismus are shown. (C) Asymmetric distribution of core planar polarity proteins at apical cell boundaries. (D-F) Schematics illustrating principles of planar polarity quantification of the (D) Fourier Series, (E) Ratio and (F) our novel PCA method, where *p* indicates polarity magnitude. (D) Given a polarized cell with the angular distribution of junctional protein intensities (gray line in graph), the Fourier Series method computes the 0^th^ (blue line) and 2^nd^ (pink line) orders of Fourier decomposition and determines polarity readout using Fourier coefficients. (E) The Ratio method fits a square wave (red line) onto the protein angular distribution of a cell (gray line) and computes the ratio between average intensities of opposite quadrants to determine the polarity magnitude. The angle that yields the largest ratio is the polarity angle. (F) The PCA method compresses cells into a regular shape and computes the angle (polarity angle) that produces the largest variance of normalized intensities. Polarity magnitude is determined from the eigenvalues (*λ*_1_, *λ*_2_) of both principal components (*v*_1_, *v*_2_). Red bars (D-F) indicate polarity readout. (G-G″) Cartoons showing notional polarity readouts (blue bars) computed for hypothetical cells. Polarity readout is determined based on junctional continuous or non-continuous (punctate) protein distribution and relative peak-to-base protein intensity. The blue bars represent the magnitude (where longer length indicates higher polarity) and angle (orientation of bar) of polarity for a given cell. (H,H′) Examples of protein angular distribution of (H) polarized pupal wing cell and (H′) simulated cells with vertical junctions (green edges) and horizontal junctions (blue edges). For all simulated cells, peak protein is higher intensity (255 a.u.), whereas base protein is lower intensity (40 a.u.), unless otherwise stated.

Tissue level cell behaviors are often regulated by polarized protein localizations. Examples are the asymmetric cellular localization of core planar polarity proteins such as Frizzled (Fz), which determines the placement of *Drosophila* wing hairs at distal cell junctions (Strutt, 2001) (Fig. 1A-C); the Fat-Dachsous system, in which intracellular asymmetry of Fat-Dachsous heterodimers (Ambegaonkar et al., 2012; Bosveld et al., 2012; Brittle et al., 2012; Merkel et al., 2014) results in the asymmetric distribution of the atypical myosin Dachs (Mao et al., 2006; Brittle et al., 2012); and planar polarization of proteins such as Myosin II, Rho kinase, E-Cadherin and Bazooka, which are required for cell rearrangements during *Drosophila* germ-band extension (Bertet et al., 2004; Zallen and Wieschaus, 2004; Blankenship et al., 2006; Tamada et al., 2012; Levayer and Lecuit, 2013; Kasza et al., 2014; Simões et al., 2014; Tetley et al., 2016).

Planar polarity in epithelia varies as cell morphology changes throughout development (Classen et al., 2005; Aigouy et al., 2010). Hence, addressing how cells with different shapes and sizes achieve planar polarization requires robust planar polarity quantification tools that are independent of cell geometry. A commonly used method is Fourier Series-based analysis, which computes the Fourier decomposition for the angular distribution of junctional protein intensities (from 0° to 360° as a periodic signal) and determines polarity magnitude and angle using Fourier coefficients (Fig. 1D) (Aigouy et al., 2010; Bardet et al., 2013; Merkel et al., 2014; Aw et al., 2016; Tetley et al., 2016; Banerjee et al., 2017). This method has been widely used; however, in its current implementation, it shows significant sensitivity to cell geometry (see Results).

A second approach calculates the ratio of fluorescence intensity of vertical to horizontal cell junctions (Farrell et al., 2017). Unlike methods using manual classification of cell junctions (Ambegaonkar et al., 2012; Brittle et al., 2012), junctions are automatically classified as horizontal or vertical, with a prior assumption that asymmetry is on this axis. However, this is poorly suited to proteins that are not polarizing along a specific axis or to cells with irregular geometry. A variant of this method fits a square wave onto the angular distribution of junctional protein intensities and computes the ratio of opposite quadrants to determine polarity magnitude and angle on a cell-by-cell basis (Fig. 1E) (Strutt et al., 2016). This approach is applicable to polarization on any cell axis, but is still challenged by irregular-shaped cells.

Here, we present an unbiased and automated method to quantify asymmetric distributions of proteins on cell boundaries based on Principal Component Analysis (PCA). This method compresses cells into regular shapes and computes the angle (polarity angle) that produces the largest variance of normalized intensities. Polarity magnitude is then determined from the eigenvalues (*λ*_1_, *λ*_2_) of both principal components (*v*_1_, *v*_2_), independently of cell geometry (Fig. 1F). To evaluate this approach, we compare it with other published methods (Fourier Series and Ratio) on simulated models and experimental data. Additionally, we provide a user-friendly QuantifyPolarity Graphical User Interface (GUI) as a general tool for the study of epithelial tissue dynamics, including quantification of planar polarization and cell characteristics such as morphology and topology, allowing correlations between different cell properties to be explored.

## RESULTS

### Validation of planar polarity quantification methods

There are two readouts of cell polarity: strength of polarization (‘polarity magnitude’) and axis of polarization (‘polarity angle’). Polarity magnitude is dependent on how proteins are distributed on cell junctions and the relative peak-to-base protein intensity (Fig. 1G-G″); a good polarity method should robustly detect different degrees of polarization magnitude based on variations in these parameters. If continuous or non-continuous (punctate) proteins are homogeneously distributed on cell junctions, the cell is unpolarized. If proteins are asymmetrically segregated to opposite junctions (‘bipolarity’), then the cell is polarized and exhibits higher polarity magnitude (Fig. 1G,G′). Cells with higher relative peak-to-base intensity exhibit higher polarity magnitude (Fig. 1G″). Polarity angle is defined as the axis that provides the maximum asymmetry. Although, in principle, unipolarity could also be measured (asymmetric localization of a protein to one side of a cell), within an epithelium the tight apposition of neighboring cell junctions generally makes it impossible to distinguish a unipolarized distribution from a bipolarized distribution. In this work, we consider methods designed to measure bipolarity.

As cells exhibit different shapes and sizes throughout development, a polarity quantification method unaffected by such changes is highly desirable. The definition of cell shape independence is adopted from ratio methods of quantifying polarity, where polarity magnitude is computed as the ratio of average protein on opposite junctions, rendering this method independent of junction length and hence cell elongation. If instead total protein on a cell junction is considered, even with a homogeneous protein distribution, more elongated cells will appear more polarized than less elongated cells simply because longer junctions have higher total protein. Similarly, larger cells should not appear more polarized than smaller ones and polarity angle should be oriented on the axis of maximum asymmetry, unaffected by cell geometry. Here, we explore the robustness of different polarity methods in detecting polarization when challenged with varying cell sizes, shapes, eccentricities, protein distribution, relative peak-to-base intensity and image conditions. Specifically, we validate the PCA method and compare it with the Ratio and Fourier Series methods, on simulated cells and biological datasets.

### Validation on simulated cells

To assess performance of different polarity quantitation methods in the face of varying cell geometry, we simulated cells with varying size, shape regularity, eccentricity and the amount of proteins on cell junctions, and also varying image conditions, such as brightness and signal-to-noise ratio.

Before *Drosophila* pupal wing hair formation, Fz protein becomes concentrated to distal cell junctions and is unipolarized (Strutt, 2001). Owing to limited resolution of confocal microscopy, Fz localization on one side of a junction is inseparable from that on the other, so Fz appears to be both distal and proximal, and hence bipolarized (Fig. 1H). The angular distribution profile is the protein intensities on cell junctions, spanning 0°-360° with respect to the cell centroid. From the angular distribution profile, one expects two peaks of Fz protein intensity at *θ* and *θ*+*π*, corresponding to the polarity angle (Fig. 1H). We simulated hexagonal cells with junctional proteins on both horizontal and vertical junctions (Fig. 1H′). Simulated cells have two intensity levels, whereby proteins on vertical junctions exhibit higher intensity (‘peak protein’), while proteins on horizontal junctions exhibit lower intensity (‘base protein’), unless otherwise stated. We then quantified polarity magnitude and angle obtained from different methods on simulated cells of different geometries and image conditions. Polarity magnitudes obtained from different methods are normalized against their maximum magnitudes to allow direct comparison unless otherwise stated.

When we gradually increased the absolute amount of peak and base protein intensities (while maintaining relative peak-to-base intensity) in simulated cells, neither polarity magnitude nor angle was affected for all methods (Fig. S1A-A″). Hence, all the methods are independent of varying image brightness/intensity. Thereafter, peak and base protein intensities were set to 255 and 40 arbitrary units (a.u.), respectively, for all two-intensity level simulations.

**Fig. 2.**
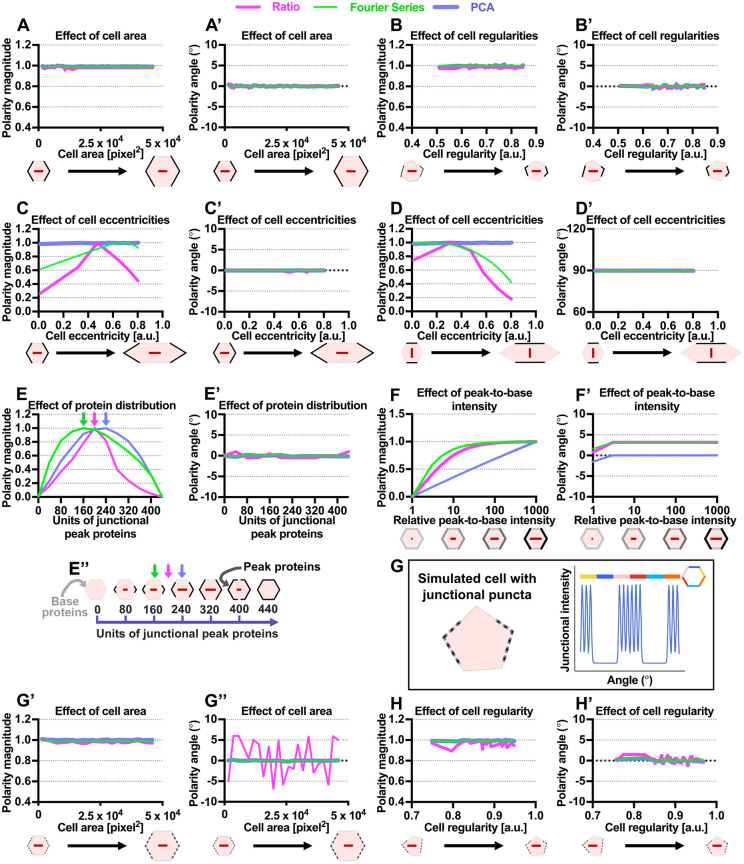
**Comparison between three methods of planar polarity quantification using simulated cells.** (A-D′) Quantified polarity magnitudes and polarity angles of cells with (A,A′) varying apical area from 1500 to 46,000 pixels^2^ (∼10-300 μm^2^), (B,B′) varying shape regularity from 0.5 to 0.85 a.u., (C,C′) varying cell eccentricity (elongation) from 0 to 0.8 a.u. with peak protein on vertical junctions and (D,D′) varying cell eccentricity from 0 to 0.8 a.u. with peak protein on horizontal junctions. (E-E″) Quantified polarity magnitudes and polarity angles of cells with varying junctional protein distribution. Given a cell with total perimeter of 440 pixels, units of junctional peak proteins increase gradually, starting from both poles of vertical junctions. (E″) Arrows indicate units of junctional peak proteins, which gives maximum polarity magnitude for each method (magenta, Ratio; green, Fourier Series; blue, PCA). (F,F′) Quantified polarity magnitudes and polarity angles of cells with varying relative peak-to-base protein intensities. (G) Simulated cell with non-continuous junctional puncta protein distribution. Each punctum exhibits a junctional intensity profile of a Gaussian function (intensity value ranges from 40 to 255 a.u., puncta spacing of 15° and Gaussian sigma of 4.47). (G′-H′) Quantified polarity magnitudes and polarity angles of cells with punctate protein when (G′,G″) apical area varies and (H,H′) shape regularity varies. All polarity magnitudes obtained using different methods are normalized to allow comparison. All polarity angles (in degrees) range between −90° and +90°, with 0° corresponding to the *x*-axis of the image.

In *Drosophila* wild-type pupal wing cells, average apical area varies between 2000 and 2800 pixels^2^ (∼12-18 μm^2^) using our typical imaging settings, between 24 and 36 h after puparium formation (hAPF) (Fig. S1B). Removing *dumpy* activity results in a shorter wing blade and a reduced cell area (Etournay et al., 2015; Ray et al., 2015) of ∼1700 pixels^2^ (∼10 μm^2^) at 30 hAPF (Fig. S1E,E′). Conversely, *ultrahair* and *cdc2* mutations produce apical cell areas ∼4-16 times larger than normal (Adler et al., 2000). Hence, we simulated cells with apical areas ranging from ∼1500 to 46,000 pixels^2^ (∼10-300 μm^2^), while maintaining the amount of protein on vertical cell junctions (peak protein spanning ±60° with respect to the cell centroid). Despite having different areas, these simulated cells had equivalent protein angular distribution profiles, and accordingly show similar polarity magnitude and angle with all three polarity methods (Fig. 2A,A′).

During *Drosophila* pupal wing development, cell shape changes from irregular to highly regular in geometry (∼0.65-0.85 a.u., 0 being highly irregular and 1 being perfectly regular) (Classen et al., 2005) (Fig. S1C). Moreover, loss of Rap1 results in highly aberrant cell shape (Knox and Brown, 2002) when compared with wild-type cells (Fig. S1E,E″). Hence, we simulated cells with varying shape regularity from 0.5 to 0.85, while maintaining the amount of proteins on the vertical cell junctions (peak proteins spanning ±30°). We found that the polarity magnitudes and angles obtained from all methods are unaffected by variation in cell regularities (Fig. 2B,B′). Hence, all the polarity methods are suitable for quantifying planar polarity in cells with varying cell sizes and shapes.

Furthermore, Aigouy et al. (2010) showed that from 24 to 36 hAPF, *Drosophila* pupal wing cell elongation (eccentricity) gradually decreases from 0.6 to 0.2 a.u. (0 being circular and 1 being highly elongated) (Fig. S1D). Thus, ideally a method for quantifying polarity should not be affected by cell eccentricity. We simulated cells with eccentricity varying from 0 to 0.8, while maintaining the amount of peak protein on vertical junctions (Fig. 2C,C′). We also simulated cells while maintaining amount of peak protein on horizontal junctions, as seen in some biological contexts (Aw et al., 2016; Devenport, 2016) (Fig. 2D,D′). In both cases, the polarity magnitude computed using the PCA method is independent of cell eccentricity (Fig. 2C,D). However, both the Ratio and Fourier Series methods are sensitive to varying cell eccentricity. For example, with peak protein on vertical junctions, the Fourier Series method gave maximum polarity magnitude for cells with an eccentricity of 0.7, while polarity magnitude using the Ratio method is significantly reduced for cells with eccentricities above and below 0.5. Nevertheless, all the methods give a constant polarity angle readout at 0° (Fig. 2C′,D′).

We note that an elongated cell results in a different protein angular distribution when compared with a regular cell. As the polarity readout from the Ratio and Fourier Series methods is determined using the protein angular distribution, this results in polarity magnitude varying with cell eccentricity. Our PCA method uses a cell compression operation to compress an elongated cell into a regular cell, hence preserving the protein angular distribution, making this method insensitive to varying eccentricity.

During pupal wing development, core proteins, such as Fz, become increasingly polarized before gradually depolarizing after the emergence of wing hairs (Usui et al., 1999; Classen et al., 2005; Aigouy et al., 2010; Merkel et al., 2014). Hence, we evaluated the performance of different methods in detecting different degrees of polarization strength due to varying junctional protein distribution (Fig. 1G). We simulated a regular hexagon with a perimeter length of 440 units initially all set to base protein intensity. We then varied the protein distribution by gradually increasing peak protein distribution on vertical junctions starting at the poles of the cell and moving onto the horizontal junctions (Fig. 2E″).

A simulated cell with an homogenous distribution of base protein exhibited no polarization (Fig. 2E,E″). Gradual increments of peak protein distribution on the poles of the vertical junctions resulted in increasing polarity magnitude. However, the maximum polarity magnitude obtained from all methods varied. Maximum polarity is achieved when the amount of the cell perimeter comprising peak protein is ∼200 units for Ratio, ∼160 for Fourier Series and ∼240 for PCA (arrows in Fig. 2E,E″). Polarity magnitudes then decrease steadily, reaching zero when peak protein becomes homogenously distributed (Fig. 2E,E″). For polarity angle, all methods remain consistently oriented at 0° (Fig. 2E′). Thus, we found that there is a distinct polarization strength profile for each method. This allows users to choose a method according to their requirements. For example, prior to *Drosophila* pupal wing hair formation, Fz becomes highly polarized to distal junctions (Strutt, 2001), represented by a simulated cell with 320 units of peak protein on vertical junctions (Fig. 2E″). Hence, the PCA method, which gives maximum polarity magnitude at ∼240 units of peak protein, may be best suited for measuring Fz polarity. Additionally, the PCA method also exhibits a more symmetrical polarization strength profile when compared with the other methods, whereby cells with 40 and 400 units of peak protein are equally (weakly) polarized (Fig. 2E).

We further extended these simulations by varying protein distribution while simultaneously elongating cells (Fig. S1F,G′). The PCA method successfully detected changes in protein distribution that were independent of cell eccentricity. On the contrary, polarity magnitude obtained from both the Ratio and Fourier Series methods remained constant due to the sensitivity of these methods to cell eccentricity. For polarity angle, all methods remain consistently oriented at 0° or 90° (Fig. S1F′,G′).

An important criterion for a robust polarity method is the ability to detect different degrees of polarization strength given variation in relative peak-to-base protein intensity (Fig. 1G″). We simulated cells with increasing peak protein intensity on vertical junctions while maintaining a constant base intensity (40 a.u.) on horizontal junctions (Fig. 2F). For simulated cells with relative peak-to-base ratio intensity of 1 (equivalent peak and base intensities), the cell is non-polarized while increasing peak protein on the vertical junctions results in increasing polarity magnitude. Polarity magnitude is plotted against the relative peak-to-base intensity in log scale, in which a straight line indicates that when relative peak-to-base intensity increases by a fixed percentage, the polarity magnitude increases by a corresponding fixed amount. The PCA method exhibits this characteristic for all relative peak-to-base values (Fig. 2F). Unlike the PCA method, both the Ratio and Fourier Series methods exhibit such characteristic only at low relative peak-to-base values before eventually plateauing (Fig. 2F). Thus, the PCA method is more reliable in detecting polarization strength for all relative peak-to-base intensity values.

It is also important to have a method that performs well on images with varying signal-to-noise ratios (SNR). We assessed the performance of each method on simulated cells with added random normally distributed noise (Fig. S2A). All methods displayed some sensitivity to noise but, even at high noise (low SNR), the maximum relative error in polarity magnitude is no more than 13% and the angle reported by the Ratio method shows fluctuations of ±13° (Fig. S2A′,A″).

Rather than just two intensity levels, biological cells exhibit multiple levels of junctional protein intensities, following a non-continuous punctate protein distribution (Figs 1H and 2G). Hence, we questioned whether a punctate protein distribution affects the performance of each polarity method on simulated cells with varying area, regularity and puncta distribution. For the Fourier Series and PCA methods, there were no significant differences in polarity readouts with a punctate protein distribution (Fig. 2G′-H′, Fig. S2B,B′). However, the Ratio method fluctuates with maximum relative error ±10.6% for polarity magnitude and ±40° error for polarity angle for all simulations. These fluctuations can be attributed to the discretization of the Gaussian intensity profile of puncta in simulated cells. Additionally, results from our simulations suggest that all methods produce inconsistent polarity readouts for tricellular junction protein localization on irregular-shaped cells (Fig. S2C-C″). Hence, none of the methods are suited to quantify tricellular junction localization.

In summary, we have tested the performance of each quantification method on a range of different cell geometries, continuous or punctate proteins distributions, relative peak-to-base intensity and image conditions. Overall, the PCA method most successfully quantifies polarity in an unbiased manner, independently of different cell size, shape and eccentricity. Besides that, each polarity method has its own unique polarity strength profile, which could be advantageous for the analysis of different types of polarized cells. These simulation results will hopefully serve as a reference for users to choose the most appropriate method best suited to their system (see Table 1).

**Table 1.**
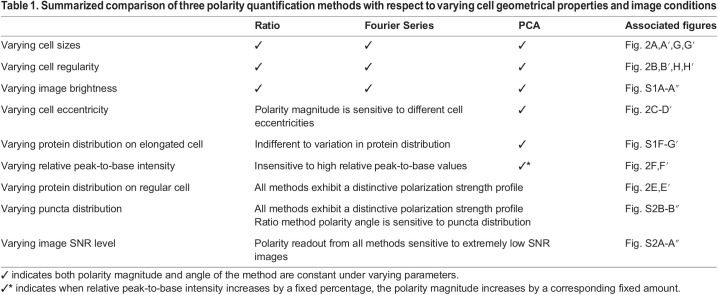
Summarized comparison of three polarity quantification methods with respect to varying cell geometrical properties and image conditions

### Validation of different polarity quantification methods on biological datasets

**Fig. 3.**
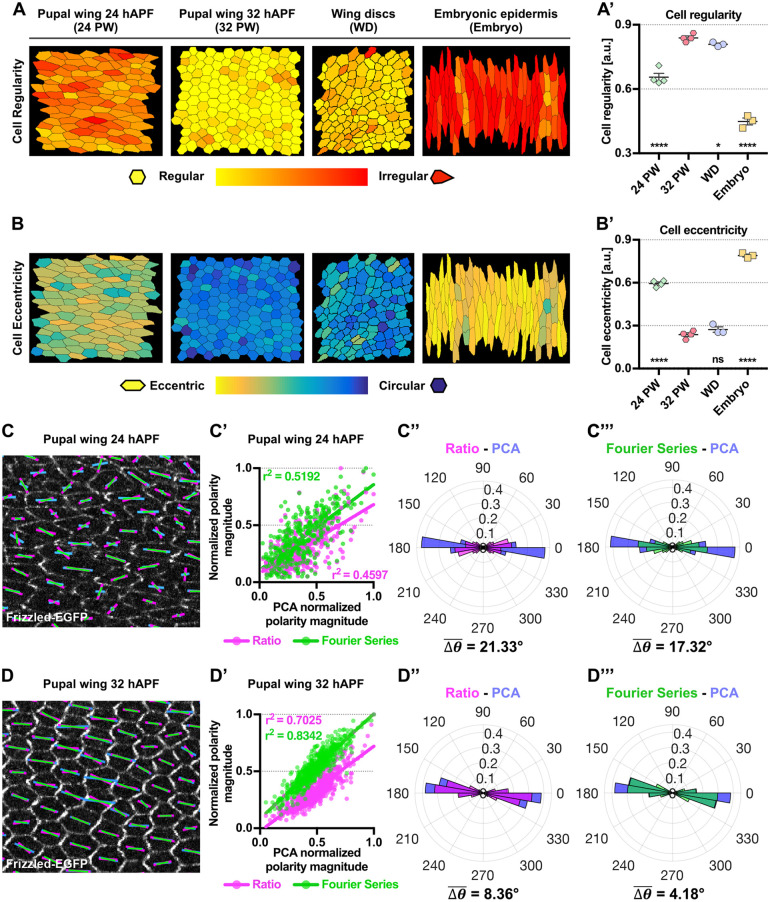
**Comparison of methods for planar polarity quantification on *Drosophila* pupal wings at different developmental timepoints.** (A,B) Processed images of different planar polarized epithelial tissues: 24 and 32 hAPF *Drosophila* pupal wing, third instar wing discs and embryonic epidermis, each exhibiting varying cell regularity and eccentricity when compared with regularly packed hexagonal cells. Cells are color-coded according to: (A) the regularity of the shape, with yellow being perfectly regular and red representing highly irregular; and (B) the eccentricity of the shape, with yellow representing highly eccentric and blue being circular. (A′,B′) Quantified average cell regularity and cell eccentricity of different epithelial tissues (*n*=3 to 4 per tissue type). Dot indicates individual tissue type, error bars show the s.e.m. One-way ANOVA unpaired test, comparing each epithelial tissue to 32 hAPF pupal wing. ****P≤0.0001, *P≤0.01; ns, not significantly different. (C,D) Quantified cell-scale polarity pattern of otherwise wild-type wings expressing Fz-EGFP at (C) 24 hAPF and (D) 32 hAPF using the three different methods. The magenta (Ratio), green (Fourier Series) and blue (PCA) bars represent the magnitude (length of bar) and angle (orientation of bar) of planar polarization for a given cell. (C′,D′) Plots of normalized polarity magnitudes at (C′) 24 hAPF and (D′) 32 hAPF obtained from Ratio (magenta dots) and Fourier Series (green dots) versus PCA methods, with best fit lines shown in magenta and green, respectively. Coefficients of determination (r^2^) are indicated. (C″,C‴,D″,D‴) Circular weighted histogram plots displaying the orientation of Fz-EGFP polarity obtained from (C″,D″) Ratio (magenta) and PCA (blue), and (C‴,D‴) Fourier Series (green) and PCA methods at 24 hAPF (C″,C‴) and 32 hAPF (D″,D‴) with mean angle difference 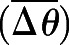 indicated (*n*=4 wings per timepoint, 600 cells analyzed). All polarity magnitudes obtained using different methods are normalized to allow comparison. All polarity angles range between 0° and 360°, with 0° corresponding to the *x*-axis of the image.

Although the PCA method performs effectively in quantifying polarity on simulated cells, this might not necessarily reflect its performance on biological datasets. We therefore compared results obtained from the PCA method with the Fourier Series and Ratio methods, on images of different planar polarized epithelial tissues, specifically the *Drosophila* pupal wing, third instar wing discs and the embryonic epidermis, which each exhibit distinct cell geometries (Fig. 3A-B′).

### *Drosophila* pupal wing analysis

The simulation results allow us to better understand the behavior of each polarity method. However, none of the simulated cases represent the real biological scenario, in which both cell morphology and protein distribution change in concert. This is particularly striking during *Drosophila* pupal wing morphogenesis, where polarization of planar polarity proteins increases concomitantly with changes in cell size, shape regularity and eccentricity (Fig. S1B-D) (Classen et al., 2005; Aigouy et al., 2010). Hence, pupal wing morphogenesis provides a dynamic system for comparing the performance of different methods on biological data. We therefore analyzed the correlation between the polarity magnitude obtained from the different methods, of EGFP-tagged Fz (a core planar polarity protein) in otherwise wild-type wings at two developmental timepoints (Fig. 3C,D). At 24 hAPF, when cells are more eccentric, there is only a moderate correlation between the PCA method and the results obtained using the Ratio (coefficient of determination, r^2^=0.4597) and Fourier Series (r^2^=0.5192) methods (Fig. 3C′). This is likely to be due to polarity magnitudes computed from the Fourier Series and Ratio methods being more sensitive to cell eccentricity, as evident from the simulation results (Fig. 2C). However, the correlation between the Ratio and Fourier Series to PCA method improved (with r^2^=0.7025 and 0.8342, respectively) as cells become less eccentric by 32 hAPF (Fig. 3D′).

We computed the mean angle difference, 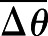, to compare polarity angles obtained from the PCA against the Ratio and Fourier Series methods (see Materials and Methods). When compared with 32 hAPF, polarity angles obtained from both the Ratio and Fourier Series methods at 24 hAPF are less in agreement with the PCA method (with 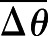 of 21.33° and 17.32°, respectively) (Fig. 3C″,C‴). However, by 32 hAPF, polarity angles computed from both methods agree better (with 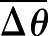 of 8.36° and 4.18°, respectively) (Fig. 3D″,D‴).

As a comparison with the polarized distribution of Fz-EGFP, we also quantified the polarization of E-Cadherin::GFP at 32 hAPF, which displays weak anteroposterior asymmetry in the pupal wing (Warrington et al., 2013) (Fig. S3A). Quantification of E-Cadherin::GFP distribution with all the polarity methods produces low but non-zero polarity magnitude (Fig. S3A′). Furthermore, E-Cadherin::GFP distribution also exhibits dispersed angles (Fig. S3B-C″). To capture the local coordination of polarity, we quantified and compared the coarse-grain polarity (vector average polarity over local groups of cells) of E-Cadherin::GFP and Fz-EGFP at 32 hAPF (Fig. S3D,D′) (see Materials and Methods). Indeed, coarse-grain polarization of E-Cadherin::GFP is significantly smaller than Fz-EGFP, with E-Cadherin::GFP showing at best weak local polarity coordination (Fig. S3D,D′).

Even for unpolarized (but non-homogenous, e.g. punctate) distributions of proteins, each method nevertheless will report a readout of polarity magnitude and angle. We therefore recommend the use of weighted histograms that use polarity magnitude as a weight for the angles (see ‘Circular weighted histogram’ in the Materials and Methods) (Aw et al., 2016), rather than simply plotting unweighted polarity angles, which results in more dispersed polarity angles (compare Fig. S3B-B″ and Fig. S3C-C″). Moreover, quantifying a poorly polarized protein such as E-Cadherin provides a baseline polarity readout, which can be an important control for comparison with well-polarized proteins.

### *Drosophila* wing discs

**Fig. 4.**
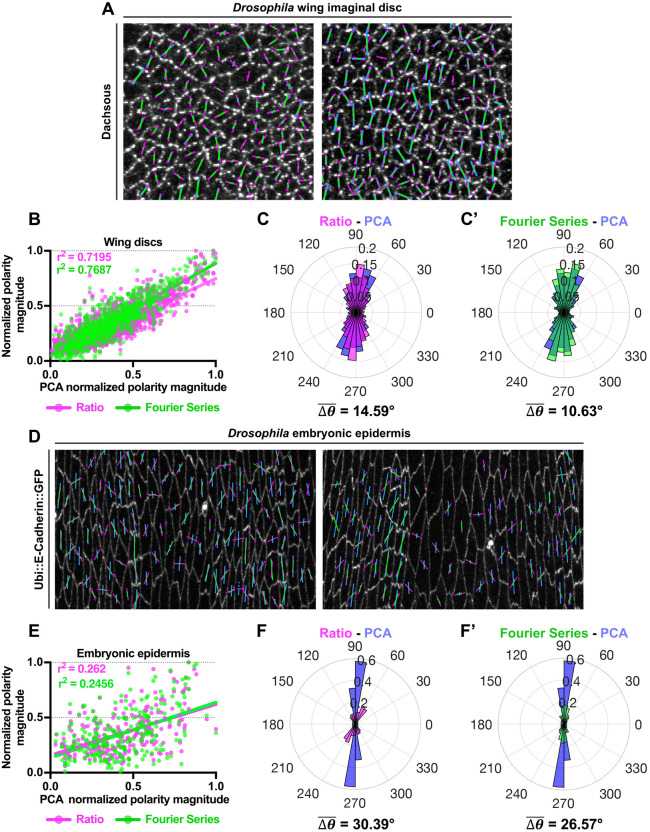
**Validation of different methods for quantification of planar polarity on *Drosophila* wing imaginal discs and embryonic epidermal cells.** (A) Quantified cell-scale polarity pattern of two examples of wing discs immunolabeled for Dachsous in third-instar larval imaginal discs. The magenta (Ratio), green (Fourier Series) and blue (PCA) bars represent the magnitude and angle of planar polarization for a given cell. (B) Plot of normalized polarity magnitudes obtained from Ratio (magenta dots) and Fourier Series (green dots) versus PCA methods with best fit lines shown in magenta and green, respectively. Coefficients of determination, r^2^, are indicated. (C,C′) Circular weighted histogram plots display the orientation of Dachsous polarity obtained from (C) Ratio (magenta) and PCA (blue), and (C′) Fourier Series (green) and PCA methods with its mean angle difference 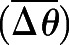 (*n*=3 wing discs, 900 cells analyzed). (D) Two examples of quantified cell-scale polarity pattern of Ubi::E-Cadherin-GFP-expressing epidermal embryonic cells at stage 15. (E) Plot of normalized polarity magnitudes of embryonic epidermal obtained from Ratio (magenta dots) and Fourier Series (green dots) versus PCA methods with best fit lines shown in magenta and green, respectively. Coefficients of determination, r^2^, are indicated. (F,F′) Circular weighted histogram plots display the orientation of Ubi::E-Cadherin::GFP polarity obtained from (C) Ratio (magenta) and PCA (blue) and (C′) Fourier Series (green) and PCA with its mean angle difference 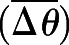 (*n*=3 embryos, 250 cells analyzed). All polarity magnitudes obtained using different methods are normalized to allow comparison. All polarity angles range between 0° and 360°, with 0° corresponding to the *x*-axis of the image.

Next, we quantified the asymmetric localization of the Dachsous planar polarity protein in third-instar larval wing imaginal discs using all three polarity methods (Fig. 4A). For polarity magnitude, both the Ratio and Fourier Series methods correlate well with the PCA method (r^2^=0.7195 and 0.7687 for Ratio and Fourier Series, respectively) (Fig. 4B). Moreover, the polarity angles obtained from the Fourier Series and PCA methods are slightly more in agreement when compared with the Ratio and PCA methods (with 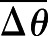 of 10.63° and 14.59°, respectively) (Fig. 4C,C′). In fact, there is only a slight difference between the geometry of third-instar wing pouch cells from that of 32 hAPF pupal wing cells (Fig. 3A′,B′), and so similarly there is good correlation between all three methods.

### *Drosophila* embryonic epidermis

Finally, we quantified Ubi::E-Cadherin-GFP asymmetry in images of lateral epidermal cells in *Drosophila* embryos at stage 15, where the embryonic epidermal cells exhibit an elongated rectangular shape (Fig. 4D). The embryonic epidermal cells are much more irregular and eccentric in geometry when compared with both pupal wing and wing disc cells (Fig. 3A′,B′). Based on published results from Bulgakova et al. (2013), E-Cadherin is asymmetrically localized to the shorter cell junctions and, therefore, should exhibit an approximately ±90° angle of polarization (along the *y*-axis in the image). Notably, polarity angles of embryonic epidermal cells computed using the PCA method are well-aligned along ±90° (with angle variance of 0.038±0.013), in agreement with the published results (Bulgakova et al., 2013) (Fig. 4D). On the contrary, polarity angles obtained from both the Ratio and Fourier Series methods are more dispersed from −90° to +90° (with angle variances of 0.52±0.04 and 0.38±0.03, respectively), disagreeing with the previous analysis (Bulgakova et al., 2013) (Fig. 4F,F′). There are higher differences in polarity angle between both methods and the PCA method for epidermal embryonic cells when compared with both pupal wing and wing discs cells (with 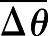 of 30.39° and 26.57°, respectively). One explanation for this might be the influence of junctional proteins from abutting cells, particularly where they form tricellular junctions with the cell of interest. When considering angular protein distribution of an elongated cell, these tricellular junctional proteins on longer junctions are closer to the cell centroid and exhibit higher ‘weighting’ when compared with proteins that are on the shorter junctions of the cell. As polarity readout from both the Ratio and Fourier Series methods is determined from the angular protein distribution, this results in incorrect quantification of polarity angles using these methods.

In terms of polarity magnitude, both the Ratio and Fourier Series methods are also poorly correlated with the PCA method (r^2^=0.262 and 0.2456 for Ratio and Fourier Series, respectively) (Fig. 4E). This is likely due to both the excess weighting of protein at tricellular junctions on the longer cell junctions exhibited by the Fourier Series and Ratio methods, and also more generally due to the presence of elongated cells, as polarity magnitudes computed will be further affected by variation in cell eccentricities, as evident from the simulation results (Fig. 2C).

### QuantifyPolarity GUI: an automated tool for quantification of planar polarization and cell shape

**Fig. 5.**
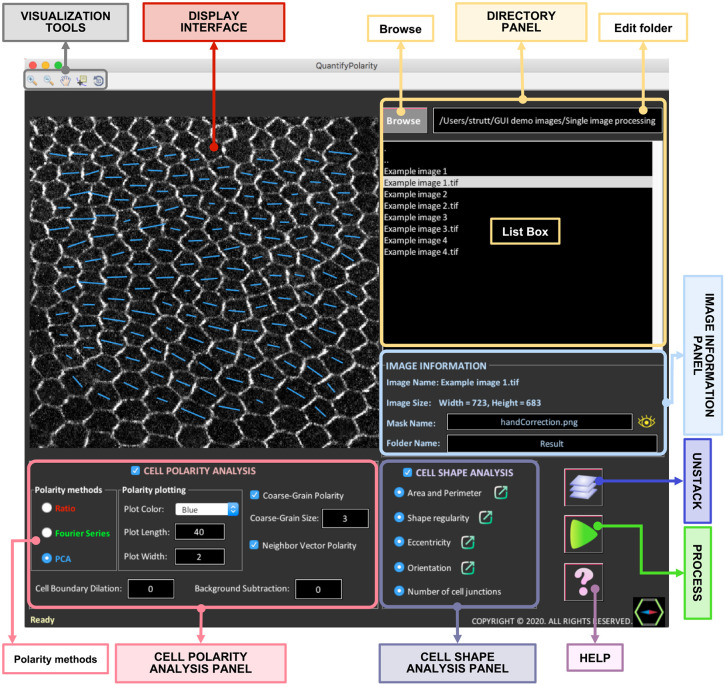
**QuantifyPolarity graphical user interface for quantification of planar cell polarity and cell shape.** The QuantifyPolarity GUI integrates all three polarity methods (Ratio, Fourier Series and PCA) for planar polarization quantification. Additional functionality of QuantifyPolarity includes 2D quantitative analysis of cell morphological properties.

The QuantifyPolarity GUI (Fig. 5) was developed to provide fast and reliable analysis of 2D planar polarity in multicellular tissues by incorporating all the three quantification methods described here – Ratio, Fourier Series and PCA. These methods are applicable to any 2D asymmetrical distribution of proteins on cell junctions. All three methods are useful for quantifying planar polarization on cells with regular geometry; however, for more complex geometries, the PCA method overall performs better (Table 1).

The cell-by-cell polarity readout obtained from the Ratio, Fourier Series and PCA methods reveals the polarization strength and alignment of each individual cell (Fig. S4B′, part i). Averaging this value (‘Average Polarity Magnitude’) gives a measure of polarization strength of all cells within the image, without taking into consideration the coordination of polarity between cells. Conversely, vector polarity measurement is defined to capture both the polarity magnitude and coordination of all cells within an image field (referred to as ‘Vector Average Polarity’), between groups of cells within a defined area (‘Coarse-Grain Vector Polarity’) and with its immediate neighbors (‘Neighbor Vector Polarity’) (see Materials and Methods) (Fig. S4B′, parts ii, iii). The ‘Angle Variance’ measures the variance in polarity alignment of all cells within the image. See Fig. S6 for a summarized explanation of the differences between each polarity measurement on various examples of polarized tissues.

In addition to polarity quantification, QuantifyPolarity also includes quantitative analysis of several cell morphological properties (e.g. size, shape regularity, eccentricity and orientation) and topology (number of neighbors), which are useful for the study of morphogenesis (Fig. S4B″). QuantifyPolarity also generates (customizable) color-coded images corresponding to the quantitative measurements, allowing users to directly visualize and inspect the results of the quantification. For example, each cell is color-coded with a gradient color-map according to their apical area, shape regularity, eccentricity, orientation and number of cell junctions, allowing visualization of the temporal and spatial evolution of cell geometries (Fig. S5). All results are automatically generated by QuantifyPolarity for further analysis.

Additionally, we added a feature that allows the user to perform measurements within multiple different regions on the same image. Equipped with the functionality of batch processing, users can automate and accelerate the analysis of multiple images within the same folder, which is often a time-consuming process. Finally, QuantifyPolarity can operate as a standalone GUI on a range of platforms such as Mac and Windows without requiring additional software.

### Analysis of temporal evolution of planar polarity and cell morphology in QuantifyPolarity

**Fig. 6.**
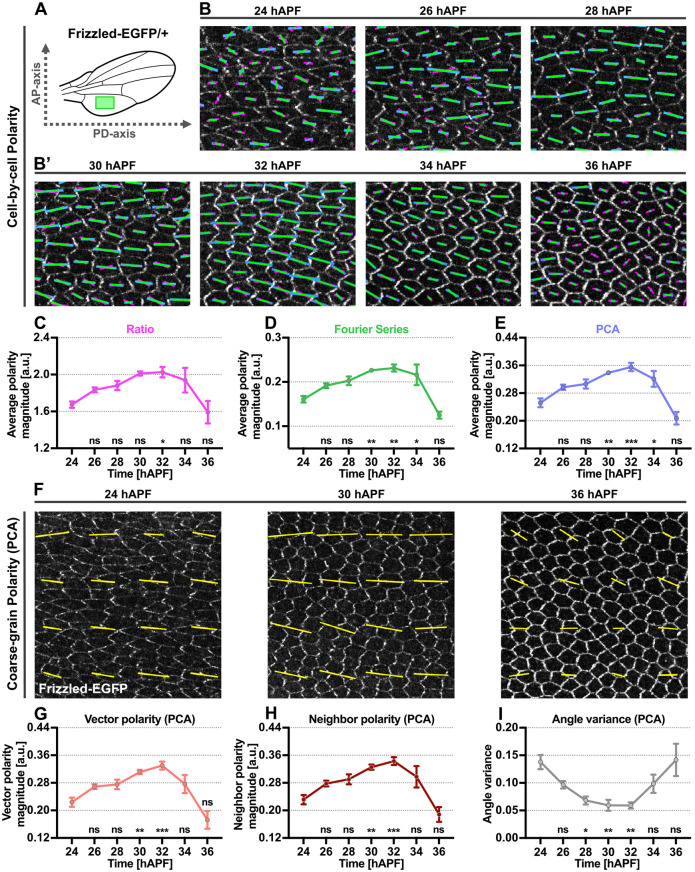
**Application of different methods for planar polarity quantification on *Drosophila* pupal wings at varying developmental timepoints.** (A) Illustration of analyzed proximal-posterior region below vein 5 of the wild-type pupal wing blade (green box). (B,B′) Quantified cell-scale polarity pattern of otherwise wild-type wings expressing Fz-EGFP from 24 to 36 hAPF. The magenta (Ratio), green (Fourier Series) and blue (PCA) bars represent the magnitude and angle of planar polarization for a given cell. (C-E) Plots of average polarity magnitudes (a.u.) obtained from the (C) Ratio, (D) Fourier Series and (E) PCA methods for Fz-EGFP over time. (F) Coarse-grain polarity pattern of otherwise wild-type wings expressing Fz-EGFP at 24, 30 and 36 hAPF. The yellow bars represent the magnitude (length of bar) and angle (orientation of bar) of planar polarization for a group of cells obtained from the PCA method. (G-I) Plots of (G) vector average polarity magnitude, (H) neighbor average polarity magnitude and (I) angle variance obtained from the PCA method for Fz-EGFP wings at indicated timepoints. *n*=5 wings per timepoint. Dot indicates mean, error bars show the s.e.m. One-way ANOVA unpaired test, comparing each timepoint to 24 hAPF pupal wing. ***P≤0.0005, **P≤0.002 and *P≤0.01; ns, not significantly different.

As a demonstration of the functionalities of QuantifyPolarity, we investigated the temporal evolution of cell polarity and cell morphological properties during *Drosophila* pupal wing development. We quantified polarization magnitude of Fz-EGFP in the proximal-posterior region of otherwise wild-type wings (Fig. 6A) from 24 to 36 hAPF using the three polarity methods (Fig. 6B,B′).

All three methods displayed a similar trend in which average polarity magnitude gradually increases from 24 to 32 hAPF and then decreases from 32 to 36 hAPF (Fig. 6C-E). Depolarization of core polarity protein occurs following the formation of trichomes at 32 hAPF (Usui et al., 1999; Merkel et al., 2014). By comparing each developmental time point to 24 hAPF, we found that the PCA method provided a higher statistical significance in detecting overall changes in polarity distribution when compared with the other methods (Fig. 6C-E). In support of this, we also computed the one-way ANOVA F-ratio, which is the ratio of variability between average polarity for each timepoint and variability within the timepoint for a given polarity method. The F-ratio obtained from the PCA method is higher than that obtained from the Ratio and Fourier Series methods, indicating that there is a higher statistical significance between average polarity magnitude for each timepoint (F-ratios are 4.7 for Ratio, 12.7 for Fourier Series and 15.9 for PCA). This could be attributed to the differences in polarization strength profile for different methods – both the Fourier Series and Ratio methods attained maximum polarization magnitude even when proteins are not fully segregated to the opposite vertical junctions (Fig. 2E). Moreover, both the Fourier Series and Ratio methods are affected by variation in cell eccentricities, reporting lower polarity magnitude for less eccentric cells and attaining maximum polarity magnitude at cell eccentricity around 0.5 for Ratio and 0.7 for Fourier Series (Fig. 2C). As cells become more regular in shape from 24 to 36 hAPF, cell eccentricity decreases from ∼0.6 to 0.2 (Fig. S1D). This results in lower polarity magnitude readout at later timepoints, thereby reducing the differences in polarity strength between earlier and later timepoints. This suggests that the PCA method is more sensitive and reliable in detecting changes in protein distribution accompanied with variation in cell geometry and protein distribution.

Next, we used the polarity readout from the PCA method to perform a broader polarity analysis during *Drosophila* pupal wing morphogenesis. Both the PCA vector average and neighbor average polarity magnitudes gradually increased from 24 to 32 hAPF and then decreased from 32 to 36 hAPF, displaying the same trend as the average polarity magnitude readout (Fig. 6F-H). However, average polarity strength at 34 hAPF was subtly greater than at 24 hAPF (Fig. 6E), although this difference is no longer significant when considering the vector and neighbor average polarity (Fig. 6G,H). This is because these vectorial measures capture both polarity strength and local polarity alignment, with the latter being low at both earlier and later timepoints. This is reflected in the polarity angle variance, which decreases from 24 to 32 hAPF as polarity alignment increases, then increases from 32 to 36 hAPF as polarity angles become more dispersed (Fig. 6I).

**Fig. 7.**
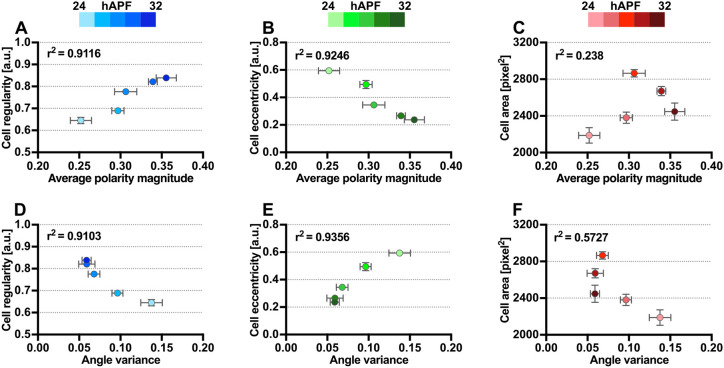
**Temporal correlation between cell size, regularity and eccentricity with Fz-EGFP polarity of wild-type wings.** (A-C) Fz-EGFP polarity magnitude is positively correlated with cell regularity (A), negatively correlated with cell eccentricity (B) and uncorrelated with apical cell area (C). (D-F) Fz-EGFP polarity angle variance is negatively correlated with cell regularity (D), positively correlated with cell eccentricity (E) and uncorrelated with apical cell area (F). Each dot represents the total average of averaged values from all wings for specific developmental timepoint. *n*=5 wings per timepoint. r^2^ indicates the coefficient of determination. Dot indicates mean, error bars show the s.e.m.

To understand the mechanism by which epithelial tissues develop specific packing geometries and coordinate their core planar polarity, we examined how cell size and shapes correlate with the strength of core protein polarization from 24 to 32 hAPF. Consistent with previous findings (Classen et al., 2005; Aigouy et al., 2010), the temporal progression of Fz-EGFP polarity magnitude strongly correlated with changes in cell regularity and eccentricity over these developmental timepoints. Average cell shape regularity and polarity magnitude were positively correlated (r^2^=0.9116), with more regular cells exhibiting higher polarity magnitude and vice versa (Fig. 7A). Similarly, average cell eccentricity and polarity magnitude were negatively correlated (r^2^=0.9246) (Fig. 7B). Interestingly, we found only a weak correlation between apical cell area and polarity magnitude during these developmental timepoints (r^2^=0.238) (Fig. 7C).

As wild-type wing tissue becomes more regularly packed and less eccentric, Fz reorients its polarity alignment to become increasingly coordinated along the proximodistal axis (PD axis) of the wing (Classen et al., 2005; Aigouy et al., 2010). Hence, we examined whether cell size, regularity and cell eccentricity correlated with core polarity alignment. Indeed, we found that polarity angle variance was strongly correlated with cell regularity and eccentricity (r^2^=0.9103 and 0.9356, respectively) but moderately correlated to apical cell area (r^2^=0.5727) from 24 to 32 hAPF (Fig. 7D-F). Thus, when using the PCA method as an accurate measure of planar polarization, we are able to conclude that Fz polarity does indeed increase as cells becomes more regular and less eccentric. We furthermore find negligible evidence for cell size influencing planar polarity at this stage of pupal wing development.

## DISCUSSION

Planar polarization is essential during morphogenesis for coordinating and organizing cells to establish specific tissues structures in a wide range of organisms. Hence, accurate and unbiased quantitative analysis of planar polarization is of paramount importance for deciphering molecular mechanisms underlying morphogenesis. Previous planar polarity quantification methods can be affected by variation in cell geometry. This study describes a novel method for quantifying asymmetrical localization of junctional proteins based on Principal Component Analysis (PCA). This method has been validated against existing polarity methods (the Fourier Series and Ratio methods) under various conditions using simulated cells. The simulation results revealed that the polarity readout from both the Fourier Series and Ratio methods are robust against variation in cell sizes and regularities but not cell eccentricities. The PCA method, on the other hand, consistently produces a polarity readout that is unaffected by variation of cell sizes, shapes and eccentricities. Additionally, the PCA method shows a more symmetric polarization strength profile when challenged with varying junctional protein distributions. The PCA method is also more reliable in detecting polarization strength due to variation in relative peak-to-base intensity.

All methods perform robustly in quantifying polarity on simulated images with varying brightness and signal-to-noise ratios. However, images with extremely low signal-to-noise ratio could interfere with polarity readout obtained from all methods.

Having validated these methods on simulated data, we tested their performance on various planar polarized epithelial tissues with distinctive cell geometries. Existing polarity methods correlate well with the PCA method on regular and less eccentric cell shapes. However, consistent with the simulation results, polarity readouts obtained from both the Fourier Series and Ratio methods are poorly correlated with the PCA method (and the published results) on highly elongated epidermal embryonic cells. Both simulation and experimental results demonstrate that the PCA method can be used reliably to quantify planar polarization independently of cell geometries.

To allow for automated and high-throughput analysis of cell polarity and shape, we further developed a standalone and user-friendly graphical user interface, QuantifyPolarity. This tool enables experimentalists with no prior computational expertise to perform comprehensive analyses of cellular and molecular mechanisms driving tissue morphogenesis. To demonstrates the application of QuantifyPolarity, we analyzed the temporal dynamics of cell behavior in the developing pupal wing. Here, we found that the temporal progression of core planar polarization magnitude is strongly correlated with cell regularity and eccentricity, consistent with a previous report (Aigouy et al., 2010). Although it is clear that correlation does not necessarily imply causation, it will be interesting to investigate the causality effect of cell shape on core planar polarization. Although it is known that apical cell area plays a role in affecting core planar polarity system, where Fz fails to restrict prehair initiation to the distal cell junctions in substantially larger cells (Adler et al., 2000), there is a lack of temporal correlation between core planar polarization and apical cell size of otherwise wild-type wings. This is likely due to the fact that these apical cell sizes in wild-type wings fall within the ‘normal’ range. Hence, it will be interesting to examine how considerably smaller or larger cell size affects the ability of core proteins to polarize.

Similarly, there is a strong temporal correlation between global polarity alignment, and cell regularity and eccentricity, but weaker correlation with apical cell area in wild-type wings. It has been proposed that irregular epithelial packing impairs the feedback propagation of polarization signal across the epithelium (Ma et al., 2008) and defective hexagonal packing leads to loss of global polarity coordination in the wing (Bardet et al., 2013). However, it has been reported that stretch-induced directional cell junctional rearrangement plays a role in coordinating global polarity alignment (Aigouy et al., 2010; Aw et al., 2016). Thus, polarity alignment may not be simply a consequence of cell geometry. An understanding of how different cell geometry quantitatively accounts for the underlying mechanism of core planar polarization can serve as a route towards elucidating molecular mechanisms of tissue planar polarization.

## MATERIALS AND METHODS

### Dissection and mounting of pupal wings for *in vivo* live imaging

All the fly strains used in this study are described in Table S1 and were raised at 25°C to the age indicated, without distinguishing between males and females. Pupae were dissected and mounted for *in vivo* live imaging as described previously (Classen et al., 2008) as live imaging is less susceptible to potential artefacts (e.g. noise from non-specific labeling or changes in tissue shape due to dissection and fixation). Briefly, pupae were placed on a piece of double-sided tape dorsal side up. Using a pair of fine scissors and forceps, the puparium case was carefully removed from above the developing pupae to expose the wing without injuring the pupa. The exposed pupal wing was covered in a drop of Halocarbon 700 oil and was then taped onto a 2.5 cm glass-bottomed dish (Iwaki) with the wing facing the coverslip. Subsequent imaging and processing steps are summarized in Fig. S4 and described below.

### Preparation of wing discs and embryos for fixed imaging

Wing discs were immunolabeled for Dachsous protein distribution and imaged as described previously (Hale et al., 2015). Embryos expressing Ubi::E-cadherin-GFP were fixed and imaged as described previously (Bulgakova et al., 2013).

### Image acquisition

Live image acquisition was performed using an inverted Nikon A1 confocal microscope with a Nikon 60× apochromatic objective lens oil (NA=1.4) and GaAsP detectors. The pinhole was set to 1.2 Airy Unit (AU). A heated stage was set to 25°C. To maintain constant power for all imaging sessions, laser power was checked and if necessary adjusted before each imaging session. For imaging of green emissions, a 488 nm laser with a 525-550 band pass filter was used to detect EGFP. Images were taken at the proximal-posterior region of the pupal wing with 1024×1024 pixels per *z*-slice and 80 nm pixel size. For each wing, 12-bit *z*-stacks (with ∼20 slices per stack, 0.5 μm/slice) were acquired. After time-lapse imaging, pupae were kept and survived to at least pharate stage and >95% to eclosion stage.

### Image processing

Raw microscopy images were first processed using external tools (e.g. PreMosa and PackingAnalyzer) to obtain skeletonized representation of the cell boundaries (also known as segmented images). These segmented images along with their original images are the pre-requisite inputs in QuantifyPolarity GUI for further image analysis.

#### Image surface extraction (PreMosa)

For image processing, microscopy images were exported into TIFF format using Nikon software (NIS-Elements AR) for further processing. These *z*-stack images were automatically surface extracted and projected using PreMosa as described previously (Blasse et al., 2017) to obtain a 2D projected image of the apical band of monolayer epithelial tissues (Fig. S4A). To quantify proteins localizing to the apical junctions, Fz-EGFP *z*-stack images were used to generate the height map. In brief, this algorithm generates an initial height map that contains information of each *z*-slice with the brightest pixels. To yield a smooth and optimized height map, smoothing (with a median filter) and artefact correction processes were carried out. The final height map was used to project the manifold of interest onto a 2D image.

More commonly used image surface extraction methods, such as maximum intensity projection, are available in Fiji. This step can be omitted for single *z*-slice image acquisition.

#### Image segmentation (PackingAnalyzer)

To identify epithelial cell boundaries, the image was segmented using the cell segmentation software PackingAnalyzer (Aigouy et al., 2010). Cell segmentation software such as PackingAnalyzer is used to identify cell boundaries using a watershed algorithm (Aigouy et al., 2010). This procedure identifies and produces a binary skeletonized representation of the cell boundaries for further image analysis (Fig. S4A). Additional manual correction was often required to obtain precise segmentation of cell boundaries. Thus, all segmented images were checked and corrected manually for segmentation errors such as under-segmentation and over-segmentation. Boundary cells and small cells are automatically removed based on the area thresholds set by the user, which vary according to the image size and specifications. This is because boundary cells do not contain all the cell edges; therefore, it is not possible to quantify the morphological properties of boundary cells. These segmented images are then passed onto the QuantifyPolarity GUI for further image analysis.

### Identification of cells and neighbor relations

A series of steps was then employed to extract information out of the segmented images. Each cell was labeled with a unique identification number (Fig. S4B). A vertex was determined by calculating the vertex degree, which gives information on the number of edges attached to one vertex. By going through the 4-connectivity binarized image, the sum of pixels within the 3×3 neighborhood of each foreground pixel (for a binary image, 1 is the foreground pixel and 0 is the background pixel) is determined. Therefore, the vertex degree *k* may be written as(1)
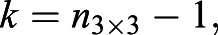
where *n*_3*×*3_ is the number of foreground pixels in the 3×3 neighborhood.

From a biological point of view, a vertex is where multiple (i.e. three or more) edges meet. Therefore, *k* has to be bigger or equal to 3 in order to be considered as a vertex point. This results in a polygonal lattice of cells, in which each of the polygons consists of a unique set of vertices and edges that are crucial for further cell shape and topology analysis, as well as for polarity quantification.

We then identified the immediate neighbors for each individual segmented cell. This step serves as a pre-requisite for the measurement of neighbor vector polarity.

### Quantification of planar polarization using the Fourier Series method

Planar polarity quantification based on the Fourier Series method is implemented as described previously (Aigouy et al., 2010; Merkel et al., 2014). Using the intensity of junctional proteins, the symmetric tensor components *Q*_1_ and *Q*_2_ are computed as described (see Eqns 3 and 4 in Aigouy et al., 2010). In order to allow for polarity comparison between images, *Q*_1_ and *Q*_2_ are normalized to normalization constant *N*, where 
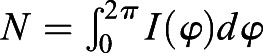
, as described by Merkel et al. (2014):(2)
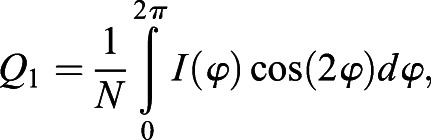
(3)
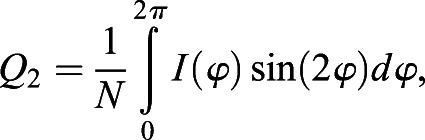
where 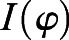 is the intensity of junctional proteins at segmented cell boundary at an angle 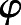 with respect to the centroid of the cell. The magnitude and angle of polarity in each cell are defined as described in Eqn 5 in Aigouy et al. (2010).

In the Fourier Series method, polarity magnitude starts from 0 onwards, with 0 having complete zero polarization, while increasingly values represent increasing polarization. All polarity angles (in degrees) range between −90° and +90°, with 0° corresponding to the *x*-axis of the image. To allow direct comparison between all polarity methods, we computed the normalized Fourier Series polarity magnitudes by normalizing against its maximum value.

### Quantification of planar polarization using the Ratio method

Planar polarity quantification based on the Ratio method described previously (Strutt et al., 2016) is implemented and improved as follows. Within each cell, junctional protein intensity is grouped into four bins of equal size (90°). The binned data are then smoothed out using linear interpolation. The mean intensity that falls within the opposing pair of bins are summed up and the ratio between the bin pairs is denoted as asymmetry. All the asymmetries are rounded to a precision of 10^−3^. Thus, the maximum asymmetry is the polarity magnitude. The central angle that corresponds to the average of all angles from multiple maximum asymmetries is considered as the polarity angle.

In the Ratio method, polarity magnitude starts from 1 onwards, with 1 having complete zero polarization, while an increasing value represents increasing polarization. All polarity angles range between −90° and +90°, with 0° corresponding to the *x*-axis of the image.

The normalized Ratio polarity magnitude of a cell is computed by subtracting 1 from the ratio value and normalizing it against the maximum of all the subtracted ratio values from all cells. This allows for direct comparison between all polarity methods.

### Quantification of planar polarization using a PCA-based method

Here, we implemented a Principal Component Analysis-based method to quantify 2D planar polarization. In order to compensate for elongated cells, the cell is negatively stretched (or compressed). Each pixel on the cell boundary is represented as (*x*_*i*_, *y*_*i*_, *I*_*i*_), where *x*_*i*_, *y*_*i*_ represent the *x*- and *y*-coordinates of the pixel, and *I*_*i*_ represents the intensity of that pixel. An ellipse is fitted to obtain the semi-major and semi-minor axes, *a* and *b*, and the orientation, 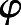, of the cell. For each of these points, it undergoes the following transformation:(4)
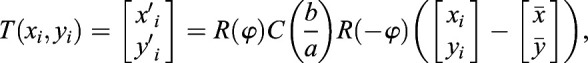
where 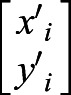 is the transformed (compressed) coordinates relative to the cell centroid 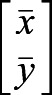, 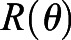 is the rotation matrix with the rotation angle of *θ*, and 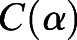 is the compression matrix with the compression factor *α*, with *α*<1. Both of these matrices can be written as(5)
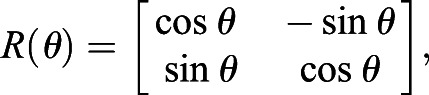
(6)
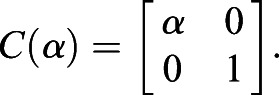


Although this operation reduces the cell area, it has been shown in Fig. 2A,A′ that polarity readout is not affected by cell area.

Next, angle *θ*_*i*_ is computed based on the transformed coordinates 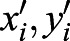, with respect to the centroid of the cell. In order to mitigate the effect of the denseness of points on the calculation of the covariance matrix, the weighting *dθ*_*i*_ for each *i* point on the cell boundary is calculated as follows:(7)
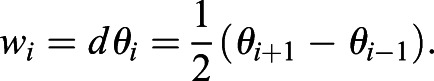
For each point *i* on the cell boundary with intensity *I*_*i*_, all the intensities are normalized so that it is independent of the image format (e.g. 8-bit, 12-bit and 16-bit) and brightness.(8)
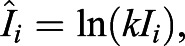
where *k* is the normalization factor (≈ 10^3^ empirically). When varying total amount of protein intensities in a simulated cell, polarity magnitude obtained from the PCA method is slightly reduced by 0.9% with decreasing base intensity (<10 a.u.) (Fig. S2A′).

Instead of positional *xy*-coordinates, we used the intensities as the distances from the centroid at specific angles, which are then converted into cartesian coordinates using trigonometric functions to obtain transformed coordinates 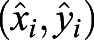, as follows:(9)
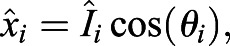
(10)
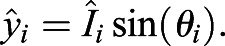


Next, the covariance matrix, ***σ***, is calculated as follows:(11)
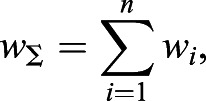
(12)
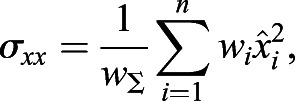
(13)
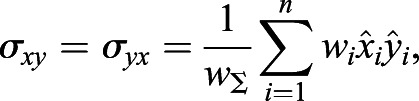
(14)
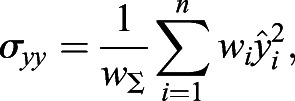
(15)
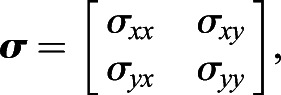
where *w*_Σ_ is the sum of all weightings and *σ*_*xx*_, *σ*_*yy*_, *σ*_*xy*_ and *σ*_*yx*_ are the covariances.

Eigenvalues *λ*_1_, *λ*_2_ with *λ*_1_≥*λ*_2_ and eigenvectors 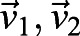 of the covariance matrix ***σ*** are computed accordingly. The eigenvalues *λ*_1_, *λ*_2_ have several properties: (1) when the protein distribution is homogeneous, *λ*_1_=*λ*_2_; (2) the greater the bipolarity, the greater the difference between *λ*_1_ and *λ*_2_.

Using the eigenvalues and covariances, we defined the magnitude of polarity *p* and the angle of polarity *θ* for a single cell as(16)
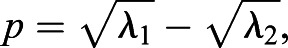
(17)
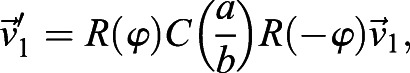
(18)
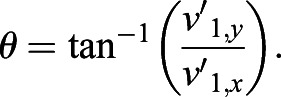
Polarity magnitude obtained from the PCA method starts from 0 onwards, with 0 having complete zero polarization, while increasing values represent increasing polarization. The angle of polarity *θ* is measured with respect to the *x*-axis of an image. Polarity angle measurement ranges between −90° and +90°, with 0° oriented along the *x*-axis and ±90° oriented along the *y*-axis. To allow direct comparison between all polarity methods, we computed the normalized PCA polarity magnitudes by normalizing against its maximum value.

### Quantification of planar polarization at tissue scales

The cell-by-cell polarity readout obtained from either method is further applied to measure local (coarse-grain and neighbor) polarization (Fig. S4B′, parts ii and iii). Within a group of cells, the polarity measurements can be combined in specific ways to reveal the strength of polarity, as well as the polarity coordination between cells. The most direct way is to compute the average of polarity magnitude without taking its polarity angle into consideration, which is termed the ‘Average Polarity Magnitude’. This measure gives us an idea of the average polarization strength for all cells within an image field. It can be simply computed using the following equation:(19)
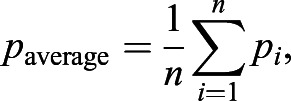
where *p*_*i*_ is the polarity magnitude of *i*^th^ cell and *n* is the total number of cells.

For the described simulation and experimental data, we used the ‘Average Polarity Magnitude’ measure as a simple readout of polarity strength. On the other hand, ‘vector’ polarity measurement is defined to capture the strength and coordination of planar polarity between groups of cells within an image field (referred to as ‘Vector Average Polarity’), between groups of cells within a defined area (‘Coarse-Grain Vector Polarity’) and with its immediate neighbors (‘Neighbor Vector Polarity’). First, polarity of individual cells is converted into their vector form 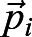. The vector polarity, 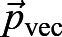, is computed as follows:(20)
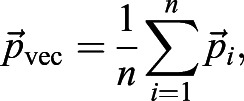
(21)
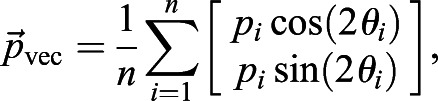
where 
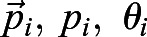
 are the polarity vector, polarity magnitude and polarity angle of *i*^th^ cell, respectively. *n* is the total number of cells. Therefore, vector average polarity magnitude *p*_vec_ and angle *θ*_vec_ can be explicitly written as:(22)
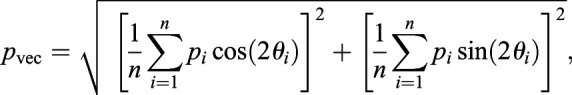
(23)
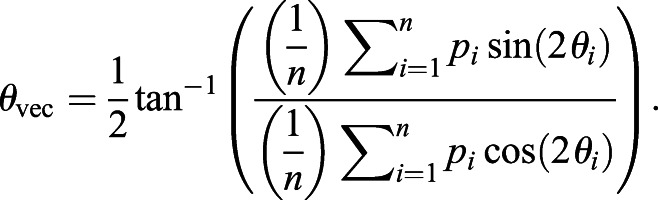
Notice that the computed vector average polarity magnitude *p*_vec_ takes the polarity angle of individual cells into consideration. Therefore, *p*_vec_≤*p*_average_ for all possible cases of polarity magnitudes and angles, with equality if and only if all polarity angles are equal.

To visualize the coarse-grain polarity on the scale of groups of cells or the entire image field, cells are divided into groups with equal number of cells. For coarse-grain polarity of the entire image field, the vector average polarity *p*_vec_ and angle of polarity *θ*_vec_ for all cells within the image field are computed as described in Eqns 22 and 23. For coarse-grain of a group of cells, the vector average magnitude *p*_vec_ and angle of polarity *θ*_vec_ for a group of cells are computed as described in Eqns 22 and 23 (Fig. S4B′, part ii). On the other hand, to capture local polarity coordination of individual cells with their immediate neighbors, the immediate neighbors of each cell are identified and computed for vector average magnitude *p*_vec_ and angle of polarity *θ*_vec_ as described in Eqns 22 and 23 (Fig. S4B′, part iii). The neighbor vector polarity magnitudes obtained from all the cells are averaged across the tissue to obtain the average neighbor vector polarity magnitude measure.

Apart from that, circular statistics are used to quantify the degree of alignment or coordination of polarity angle between cells. A measure called circular angle variance, as implemented in CircStats MATLAB toolbox (Berens, 2009), is used to determine the circular spread of vectorial data. In order to accommodate the rotational symmetry of polarity angle, the angle variance Var_circ_ for polarity angles can be computed as follows:(24)
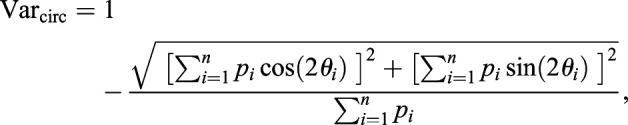
where *θ*_*i*_ is the polarity angle of *i*^th^ cell and *n* is the total number of cells. Angle variance ranges between 0 and 1, with 0 having complete agreement in polarity alignment, while 1 represents complete polarity misalignment.

### Mean angle difference 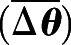


Additionally, we computed the mean angle difference, 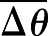, as a way to quantitatively compare the differences in polarity angles obtained from the PCA method against the Ratio and Fourier Series methods. Briefly, this is carried out by first determining the difference in the polarity angles obtained from two different methods for each individual cell, accommodating the circular spread of the data. We then computed the mean of the (absolute) polarity angle differences for all cells across the image, with 0° representing complete agreement between polarity angles obtained by two different methods, while higher values indicate that the methods agree less.

### Circular weighted histogram

To visually compare cell polarity angle obtained from different planar polarity methods, we plotted a circular weighted histogram described as follows. First, we computed the magnitude and angle/axis of polarity on a cell-by-cell basis using all three methods. Data from multiple wings were combined and represented by a circular weighted histogram using the MATLAB built-in function ‘*polarhistogram*’. Data were grouped into 20 bins, with each bin representing a unique polarity angle. Histograms were weighted by the average magnitude of polarity within each bin to capture both the angle and magnitude of polarity (Aw et al., 2016). The length of each bin represents the polarity magnitude-weighted frequency of occurrences; meanwhile, the orientation of each bin represents the axis of average polarity. The angle of polarity has characteristic rotational symmetry; hence, a polarity angle of *θ* also corresponds to *θ*+*π*. Therefore, for better visual representation, all computed polarity angles (ranges from −90° and +90°) were replotted in a range between 0° and 360°, where 0° corresponds to the *x*-axis of the image.

### Cell morphological parameter measurements

#### Cell area and perimeter

The apical cell area (pixels^2^) and perimeter (pixels) for each cell was determined from the labeled images using the MATLAB built-in function ‘*regionprops*’.

#### Cell shape regularity

Cell shape regularity was quantified based on how ‘far’ the shape of a cell is from a regular polygon, using a measure focusing on the equilateral and equiangular properties of a polygon (Chalmeta et al., 2013). From the lengths of the edges (*l*_*i*_), first the median length of edges (*l*_*median*_) and the sum of all edge lengths (*l*_Σ_) were determined. Then, an intermediate term *D* can be calculated as follows:(25)
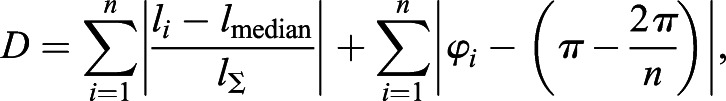
where *n* is the number of sides and 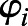 are the interior angles of a cell. Finally, the cell shape regularity measure *μ* can be obtained as follows:(26)
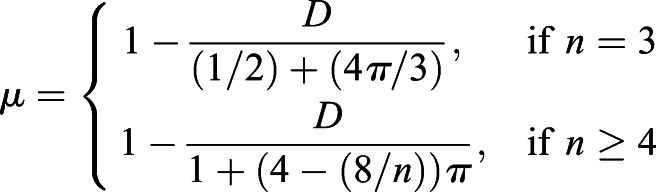
The value of cell regularity (a.u.) ranges from 0 to 1, with 0 representing highly irregular and 1 being perfectly regular with equal length of cell edges and interior angles.

#### Cell eccentricity and orientation

To measure cell eccentricity, a robust ellipse-fitting approach was used. It is a shape-based method where the cell boundaries are used as a reference landmark for ellipse fitting (Young, 2010). Any ellipse can be described by the following (general) equation:(27)
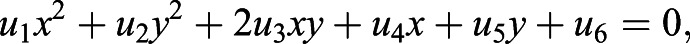
where *u*_*i*_ is the unique coefficients of each distinct ellipse and (*x*, *y*) are the coordinates of the cell boundaries. The least squares method is used to determine the most optimal set of coefficients, *u*_*i*_, for every single cell. The parameters of an ellipse can then be determined using the following equations:(28)
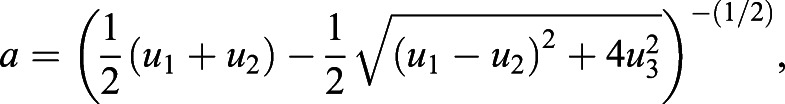
(29)
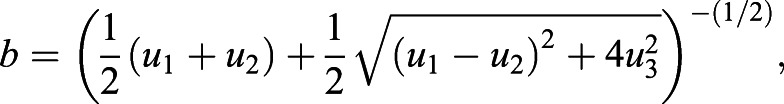
(30)
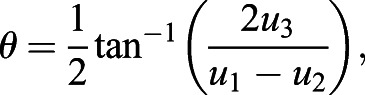
where *a*, *b* are the semi-major and semi-minor axes, respectively, with *a*≥*b*, and *θ* representing the cell orientation. The value for cell orientation ranges from −90° to +90°, with 0° corresponding to the *x*-axis of the image.

By fitting an ellipse onto the geometry of a cell, the eccentricity can be calculated using the following formula:(31)
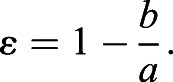
The value for cell eccentricity ε ranges from 0 to 1 in arbitrary units, with 0 representing no elongation (or circular) and 1 being highly eccentric.

#### Number of cell junctions

A cell junction that is below 10% of average junctional length will not be considered as a side of a cell. Once cell junctions are defined, the number of cell junctions, which is equivalent to the number of neighboring cells, can be determined.

### Statistical analysis

Quantification measures, such as polarity magnitude, cell regularity, cell eccentricity and cell apical area for each genotype or timepoint were averaged and tested for normality with the Shapiro-Wilk test. The resultant data from different genotypes or timepoints (*n*, number of samples) were compared using an unpaired one-way ANOVA test. If it was statistically significant, a post-hoc test called Tukey-Kramer's multiple comparison was used to compare to a specific group within an experiment (for comparison between two groups, an unpaired *t*-test was used). In some experiments, coefficient of determination test (r^2^) was computed to determine how strong the correlation was between two variables. In all graphs, error bars indicate s.e.m., unless otherwise mentioned.

## Supplementary Material

Supplementary information

Reviewer comments
