# Supplementary material for: QuantifyPolarity, a new tool-kit for measuring planar polarized protein distributions and cell properties in developing tissues
Source: Development. 2021 Sep 7;148(18):dev198952. doi: 10.1242/dev.198952 (PMC8451067; doi:10.1242/dev.198952)
Supplement: Supplementary information [file develop-148-198952-s1.pdf]

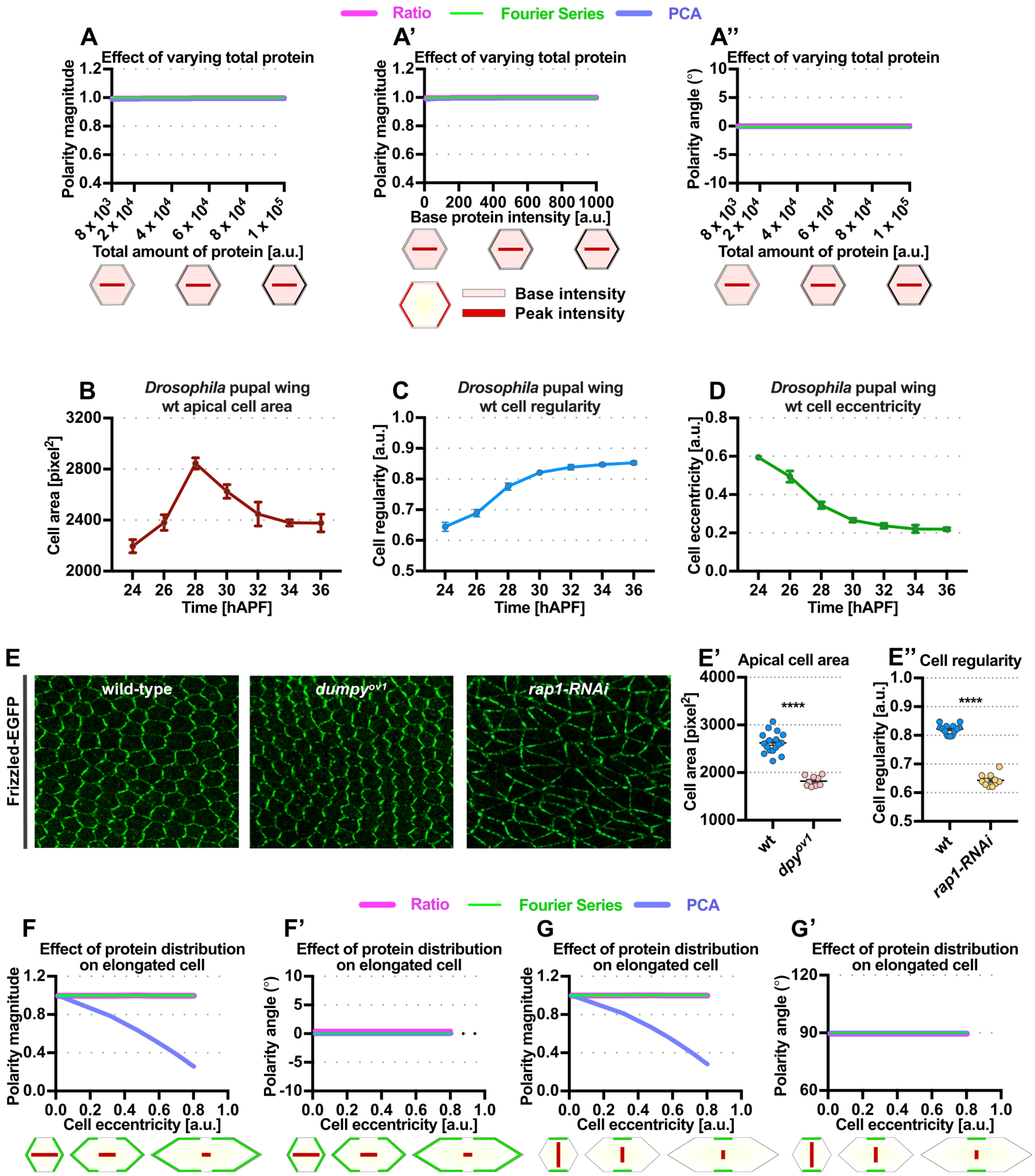

Fig. S1. The variation of cell size and shape during pupal wing development and in different genotypes

(A-A'') Effects of varying total amount of proteins on the entire cell junctions with conserved cell geometries, junctional protein distribution and relative peak-to-base intensity levels. Graphs show quantified normalized polarity magnitudes against varying (A) total amount of protein on all junctions of the cell and (A') base protein intensity. (A'') Quantified polarity angles obtained from varying total amount of protein on all junctions of the cell.

Development • Supplementary information

All polarity magnitudes obtained using different methods are normalized to allow comparison unless otherwise stated. All polarity angles range between -90° to +90°, with 0° corresponding to the x-axis of the image.

(B-D) Quantification of average (B) apical cell area, (C) cell regularity, (D) cell eccentricity of otherwise wild-type pupal wings expressing Fz-EGFP from 24 to 36 hAPF ($n = 5$ wings analyzed).

(E) Confocal images of otherwise wild-type, *dumpy^{ov1}* mutant and *rap1-RNAi* pupal wings expressing Fz-EGFP at 30 hAPF. *dumpy^{ov1}* mutant wings lack the extracellular matrix protein Dumpy required to regulate proper wing shape and size development. *rap1-RNAi* wings lack the homogeneous distribution of E-Cadherin required for regulation of cell shape. Note distinctive sizes and shapes of planar polarized cells on these mutant backgrounds as compared to otherwise wild-type cells.

(E’) Quantified average apical cell area of *dumpy^{ov1}* and wild-type wings at 30 hAPF ($n = 11$ - 13 wings per genotype analyzed).

(E’’) Quantified average cell regularity of *rap1-RNAi* and wild-type wings at 30 hAPF ($n = 11$ - 13 wings per genotype analyzed). Error bars indicate mean$\pm$SEM. Unpaired t-test. Significance levels: p-value ≤ 0.0001^{****}.

(F-F’) Quantified polarity magnitudes (F) and angles (F’) of simulated cells with varying junctional peak protein distribution and cell eccentricity, from 0 to 0.8.

(G-G’) Quantified polarity magnitudes (F) and angles (F’) of simulated cells with varying junctional base protein distribution and cell eccentricity, from 0 to 0.8.

Development • Supplementary information

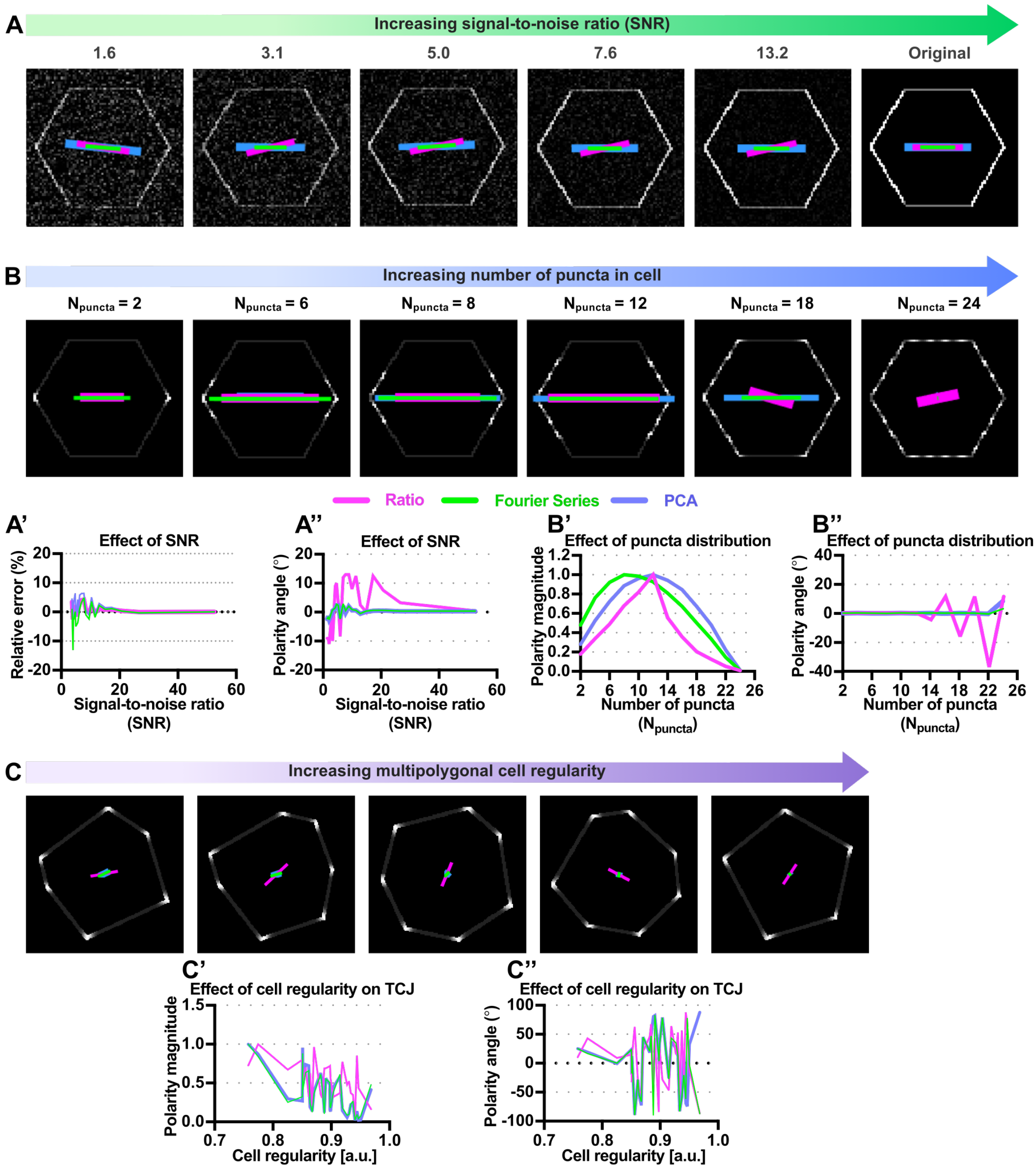

Fig. S2. Comparison of methods under conditions of increasing signal-to-noise ratio, punctate protein distributions and tricellular junction protein localization

(A) Simulation of regular hexagonal cells with fixed amount of proteins on both the vertical cell junctions. For the original noise-free cell, the peak and base intensities are set to 900 and 450 a.u. respectively. Random normally-distributed noise was artificially added into the original noise-free simulated cell. The signal-to-noise ratio (SNR) of each noise-added image was estimated using the original noise-free image (Gonzalez and Woods, 2007). The magenta (Ratio), green (Fourier Series) and blue (PCA) bars represent the magnitude

Development • Supplementary information

(length of bar) and angle (orientation of bar) of planar polarization for a given cell obtained from the Ratio, Fourier Series and PCA methods respectively.

(A'-A'') Graphs show quantified (A') polarity magnitudes and (A'') polarity angles of cells with varying image signal-to-noise ratios.

(B) Simulation of cells with increasing number of puncta (N_{puncta}) while maintaining cell geometry and puncta intensity. Total number of junctional protein puncta increase gradually (from 2 to 24) starting from both vertical cell poles. N_{puncta} = 12 indicates there are a total of 12 puncta in the simulated cell, with 6 puncta equally distributed starting from both poles of vertical junctions.

(B'-B'') Graphs show quantified (B') polarity magnitudes and (B'') polarity angles of cells with varying junctional puncta distribution.

(C) Simulation of tricellular junction localization on cells with varying shape regularity, where each tricellular vertex exhibits a fixed junctional puncta profile of a Gaussian function (intensities value ranges from 40 to 255 a.u.). Other bicellular junctions exhibit intensity of 40 a.u.

(C'-C'') Graphs show quantified (C') polarity magnitudes and (C'') polarity angles of tricellular localization on cells with varying shape regularity. Note changes in polarity readouts with varying cell regularity.

All polarity magnitudes obtained using different method are normalized to allow comparison unless otherwise stated. All polarity angles range between -90° to +90°, with 0° corresponding to the x-axis of the image.

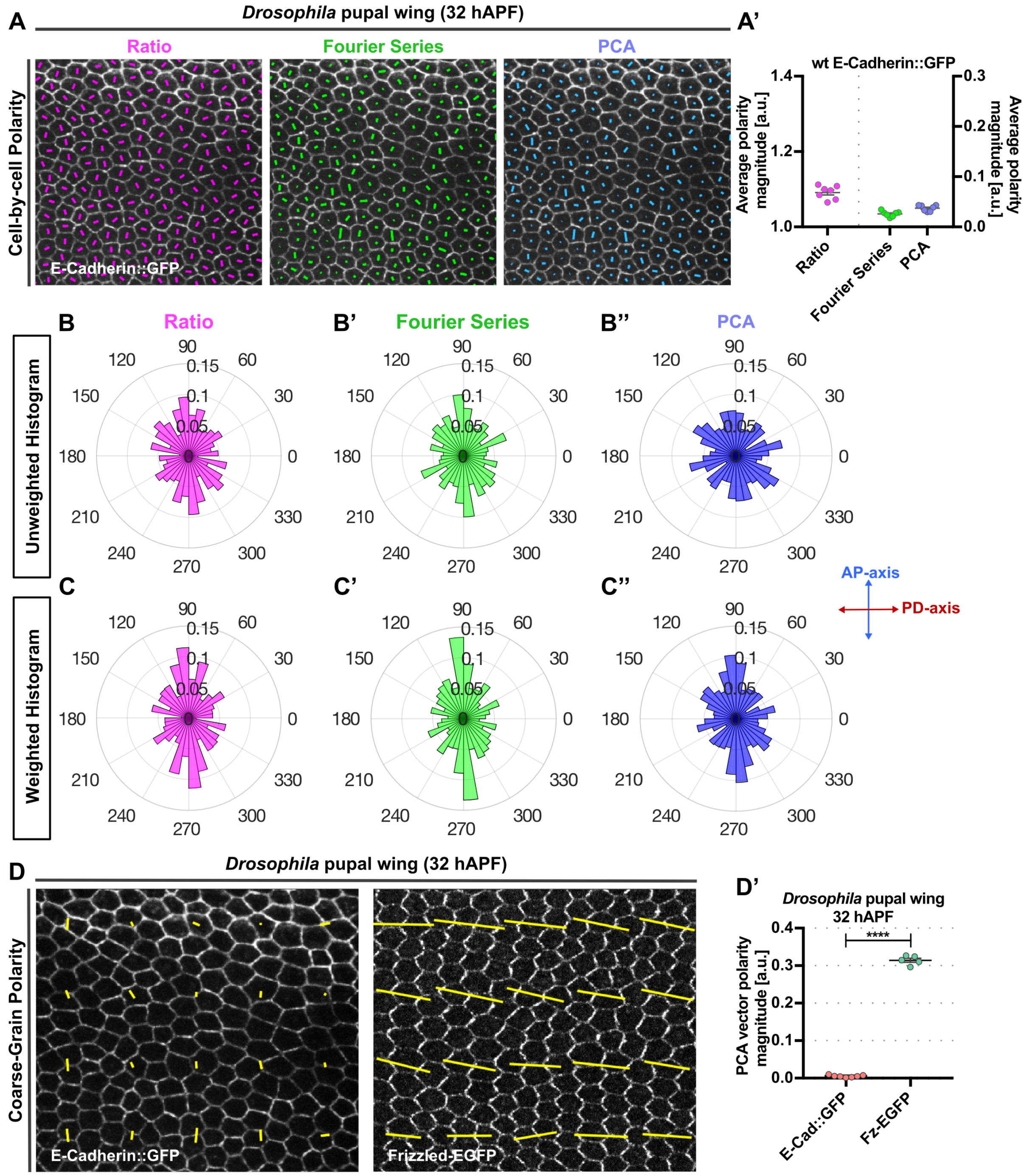

Fig. S3. The weakly polarized distribution of E-Cadherin on cell junctions results in low polarity magnitude with dispersed polarity angles

(A) Quantified cell-scale polarity pattern of otherwise wild-type wings expressing E-Cadherin::GFP at 32 hAPF using three different methods. The magenta (Ratio), green (Fourier Series) and blue (PCA) bars represent the magnitude and angle of planar polarization pattern for a given cell.

(A’) Plot of average polarity magnitudes at 32 hAPF obtained from the Ratio, Fourier Series

Development • Supplementary information

and PCA methods respectively ($n = 7$ wings analyzed). Error bars indicate mean$\pm$SEM.

(B-B'' and C-C'') Circular (B-B'') unweighted and (C-C'') weighted histogram plots displaying the orientation of E-Cadherin::GFP polarity obtained from Ratio, Fourier Series and PCA methods ($n = 7$ wings analyzed). AP- and PD-axes indicate anteroposterior- and proximodistal-axes respectively.

(D) Quantified coarse-grain polarity pattern of otherwise wild-type wings expressing E-Cadherin::GFP and Fz-EGFP at 32 hAPF. The yellow bars represent the magnitude (length of bar) and angle (orientation of bar) of planar polarization pattern for local group of cells obtained from the PCA method.

(D') Plot of coarse-grain vector polarity magnitude for E-Cadherin::GFP and Fz-EGFP expressing wild-type wings at 32 hAPF. Error bars indicate mean$\pm$SEM. Unpaired t-test. Significance levels: p-value ≤ 0.0001^{****}.

Development • Supplementary information

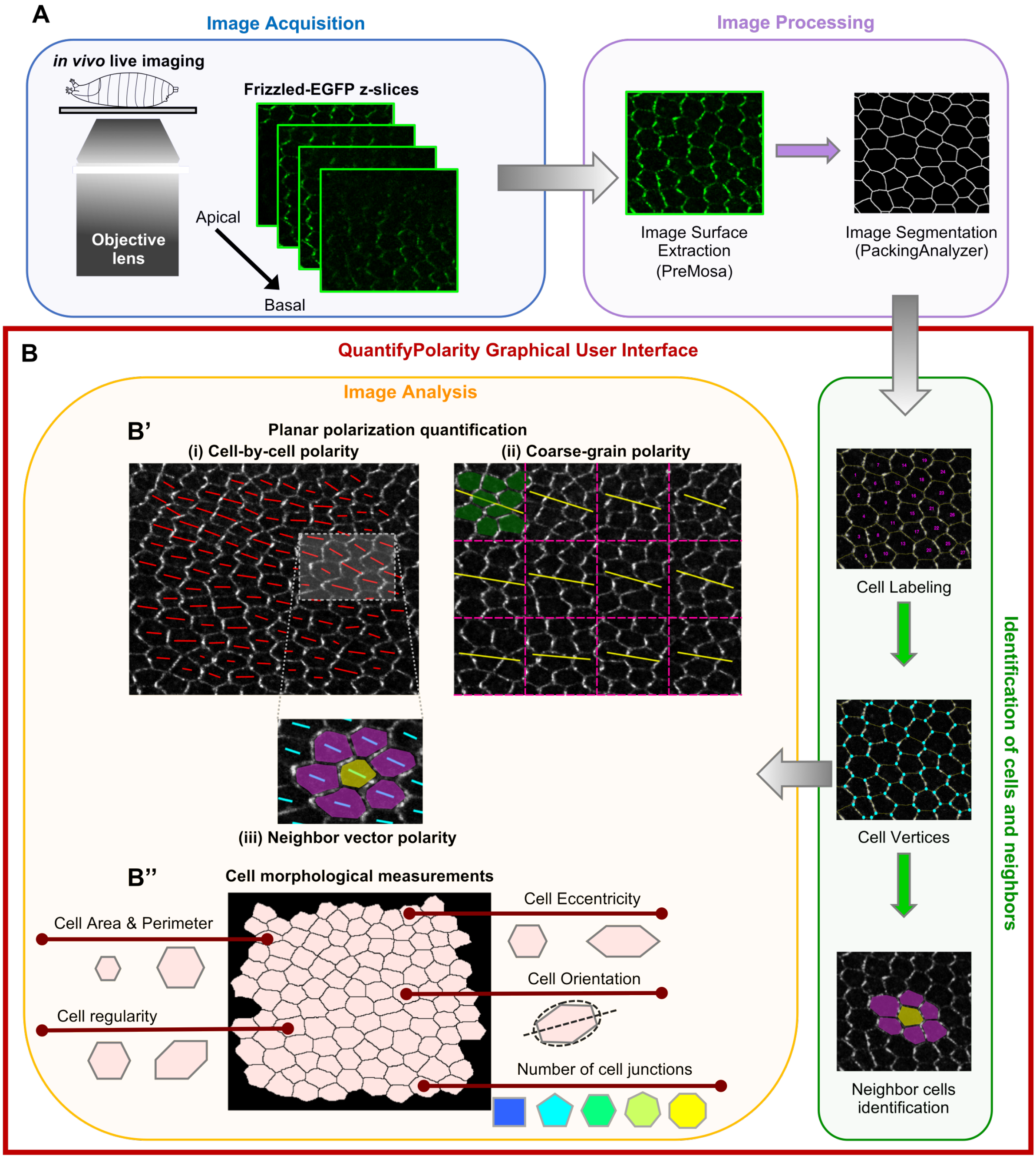

Fig. S4. Overview of image acquisition, processing and subsequent image analysis (in QuantifyPolarity) steps

(A) Following image acquisition, raw images are processed using external tools (e.g. PreMosa and PackingAnalyzer) to obtain segmented images. These images are then fed into the QuantifyPolarity GUI (red box).

(B) Following identification of cells and their neighbor relations (green box), QuantifyPolarity performs further image analysis (orange box), such as cell polarity and morphological quantifications.

Development • Supplementary information

(B’) Quantification of planar polarity at cellular and tissue scales.

(i) Cell-by-cell polarity pattern of a *Drosophila* pupal wing expressing Fz-EGFP at 30 hAPF. The length and orientation of red bars denote the polarity magnitude and angle for a given cell respectively.

(ii) Coarse-grain pattern of vector average polarity at 30 hAPF. Image is divided into group of cells with equal square grids (with dotted magenta lines), where the vector average polarity for each group of cells is computed. For each group of cells, the average polarity magnitude p_{vec} is proportional to the length of the yellow bar, while the average polarity angle θ_{vec} is denoted by the orientation of the yellow bar.

(iii) Neighbor vector polarity quantification for the yellow cell with its immediate neighbors (magenta cells). The length and orientation of cyan bars denote the neighbor polarity magnitude and angle for each cell.

(B’’) Cell morphological measurement tools are available in the QuantifyPolarity GUI to quantify cell area, perimeter, shape regularity, eccentricity, orientation and number of cell junctions.

Development • Supplementary information

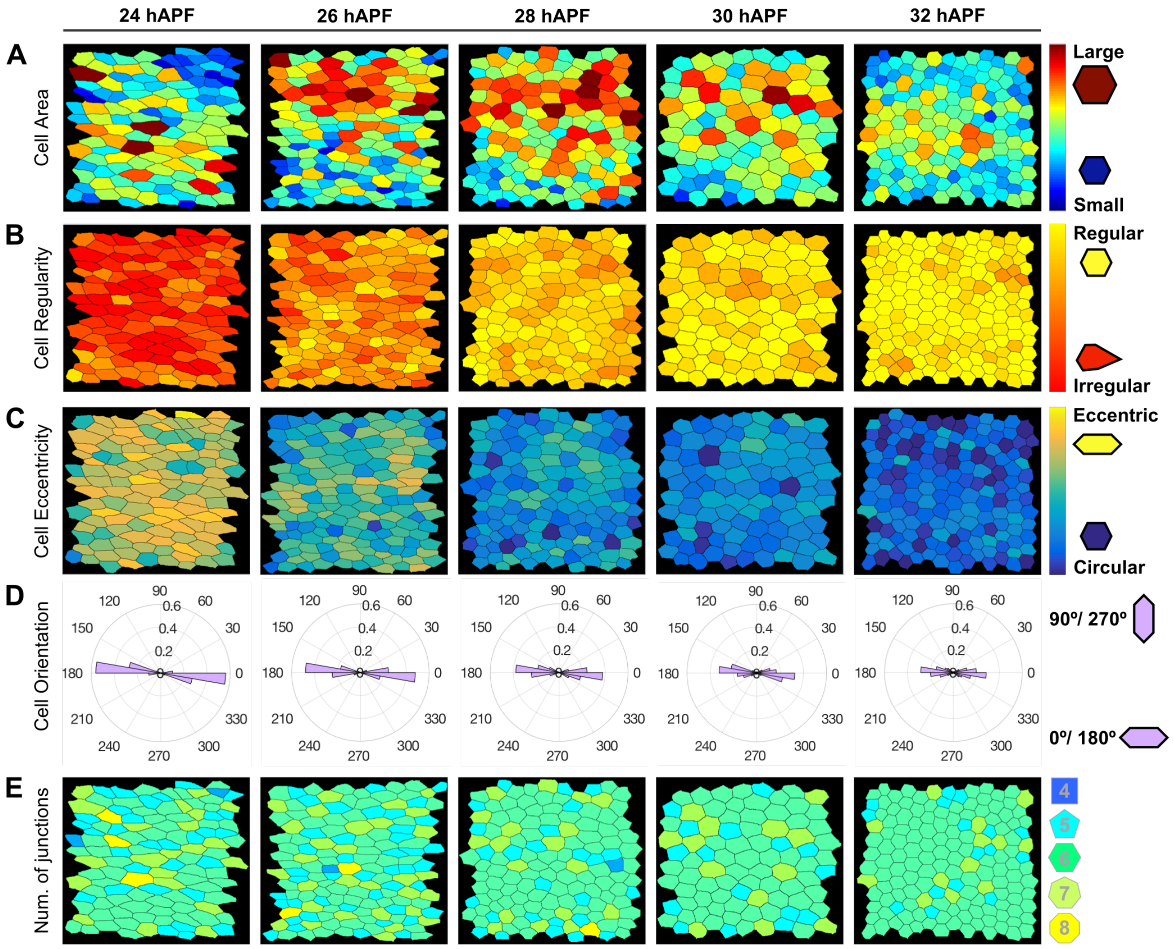

Fig. S5. Examples of cell morphological quantitative analysis of *Drosophila* pupal wing development computed using the QuantifyPolarity GUI

(A-E) Processed images of the posterior-proximal region of otherwise wild-type pupal wing from 24 to 32 hAPF.

(A) Cells are color-coded according to the cell apical area, with red represent cells with larger apical area and blue represents cells with smaller apical area.

(B) Cells are color-coded according to the regularity of the shape, with yellow being perfectly regular and red represent highly irregular.

(C) Cells are color-coded according to the eccentricity of the shape, with yellow represent highly eccentric and blue being circular or non-eccentric.

(D) Circular histogram plots display the orientation of cell, ranges between 0° to 360°, with 0°/180° corresponding to the x-axis of the image.

(E) Cells are color-coded according to the number of cell junctions.

Development • Supplementary information

	Case 1	Case 1.5	Case 2	Case 3
Examples of polarized tissues<br><br>The length and orientation of red bars denote the polarity magnitude and angle of individual cells	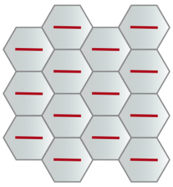	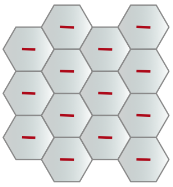	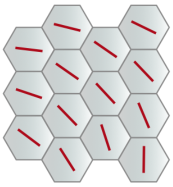	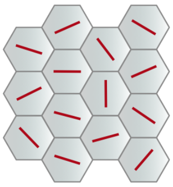
Individual cell polarity	Individual cells are strongly polarized	Individual cells are weakly polarized	Individual cells are strongly polarized	Individual cells are strongly polarized
Average polarity magnitude	Computes the average individual cell polarity magnitudes within the tissue<br><br>Case 1 = Case 2 = Case 3<br>Case 1.5 << (Case 1, 2 & 3)			
Local polarity<br>(with immediate neighbors)	Polarity is locally coordinated with neighboring cells	Polarity is locally coordinated with neighboring cells	Polarity is locally coordinated among neighboring cells	Loss of local polarity coordination
Average neighbor vector polarity magnitude	This measure allows us to capture the local polarity strength and coordination with neighboring cells<br><br>Case 2 < Case 1<br>Case 3 << (Case 1 & 2)			
Global polarity<br>(Tissue level)	Global coordination of polarity is observed (All the cell polarities are well-aligned along a single direction across the tissue)	Global coordination of polarity is observed	Loss of global coordination (No single polarity alignment across the tissue)	Loss of global polarity coordination
Vector average polarity magnitude	This measure captures the global polarity strength and coordination across the tissue<br><br>(Case 2 & 3) << Case 1			
Angle variance	This measure captures the polarity angle alignment (weighted with polarity magnitude) across the tissue. Angle variance ranges between 0 and 1, with 0 having complete agreement in polarity alignment, while 1 represents complete polarity misalignment<br>Case 1 = Case 1.5<br>(Case 1 & 1.5) << (Case 2 & 3)			
Coarse-grain vector polarity<br><br>The length and orientation of blue bars denote the vector average polarity magnitude and angle for each group of cells	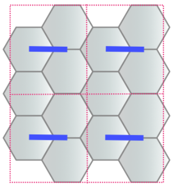	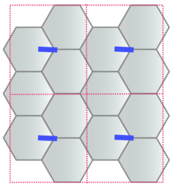	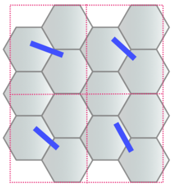	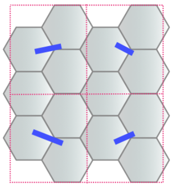

= denotes equal to; < denotes slightly less than; << denotes significantly less than.

Fig. S6. Different polarity measurements to quantify polarization at different scales (cellular, local and global)

Development • Supplementary information

Table S1. Key resources

RESOURCE	SOURCE	IDENTIFIER	ASSOCIATED FIGURES
**Experimental models: Organisms/Strains**			
fz-EGFP	(Strutt et al., 2016)	FBti0206968	Fig 1, Fig 3, Fig 6-7, Fig S1, Fig S3-S4
engrailed-GAL4	Bloomington Drosophila Stock Center	FBal0052377	Fig S1
y v; P{y+, v+, UAS-Rap1-RNAi[HMJ21898]}attP40	Bloomington Drosophila Stock Center	FBal0300407	Fig S1
dumpy^{ov1}	Bloomington Drosophila Stock Center	FBal0002834	Fig S1
E-Cadherin::GFP	Suzanne Eaton (Huang et al., 2009)	FBal0247908	Fig S3
Ubi::E-cadherin-GFP	(Oda and Tsukita, 2001)	FBal0122908	Fig 4
**Antibodies**			
Rabbit anti-Dachsous, affinity purified	(Strutt and Strutt, 2002)	N/A	Fig 4
**Software/Graphical User Interfaces**			
NIS Elements AR version 4.60	Nikon	N/A	N/A
PackingAnalyzer version 8.5 Beta	(Aigouy et al., 2010)	N/A	N/A
MATLAB_R2016b	MathWorks	N/A	N/A
GraphPad Prism version 7.0c	GraphPad software	N/A	N/A
QuantifyPolarity	This work	N/A	Fig 5
**Reagent**			
Halocarbon 700 oil	Halocarbon products	CAS: 9002-83-9	N/A

Development • Supplementary information

Supplementary References

Aigouy, B., Farhadifar, R., Staple, D. B., Sagner, A., Roper, J. C., Julicher, F. and Eaton, S. (2010). Cell flow reorients the axis of planar polarity in the wing epithelium of Drosophila. *Cell* **142**, 773-786.

Gonzalez, R. C. and Woods, R. E. (2007). Image processing. *Digital image processing* **2**.

Huang, J., Zhou, W., Dong, W., Watson, A. M. and Hong, Y. (2009). Directed, efficient, and versatile modifications of the Drosophila genome by genomic engineering. *Proc Natl Acad Sci USA* **106**, 8284-8289.

Oda, H. and Tsukita, S. (2001). Real-time imaging of cell-cell adherens junctions reveals that Drosophila mesoderm invagination begins with two phases of apical constriction of cells. *J Cell Sci* **114**, 493-501.

Strutt, H. and Strutt, D. (2002). Nonautonomous planar polarity patterning in Drosophila: Dishevelled-independent functions of Frizzled. *Dev Cell* **3**, 851-863.

Strutt, H., Gamage, J. and Strutt, D. (2016). Robust asymmetric localization of planar polarity proteins is associated with organization into signalosome-like domains of variable stoichiometry. *Cell Rep* **17**, 2660–2671.

Development • Supplementary information
