# Supplementary material for: QuantifyPolarity, a new tool-kit for measuring planar polarized protein distributions and cell properties in developing tissues
Source: Development. 2021 Sep 7;148(18):dev198952. doi: 10.1242/dev.198952 (PMC8451067; doi:10.1242/dev.198952)
Supplement: Reviewer comments [file develop-148-198952_review_history.pdf]

QuantifyPolarity, a new tool-kit for measuring planar polarized protein distributions and cell properties in developing tissues

Su Ee Tan, Weijie Tan, Katherine H Fisher and David Strutt
DOI: 10.1242/dev.198952

Editor: Thomas Lecuit

Review timeline

Original submission:	24 November 2020
Editorial decision:	20 January 2021
First revision received:	9 April 2021
Editorial decision:	13 May 2021
Second revision received:	17 June 2021
Editorial decision:	12 July 2021
Third revision received:	20 July 2021
Accepted:	26 July 2021

Original submission

First decision letter

MS ID#: DEVELOP/2020/198952

MS TITLE: QuantifyPolarity, a new tool-kit for measuring planar polarized protein distributions and cell properties in developing tissues

AUTHORS: Su Ee Tan, Weijie Tan, Katherine H Fisher, and David Strutt

I apologise for the delay before being able to come back to you. I have now received all the referees reports on the above manuscript, and have reached a decision. The referees' comments are appended below, or you can access them online: please go to BenchPress and click on the 'Manuscripts with Decisions' queue in the Author Area.

The overall evaluation is clearly very positive although one of the reviewers has a number of concerns that in my view need to be addressed carefully. Please attend to all of the reviewers' comments in your revised manuscript and detail them in your point-by-point response. If you do not agree with any of their criticisms or suggestions explain clearly why this is so.

Reviewer 1

Advance summary and potential significance to field

In this paper, Tan et al. propose a novel method to quantify cellular polarization of proteins using Principle Component Analysis. The authors compare their method to two existing methods, Ratio-based and Fourier Series-based.
Specifically, the authors compare these three methods using simulated cells, and data generated from Drosophila pupal wing, pupal notum, wing discs, and embryonic epidermis. Through these various sources of data, the three methods are tested on a variety of cell geometries. They conclude that the PCA method performs better for tissues with highly irregular cell geometries.

© 2021. Published by The Company of Biologists under the terms of the Creative Commons Attribution License (https://creativecommons.org/licenses/by/4.0/).

These three methods and additional analyses are packaged into the QuantifyPolarity software for ease of use.
While there aren’t substantial new biological insights in the paper – it mostly confirms existing findings in terms of Fz and E-Cadherin polarization - the software developed will be extremely useful to the polarity field. It allows the user to compare between the three methods discussed in the paper using a simple Graphical User Interface. This tool will be broadly useful to those interested in quantifying polarity of various proteins, regardless of organism and tissue of interest.

Comments for the author

Major Comments
1. While some disadvantages of the existing Ratio and Fourier Series methods were given, an explanation for why these methods are more sensitive to irregularly-shaped cells would be helpful. Additionally, it is unclear from the text why PCA was chosen as the appropriate solution and why PCA is not sensitive to cell geometry. The explanation for the PCA method is quite short in the main text. The conclusion of the comparisons between the three method is that the user can use the software to pick between the three methods and decide for themselves which method should be used. However, there should be some guidelines recommended or rationale for when a certain method should be used.

2. The description of the PCA method in the methods section would benefit from including some rationale behind the major steps. Are Equations 4 and 5 standard practices or are these novel? Also, why is polarity calculated as stated in Equation 13? Is the polarity magnitude defined as the difference between the standard deviations along the two principal components (with the square root of the eigenvalues of the covariance matrix being the standard deviation in that principal component?)? I think explanations that provide some intuition behind the equations could be useful to people who aren’t familiar with the math behind PCA. Perhaps a schematic would also help?

3. One suggestion to bolster this study and help draw more differences between the three methods would be to include simulated data with some noise parameter. This would more closely simulate real data, as PCP proteins are known to cluster along junctions, and rarely show smooth high and low intensities, as shown in Fig. 1D. This could reveal further differences between the three methods, allowing the user to have a better understanding of which method may best apply to their data.

4. Several figures of this paper include rose plots displaying weighted histograms of calculated polarity as well as the mean angle difference between two methods. However, perhaps due to the weighed histogram, often the mean angle difference reported does not match what is shown on the figure. For example, in Fig. 2E/C” and Fig 3F’’, the reported mean angle differences are quite large however the rose plots appear to be nearly overlapped, in terms of orientation.
The mean angles should be displayed on the rose plot for each method so that the mean angle difference is shown. Additionally, the decision for when the mean angle difference is significantly different appears to be arbitrary. For example, in Fig. 3C the mean angle difference of 14.59° between the Ratio and PCA method is reported as not significantly different. However, in Fig. 2E’/E’’’ the mean angle difference between Fourier Series and PCA of 17.32° is reported as being in less agreement. One solution is to utilize a statistical test, such as Two-sample Kuiper’s Test, to show the if two samples are from the same distribution. This would allow for an unbiased statement of significance to be applied.

5. Based off the results from Fig. S2C-C’, the authors argue that a weighted histogram should be used to display polarity distributions since even proteins that are not polarized, such as E-cadherin in the wing, will have some non-zero average polarity magnitude. The authors should present both the weighted and unweighted histograms for Fig. S2C-C’ to illustrate this point.

Minor comments
1. Figure 2 labels do not match to legend and text.
2. Figure 2D is titled “32 hAPF pupal wing” while all other instances have the time point after the tissue name “Pupal wing 32 hAPF”.

© 2021. Published by The Company of Biologists under the terms of the Creative Commons Attribution License (https://creativecommons.org/licenses/by/4.0/).

3. In Figure 3D, the polarity nematics are difficult to see in order to compare the three methods. Perhaps the colors should be brighter and tensors could be scaled to be bigger?
4. In the main text the authors refer to a study reporting E-Cadherin polarization in the embryonic epidermis (Bulgakova et al, 2013), and then compare the three methods at measuring this asymmetry. How was E-cad polarity measured in that paper and how does it compare to the Ratio and Fourier Series-based methods, which were poor at detecting E-Cad asymmetry in this tissue?
5. While the software appears to be available on Github right now, the folder containing the source code is empty. We strongly encourage that the authors commit to this software being open source. This would give QuantifyPolarity a distinct advantage to existing popular tools.

Reviewer 2

Advance summary and potential significance to field

In the manuscript entitled 'QuantifyPolarity, a new tool-kit for measuring planar polarized protein distributions and cell properties in developing tissues', Tan et al. propose a new method to quantify planar cell polarity in tissues, which is inspired by principal components analysis (PCA). They compare it to two existing methods. The first is based on the ratio of fluorescence intensity on horizontal versus vertical cell edges and the second is based on Fourier coefficients of the angular fluorescence intensity. They compare these three methods by testing a limited number of theoretical scenarios as well as experimental data from the Drosophila pupal wing, the wing disc and the embryonic epidermis. As major result of this manuscript, the authors conclude that the proposed PCA method is independent of cell geometry, while the other two are not. Finally, the authors have built a software to ease the use of the three polarity quantification methods on biological images.

Despite the key role of planar cell polarity in development, it is often not quantitatively studied. Indeed, in many papers, authors still decide
'by eye' whether or not a cell is polarized. Thus, there is a real need in this field for new unbiased methods and for careful comparison with existing methods.
However, we find that in its current form, the manuscript has major issues, which we list and discuss in detail.

Comments for the author

Main problems:
1. Based on its description in the Methods part, the proposed PCA method appears to be just a minor modification of the existing Fourier method.

2. Results in Figure 1 G and H appear to be incompatible with one another. Importantly, this casts serious doubt on the claim that the PCA method is independent of cell geometry in the sense of Fig. 1G. Unfortunately, we are not able to test this further as the authors did not provide us with the code for their polarity calculations.

3. There appear to be problems with the intensity normalization in the proposed PCA method. In particular, it seems that the polarity angle determined with the PCA method depends on global intensity scaling of the image used, and thus on the imaging conditions (brightness).

4. Last, but not least: There is a conceptual issue with the question "Which polarity quantification method is independent of cell geometry?" as the answer to this question depends on how one defines "independent of geometry". This is very important, because, for example, the authors choose to define "independent of geometry" as depicted in Fig. 1G, where they compare cell polarity between some distribution on an unstretched cell and then stretch the cell while keeping the min and max intensity constant, even if this means that then the total amount of polarity proteins will increase. There are other possible choices, e.g. (i) to require that one needs to compare situations where the total amount of polarity protein remains constant, or (ii) to require that the angular distribution remains the same while changing the shape, etc. All of these definitions of shape independence are similarly reasonable but different from one another. Thus,

© 2021. Published by The Company of Biologists under the terms of the Creative Commons Attribution License (https://creativecommons.org/licenses/by/4.0/).

there is no single definition of "independent of geometry", and the answer to "Which polarity quantification method is independent of cell geometry?" is always connected to the definition of shape independence.

While we reiterate that proposing alternatives to existing methods and comparing everything is welcome and can only be helpful for everyone in the field, we are afraid the fundamental issues mentioned above prevent us from recommending publication.

--

Details (on points 1-3):

On point 2: According to the Methods part, both Fourier method and proposed PCA method only depend on the angular intensity distribution I(theta). In the theoretical tests in this manuscript, this distribution is changed in particular in Fig. 1G-H". The following comparison of Fig. 1G and Fig. 1H-H" suggests that both are inconsistent with one another.
In Fig. 1H-H", the peak polarity in I(theta) appears in two angular intervals centered at angles 0 and pi, where only the width of the angular intervals is changed as the number of units of peak protein is changed. In this case, indeed the polarity magnitudes for both methods depend on the angular distribution with a similar typical sensitivity, even though the maxima appear in different places.
We note that the angular intensity distribution I(theta) also changes in Fig. 1G: peak intensity distributes within narrower and narrower intervals centered around angles 0 and pi, which should lead, formally, to the same kind of distributions I(theta) as in Fig. 1H-H", where again only the angular width of peak polarity is changed. It is thus very surprising to us that in Fig. 1G the polarity magnitude with the proposed PCA method changes much less than with the Fourier method despite not doing so in Fig. 1H.

This points to a crucial discrepancy that could unfortunately not be further evaluated as the code was not available.
On point 1 above: The authors seem to have missed that the proposed PCA method as described in the Methods part is just two small modifications away from the existing Fourier method. These two modifications are:
(a) The intensity I(theta) in the Fourier integral is replaced by with Î^2(theta).
(b) The non-normalized Fourier method results in the polarity magnitude |Q| = 2*pi*(lambda1-lambda2) while the PCA methods results has polarity magnitude p = sqrt(lambda1) - sqrt(lambda2).

This can be seen by using the relations cos^2(theta)=(1+cos[2 theta])/2, cos(theta)sin(theta)=sin(2theta)/2 and sin^2(theta)=(1-cos[2 theta])/2 in Eqs. (9-11), respectively. This means that the matrix **sigma** computed in Eq. (12) can be expressed as:
sigma = (**N*1 + Q**)/(4*pi), where N is the normalization factor for the Fourier method, **1** is the 2x2 identity matrix, and **Q** is the non- normalized **Q** matrix in the Fourier method. As a result, the eigen values will be lambda1 = (N+|Q|)/(4*pi) and lambda2 = (N-|Q|)/(4*pi), where |Q| is the magnitude of the single-cell polarity used in the Fourier method, and as a result |Q| = 2*pi*(lambda1-lambda2). Moreover, the angle theta the authors defined in Eq. (14) is the same as the one obtained from the Fourier method as the eigen vectors of **sigma** and **Q** are the same (as they only differ by a factor and the identity matrix).

On point 3: In the modification (a) compared to the Fourier method, there seem to be two closely related issues:
(i) How is the factor k determined?
(ii) The PCA polarity definition, and even the polarity angle depends in principle on the scaling of the overall image intensity. This means that the polarity angle would depend on the imaging conditions (brightness). To show this, let us say we increase the image intensity by some factor c: I --> c*I. The authors have maybe already realized that the PCA polarity angle in real cells changes when they vary k, which is essentially the same effect.
As discussed above, the PCA polarity angle can be computed using the Fourier method from Î^2 = (ln[k*c] + ln I)^2 = (ln [k*c])^2 + 2(ln [k*c])(ln I) + (ln I)^2.
The first term being a constant, only the last two terms matter for determining the angle. Thus, in the PCA method, the authors essentially compute the polarity angle defined by the linear combination of the angular distributions (ln I) and (ln I)^2. The angles of either distribution are in

© 2021. Published by The Company of Biologists under the terms of the Creative Commons Attribution License (https://creativecommons.org/licenses/by/4.0/).

general not the same, and so the angle that results from the PCA method will depend on the relative weighting of these two terms, 2(ln [k*c]), which depends on the overall intensity scaling c. Hence, the polarity angle resulting from the PCA method is expected to depend on imaging conditions such as brightness

Other issues:
5. The theoretical scenarios tested in Fig. 1 are somewhat limited. In particular, the authors test only the effect of two different intensity levels, whereas there are many different intensity levels in real tissues. This is particularly relevant as the biggest difference between the proposed PCA method and the existing Fourier method only lie in the non-linearity applied to the intensity values (point 1a above). Moreover, conceptual arguments for why the PCA method would be independent of cell shape could help beyond comparing examples.

Reviewer 3

Advance summary and potential significance to field

The polarized localization of proteins within the epithelial plane (planar cell polarity) plays an essential role in developing tissue structure and shape. While the visual appearance of such polarity is striking, quantifying protein distribution at the cellular level is complicated by variations in cell geometries. As such, different quantification methods can have differing outputs due to irregularities in cell geometry.

This manuscript presents a PCA-based method for quantifying planer cell polarity and compares this method to 2 other previously used measurements. The authors directly compare these 3 algorithms for simulated data and in several different tissues: the Drosophila pupal wing and wing imaginal discs. I liked how the authors demonstrated the performance of the 3 algorithms for different proteins (e.g. Fz-EGFP, ECadherin-GFP, and Dachsous) and different genotypes (e.g. Fig. S1). Finally the authors presented a graphical user interface (GUI) that they developed for researchers to quantify planer cell polarity. I really appreciated that they did not just include their PCA algorithm, but included the prior 2 algorithms in the software so that users of the GUI can explore for themselves the different performance of each algorithm. The authors provided a thoughtful discussion of the strengths and weaknesses of each algorithm and their appropriateness with different types of data.

Comments for the author

Overall, I found the manuscript to be well written, thoughtful, and envision that this QuantifyPolarity tool will be extremely valuable for the field. I think the manuscript could more-or-less be published as is. However, I do have some minor suggestions and corrections for the authors.

1) I was wondering if the author's simulation could provide insight into how the various algorithms do with different intensity patterns around the perimeter. For example, if a signal is punctate versus continuous?
What if the signal localizes to tricellular vertices or only at bicellular junctions, can the algorithm identify polarity as well as a continuous distribution?

2) p. 9: Here, I thought it would be helpful for the authors to show the correlation between different polarity measurements and eccentricity. Doing this would be a nice way to show that certain algorithms are affected by eccentricity, but others are less dependent. Something like Figure 6, but comparing the different algorithms.

3) p. 9: The lettering on their figure callouts for Figure 2 are incorrect. They have references for 2E, F, and G and I don't see anything beyond D in the figure.

4) Fig. 2C: It would help to separate the blue, green, and magenta bars rather than plotting them on top of each other so that readers can compare them.

© 2021. Published by The Company of Biologists under the terms of the Creative Commons Attribution License (https://creativecommons.org/licenses/by/4.0/).

First revision

Author response to reviewers' comments

Response to the Editor

First of all, we appreciate your time and effort in coordinating the review of our paper. We have carefully revised the manuscript according to the valuable comments and suggestions provided by the reviewers. We believe that this has significantly improved the work. The major improvements over the previous version are summarized as follows:

- We have added schematics to illustrate the principles underlying the three different polarity methods and our definition of planar polarization.
- We have added the cell compression operation in the Material and Methods section for the PCA method (which was omitted by error). Besides that, we have provided a detailed explanation for why both Ratio and Fourier Series methods are sensitive to cell elongation while the PCA method is not in the revised manuscript.
- We have provided more detailed information on the independence of polarity angle readout from the PCA method to image brightness.
- In response to the suggestions of the reviewers, we have added five additional simulations (based on different biological systems and image conditions) to assess the performance of all polarity methods. These simulation results have been added into either the main or supplementary figures.
- We have added a table of summarized comparison between the three polarity methods to assist users in selecting the most suitable method.
- We have shortened the manuscript (to be under 7000 words) and improved the overall presentation of the figures.

In our responses to the reviewers, we have also included a number of additional figures where we thought they would be helpful to explain a point. These are denoted as Figure R1 etc.

Our responses to the specific comments of the reviewers are listed below.

Responses to Reviewer 1

Thank you very much for your time and effort in reviewing the manuscript. We appreciate the valuable comments and suggestions, which have helped us to improve the work. The manuscript has been revised according to the comments provided by you as well as by the other reviewers. Below we detail our responses to your comments, point-by-point, for your convenience.

Comment 1

In this paper, Tan et al. propose a novel method to quantify cellular polarization of proteins using Principle Component Analysis. The authors compare their method to two existing methods, Ratio-based and Fourier Series-based. Specifically, the authors compare these three methods using simulated cells, and data generated from Drosophila pupal wing, pupal notum, wing discs, and embryonic epidermis. Through these various sources of data, the three methods are tested on a variety of cell geometries. They conclude that the PCA method performs better for tissues with highly irregular cell geometries. These

© 2021. Published by The Company of Biologists under the terms of the Creative Commons Attribution License (https://creativecommons.org/licenses/by/4.0/).

three methods and additional analyses are packaged into the QuantifyPolarity software for ease of use.
While there aren't substantial new biological insights in the paper – it mostly confirms existing findings in terms of Fz and E-Cadherin polarization - the software developed will be extremely useful to the polarity field. It allows the user to compare between the three methods discussed in the paper using a simple Graphical User Interface. This tool will be broadly useful to those interested in quantifying polarity of various proteins, regardless of organism and tissue of interest.

Response 1

We would like to thank the Reviewer for these comments.

Comment 2

While some disadvantages of the existing Ratio and Fourier Series methods were given, an explanation for why these methods are more sensitive to irregularly-shaped cells would be helpful. Additionally, it is unclear from the text why PCA was chosen as the appropriate solution and why PCA is not sensitive to cell geometry. The explanation for the PCA method is quite short in the main text. The conclusion of the comparisons between the three method is that the user can use the software to pick between the three methods and decide for themselves which method should be used. However, there should be some guidelines recommended or rationale for when a certain method should be used.

Response 2

Thank you for the suggestions.

- We have provided more explanation in the manuscript on why the Ratio and Fourier Series are more sensitive to elongated cells while the PCA method is not (Line 155-162 Page 7). In brief, our implementation uses a simple cell compression operation to compensate for cell eccentricity, making this method insensitive to varying cell eccentricity.

- We agree that it is helpful to have guidelines for users on which method to choose. Due to the biological variation between different polarization systems, it is not simple to recommend the 'right' method for all systems. Instead, we have provided a table (Table 1) which summarizes the performance comparison between all three polarity methods on different cell geometry, protein distribution and image conditions (such as brightness and signal-to-noise ratios. Hopefully, this will be useful in guiding users to select the method most suited to their system.

Comment 3

The description of the PCA method in the methods section would benefit from including some rationale behind the major steps. Are Equations 4 and 5 standard practices or are these novels? Also, why is polarity calculated as stated in Equation 13? Is the polarity magnitude defined as the difference between the standard deviations along the two principal components (with the square root of the eigenvalues of the covariance matrix being the standard deviation in that principal component?)? I think explanations that provide some intuition behind the equations could be useful to people who aren't familiar with the math behind PCA. Perhaps a schematic would also help?

Response 3

Thank you for the valuable comment.

© 2021. Published by The Company of Biologists under the terms of the Creative Commons Attribution License (https://creativecommons.org/licenses/by/4.0/).

We have updated the manuscript with a schematic (Figure 1D-F) to illustrate the basic working principles of all three polarity methods.

The main distinction of our proposed method from the standard PCA method lies in Eq. 5 and 13 for intensity normalization and the polarity magnitude computation respectively. Details regarding each equation are provided below:

$$w_i = d\theta_i = \frac{1}{2}\left(\theta_{i+1} - \theta_{i-1}\right). \tag{4}$$

- Eq. 4: A common application of angular weighting as shown in this equation is for the summation of a discretized integral, for e.g. in the calculation of moment of inertia. However, such angular weighting is not a standard practice in PCA, for e.g., dimensional reduction in machine learning application. The reason why we used this weighting formulation is to compensate for the non-uniformity of the angular distributions of pixels along the cell junctions.

$$\hat{I}_i = \ln\left(k \cdot I_i\right), \tag{5}$$

- Eq. 5 is novel, to our best knowledge. This equation is essential for the intensity normalization.

$$\hat{x}_i = \hat{I}_i \cos\left(\theta_i\right), \tag{6}$$

$$\hat{y}_i = \hat{I}_i \sin\left(\theta_i\right). \tag{7}$$

- Eq. 6-7 differ from the standard PCA calculation. The standard practice of PCA (for e.g. in physics) uses positional XY-coordinates (both X and Y are the coordinates of pixel of interest) as below:

$$x_i = r_i \cos\ (\theta_i)\,,$$
$$y_i = r_i \sin\ (\theta_i)\,,$$

where r_i is the distance from the centroid of the cell.
However, PCA computation using such positional XY-coordinates resulted in its dependency on cell geometry. Therefore, instead of positional XY-coordinates, we take the intensities as the distances from the centroid at specific angles, which are then converted into cartesian coordinates using trigonometric functions to obtain transformed coordinates as shown in Eq. 6-7.

$$w_\Sigma = \sum_{i=1}^{n} w_i, \tag{8}$$

$$\sigma_{xx} = \frac{1}{w_\Sigma}\sum_{i=1}^{n} w_i \hat{x}_i^2, \tag{9}$$

$$\sigma_{xy} = \sigma_{yx} = \frac{1}{w_\Sigma}\sum_{i=1}^{n} w_i \hat{x}_i \hat{y}_i, \tag{10}$$

$$\sigma_{yy} = \frac{1}{w_\Sigma}\sum_{i=1}^{n} w_i \hat{y}_i^2, \tag{11}$$

$$\boldsymbol{\sigma} = \begin{bmatrix} \sigma_{xx} & \sigma_{xy} \\ \sigma_{yx} & \sigma_{yy} \end{bmatrix}, \tag{12}$$

- Eq. 8-12 are slightly different from the standard PCA calculation, where $\sigma_{xx} = \frac{1}{w_\Sigma}\sum_i w_i \cdot (\hat{x}_i - \bar{x})^2$ (similarly for the calculation of $\sigma_{xy} = \sigma_{yx}$ and σ_{yy}). Instead, we use $\sigma_{xx} = \frac{1}{w_\Sigma}\sum_i w_i \cdot (\hat{x}_i)^2$, where w_i is the weighting, i is the index for each point on the cell boundary, $\hat{x}_i$ is the transformed x-coordinates, and $\bar{x}$ is the average of all $\hat{x}_i$.

© 2021. Published by The Company of Biologists under the terms of the Creative Commons Attribution License (https://creativecommons.org/licenses/by/4.0/).

This modification is necessary in order to accommodate cases such as unequal peak protein intensities (with higher peak protein on one side and significantly lower peak protein on the opposite side) or unipolarity. This is because such a protein distribution would bias the centroidal position $(\bar{x}, \bar{y})$ away from its origin thus affecting the polarity readout.

- $$p = \sqrt{\lambda_1} - \sqrt{\lambda_2}, \qquad (13)$$

  Eq. 13 is novel. The explanation below should provide a basic understanding as to why the polarity magnitude is defined as shown in Eq. 13.
  The eigenvalues $\lambda_{1,2}$ obtained from the covariance matrix $\boldsymbol{\sigma}$ have several interesting properties:

  a. When the protein distribution is homogeneous, $\lambda_1 = \lambda_2$
  b. The greater the bipolarity, the greater the difference between λ_1 and λ_2

  Meanwhile, we adopted the square root of eigenvalues $\sqrt{\lambda_{1,2}}$, which give a more intuitive interpretation as an "average" of $\hat{I}_i$.
  As shown in Eq. 6-7, each pixel on the cell boundary can be represented with the transformed coordinates $(\hat{x}_i, \hat{y}_i)$. By projecting $(\hat{x}_i, \hat{y}_i)$ along the eigenvectors $v_{1,2}$, we obtain their projection as $p_{i;\,1,2}$, where $p_{i;\,\mu}$ should be interpreted as the projection of the i-th pixel on the cell boundary onto the μ-th eigenvector.
  The (weighted) root mean squares (RMS) of these projections are computed as below:

  $$\mathrm{RMS}(p_{i;\,1,2}) = \sqrt{\frac{1}{w_\Sigma}\sum_i w_i \cdot (p_{i;\,1,2})^2}.$$

  This is equivalent to $\sqrt{\lambda_1}$ and $\sqrt{\lambda_2}$ respectively. So, polarity magnitude is defined as the difference of the square root of eigenvalues.

- $$\theta = \frac{1}{2}\tan^{-1}\left(\frac{2\sigma_{xy}}{\sigma_{xx} - \sigma_{yy}}\right). \qquad (14)$$

  Eq. 14 is standard practice in calculating the direction of the principal axis in 2D.

We have also included some of the explanation for these equations in "Quantification of planar polarization using a PCA-based method" in Materials and Methods section section (Lines 569-603).

Comment 4

One suggestion to bolster this study and help draw more differences between the three methods would be to include simulated data with some noise parameter. This would more closely simulate real data, as PCP proteins are known to cluster along junctions, and rarely show smooth high and low intensities, as shown in Fig. 1D. This could reveal further differences between the three methods, allowing the user to have a better understanding of which method may best apply to their data.

Response 4

We appreciate this valuable suggestion.

- We have simulated data with artificially added noise and added it into the manuscript (Figures 2F and S2A and accompanying text and legends). (Lines 186-191)

© 2021. Published by The Company of Biologists under the terms of the Creative Commons Attribution License (https://creativecommons.org/licenses/by/4.0/).

Comment 5(i)

Several figures of this paper include rose plots displaying weighted histograms of calculated polarity as well as the mean angle difference between two methods. However, perhaps due to the weighed histogram, often the mean angle difference reported does not match what is shown on the figure. For example, in Fig. 2E/C" and Fig 3F'', the reported mean angle differences are quite large, however the rose plots appear to be nearly overlapped, in terms of orientation. The mean angles should be displayed on the rose plot for each method so that the mean angle difference is shown.

Response 5(i)

Many thanks for raising this concern. Allow us to clarify the main purpose of this measure. For each individual cell, we would like a way to compare how 'similar' or 'different' the individual polarity angle readout is from each polarity method. The main issue is that mean angle difference measures the difference in polarity angles obtained from different methods on a cell- by-cell level, whereas a weighted rose histogram is a way to visualize overall polarity angle distribution.

- Consider a simplified case below:
  Polarity angle computed from Fourier Series: Cell A = 30° and Cell B = 60°
  Polarity angle computed from PCA: Cell A = 60° and Cell B = 30°

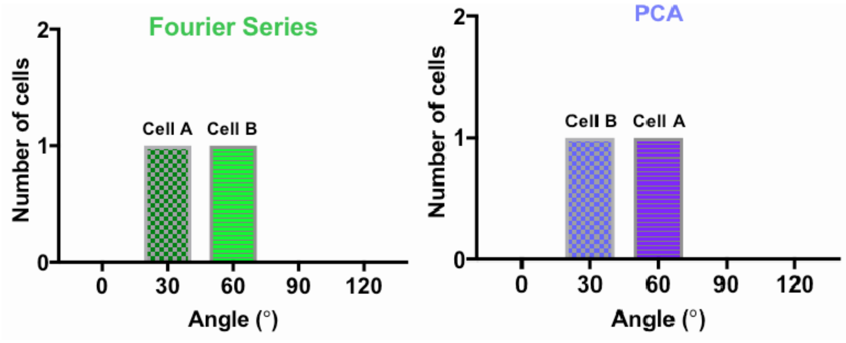

Figure R1: Histogram for the Fourier Series (Left panel) and PCA (Right panel) methods

Both methods will output exactly the same histogram because they have similar angle distribution (Figure R1) i.e. when overlaying the Fourier Series histogram onto PCA histogram, the angle plot will be identical. Thus, from the histogram, we can't tell whether the polarity angles obtained from Fourier Series and PCA methods differ at the cell-by- cell level.

- In contrast, the measure we use (mean angle difference) directly captures the average difference in polarity angles obtained from two different methods for all cells. The mean angle difference between Fourier Series and PCA for cells A and B is calculated as below:

  Angle difference in Cell A = abs(30-60) = 30°

  Angle difference in Cell B = abs(60-30) = 30°

  Mean angle difference = (30+30)/2 = 30°

Overall, both methods can have equal angle distribution but with a mean angle difference of 30°. This explains why some rose plots appeared to be overlapping but still exhibit high mean angle differences.

© 2021. Published by The Company of Biologists under the terms of the Creative Commons Attribution License (https://creativecommons.org/licenses/by/4.0/).

- Regarding displaying the mean polarity angle for each method besides the rose plots: The mean polarity angle does not simply relate to the measure of mean angle difference.
  Following from the example above, the mean polarity angle (for cell A and B) computed from the Fourier Series and PCA methods would be exactly the same, which is 45°. This is because both methods give the same angle distribution. Hence, displaying the mean polarity angle next to the mean angle difference would potentially confuse the reader.

- We understand that the mean angle difference described in methods section may not be sufficiently clear which might have led to some misunderstanding. Hence, we modified the Materials and Methods section for the mean angle difference to try to explain more clearly (Lines 656-670).

Comment 5(ii)

Additionally, the decision for when the mean angle difference is significantly different appears to be arbitrary. For example, in Fig. 3C the mean angle difference of 14.59° between the Ratio and PCA method is reported as not significantly different. However, in Fig. 2E'/E''' the mean angle difference between Fourier Series and PCA of 17.32° is reported as being in less agreement. One solution is to utilize a statistical test, such as Two-sample Kuiper's Test, to show the if two samples are from the same distribution. This would allow for an unbiased statement of significance to be applied.

Response 5(ii)

Thank you for this suggestion and comment.

- Indeed, the suggested statistical tests such as Kuiper (also Watson) compare between two samples distribution, considering the circularity properties of the data. However, as shown in Response 5(i) above, both methods could have similar angle distribution despite there being angle differences at the cell-by-cell level comparison (Figure R1). Hence, we resorted to a simpler method of measuring angle differences for each cell output from two different methods. We acknowledge that with this method it is difficult to address what might be considered a significant angle difference and vice versa.
  However, it seems reasonable to assume that the smaller the angle differences, the more in agreement both methods are in terms of polarity angle. We have modified the manuscript as suggested to make this clearer (Lines 268-270) and (Lines 287-290).

Comment 6

Based off the results from Fig. S2C-C', the authors argue that a weighted histogram should be used to display polarity distributions since even proteins that are not polarized, such as E- cadherin in the wing, will have some non-zero average polarity magnitude. The authors should present both the weighted and unweighted histograms for Fig. S2C-C' to illustrate this point.

Response 5(ii)

We thank the reviewer for this helpful suggestion.

- We have included unweighted histograms for polarity angles obtained from three different methods in Figure S3. As expected, the unweighted histograms displayed more dispersed polarity angles as compared to weighted histograms (compare Figure S3B-B'' and C-C'') (Lines 256-263).

© 2021. Published by The Company of Biologists under the terms of the Creative Commons Attribution License (https://creativecommons.org/licenses/by/4.0/).

Responses to minor comments

1. Figure 2 labels do not match to legend and text.

   Apologies. We have now corrected this error in the revised manuscript.

2. Figure 2D is titled "32 hAPF pupal wing" while all other instances have the time point after the tissue name "Pupal wing 32 hAPF".

   Thank you for your careful observation. We have changed the title in Figure 3D (old Figure 2D) to "Pupal wing 32 hAPF" to be consistent

3. In the main text the authors refer to a study reporting E-Cadherin polarization in the embryonic epidermis (Bulgakova et al, 2013), and then compare the three methods at measuring this asymmetry. How was E-cad polarity measured in that paper and how does it compare to the Ratio and Fourier Series-based methods, which were poor at detecting E-Cad asymmetry in this tissue?

   In (Bulgakova et al., 2013) they manually classified all junctions into vertical and horizontal junctions and then computed the average junctional intensity for both junctions. They reported that there is a statistically higher amount of E-Cadherin intensity on the horizontal as compared to the vertical junctions in wild-type *Drosophila* embryo, suggesting that angle of cell polarity is oriented at approximately 90° degree, which is consistent with the results from the PCA method.

   One explanation as to why both the Ratio and Fourier Series methods were poor at detecting polarity angle of E-Cad could be attributed to the influence of junctional proteins in abutting cells. As shown in Figure R2, there are some junctional proteins from neighboring cells B and C (as indicated by pink circles) localized adjacent to or on the tricellular junctions on the long junctions (yellow arrows) of cell A. These populations of neighboring protein could contribute significantly to the angular protein distribution of the cell A as they are located closer to the centroid of cell A as compared to the proteins on both short junctions (cyan arrowheads). Note that, by taking protein angular distribution, junctional protein populations (from neighboring cells along the longer junctions) that are closer to the cell centroid exhibit higher 'weight' as compared to proteins that are further away from the cell centroid (on both shorter junctions). Since both the Ratio and Fourier Series methods are only dependent on the protein angular distribution, this explains why polarity output from the Ratio and Fourier Series methods give inconsistent readouts. The PCA method, on the other hand, compresses the cell into a regular shape and thereby is less sensitive to the contribution of neighboring protein.

   In our simulations with varying cell elongation (Figure 2C-C' and D-D'), polarity angles obtained from both the Ratio and Fourier Series methods seem to be unaffected by varying degree of cell elongation. This is because in these simulations (and all other simulations), we consider only a single cell, without any neighboring cells. However, from biological dataset, we observed that proteins from neighboring cells can affect the polarity readout obtained from both the Ratio and Fourier Series methods for elongated cells.

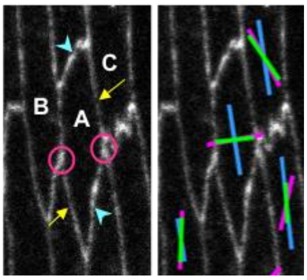

Figure R2: Explanation for the sensitivity of the Ratio and Fourier Series methods to neighboring junctional proteins. Left: Localization of neighboring proteins from cells B and C (pink circles) along the long junctions (yellow arrows) of cell A Right: Quantified cell-scale polarity of E-Cadherin. The magenta, green and blue bars represent the magnitude and orientation of planar polarity for a given cell obtained from Ratio, Fourier Series and PCA methods respectively

© 2021. Published by The Company of Biologists under the terms of the Creative Commons Attribution License (https://creativecommons.org/licenses/by/4.0/).

4. While the software appears to be available on Github right now, the folder containing the source code is empty. We strongly encourage that the authors commit to this software being open source. This would give QuantifyPolarity a distinct advantage to existing popular tools.

We thank you for the comment. This software is intended to be open source which would allow users to modify/enhance it according to their own requirements. All source code will be publicly available in Github once published.

Responses to Reviewer 2

Thank you very much for your time spent reviewing our paper. We highly appreciate your valuable comments and suggestions. The manuscript has now been revised according to the comments provided by you as well as by the other reviewers. Our replies to your comments are detailed below, point-by-point for your convenience.

Comment 1

In the manuscript entitled ‘QuantifyPolarity, a new tool-kit for measuring planar polarized protein distributions and cell properties in developing tissues’, Tan et al. propose a new method to quantify planar cell polarity in tissues, which is inspired by principal components analysis (PCA). They compare it to two existing methods. The first is based on the ratio of fluorescence intensity on horizontal versus vertical cell edges and the second is based on Fourier coefficients of the angular fluorescence intensity. They compare these three methods by testing a limited number of theoretical scenarios as well as experimental data from the Drosophila pupal wing, the wing disc and the embryonic epidermis. As major result of this manuscript, the authors conclude that the proposed PCA method is independent of cell geometry, while the other two are not. Finally, the authors have built a software to ease the use of the three polarity quantification methods on biological images.
Despite the key role of planar cell polarity in development, it is often not quantitatively studied. Indeed, in many papers, authors still decide 'by eye' whether or not a cell is polarized. Thus, there is a real need in this field for new unbiased methods and for careful comparison with existing methods. However, we find that in its current form, the manuscript has major issues, which we list and discuss in detail.

Response 1

Thank you very much for your comments and suggestions. We have tried our best to discuss the issues raised in the following responses.

Comment 2

Based on its description in the Methods part, the proposed PCA method appears to be just a minor modification of the existing Fourier method
The authors seem to have missed that the proposed PCA method as described in the Methods part is just two small modifications away from the existing Fourier method. These two modifications are:
(a) The intensity I(theta) in the Fourier integral is replaced by with Î^2(theta).
(b) The non-normalized Fourier method results in the polarity magnitude |Q| = 2*pi*(lambda1- lambda2) while the PCA methods results has polarity magnitude p = sqrt(lambda1) - sqrt(lambda2).

This can be seen by using the relations cos^2(theta)=(1+cos[2 theta])/2, cos(theta)sin(theta)=sin(2theta)/2 and sin^2(theta)=(1-cos[2 theta])/2 in Eqs. (9-11), respectively. This means that the matrix **sigma** computed in Eq. (12) can be expressed as: **sigma** = (N*__1__ + **Q**)/(4*pi), where N is the normalization factor for the Fourier method, **1** is the 2x2 identity matrix, and **Q** is the non- normalized **Q** matrix in the Fourier method. As a result, the eigen values will be lambda1 = (N+|Q|)/(4*pi) and lambda2 = (N-|Q|)/(4*pi), where |Q| is

© 2021. Published by The Company of Biologists under the terms of the Creative Commons Attribution License (https://creativecommons.org/licenses/by/4.0/).

the magnitude of the single- cell polarity used in the Fourier method, and as a result |Q| = 2*pi*(lambda1-lambda2). Moreover, the angle theta the authors defined in Eq. (14) is the same as the one obtained from the Fourier method as the eigen vectors of **sigma** and **Q** are the same (as they only differ by a factor and the identity matrix).

Response 2

Thank you for the comment.

- Indeed, PCA and Fourier Series methods are mathematically related and are convertible to each other. Nevertheless, they are not a subset of each other (unlike Fourier transform which is a subset of Laplace transform). As explained below, we chose PCA as the basis for our new method due to its specific properties that we think make it a useful alternative to Fourier in this application.

  By way of background, it is our view that due to the properties of the existing Fourier Series method (and also a standard PCA method), there is a need for a better tool that in particular is insensitive to cell elongation and exhibits a symmetrical polarity strength curve. Therefore, we developed three novel formulations in our proposed PCA method to tackle these issues (see details in Response 2 to Reviewer 1), which is evident from the performance of our method in the simulations. We believe that these practical applications illustrate why the PCA method as implemented provides a useful alternative to the existing Fourier Series method for the biology community.

  In more detail, undesirable features with the existing Fourier Series method are:
  • First, it is highly sensitive to cell elongation (Figure 2C). In contrast to the existing Fourier Series method, our proposed PCA method is insensitive to varying cell elongation
  • Second, the Fourier Series method exhibits an asymmetrical polarity strength curve which could give a deceptive polarity strength interpretation (Figure 2E and R3A). Consider the puncta simulation results below as an example (Figure R3A-A', see details in Response 2 to Reviewer 3) - as it is 'closer' to a real biological system (although the simulation results from both continuous and punctate protein distributions are similar): Due to the asymmetrical nature of the polarization strength curve obtained from the existing Fourier Series method, a simulated cell with $N_{puncta} = 2$ is equally polarized as $N_{puncta} = 18$, even though there is obviously much less protein on the opposite cell junctions for $N_{puncta} = 2$ than $N_{puncta} = 18$ (Figure R3A-A'). Differently from the Fourier Series method, our modified PCA method exhibits a symmetrical polarization strength profile, thus a simulated cell with $N_{puncta} = 2$ is equally as weakly polarized as $N_{puncta} = 22$. We argue that this behavior is closer to what most biologists would expect (but note that our GUI offers them a choice, if they prefer the behavior of the Fourier method in their application).

© 2021. Published by The Company of Biologists under the terms of the Creative Commons Attribution License (https://creativecommons.org/licenses/by/4.0/).

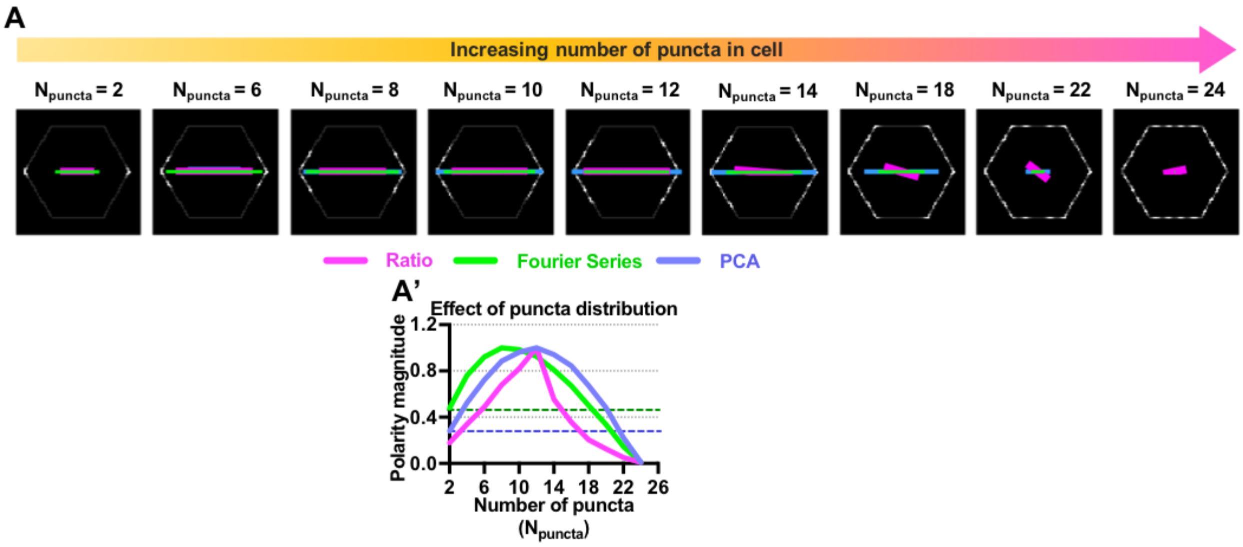

Figure R3: The effect of varying of puncta protein distribution for elongated cells on different polarity methods

(A) Simulated cells with increasing number of puncta (N_{puncta}) while cell geometry and puncta intensity are conserved. The total number of junctional puncta proteins increase gradually (from 2 to starting from both vertical cell poles. For e.g. N_{puncta} = 12 indicates there are a total of 12 puncta in the simulated cell, with 6 puncta equally distributed starting from both poles of vertical junctions. (A') Graphs show quantified normalized polarity magnitudes of cells with varying junctional puncta distribution. Dotted lines indicate unit of junctional peak puncta which exhibit similar polarity magnitude with N_{puncta} = 2 (green - Fourier Series and blue - PCA).

Comment 3

There appear to be problems with the intensity normalization in the proposed PCA method. In particular, it seems that the polarity *angle* determined with the PCA method depends on global intensity scaling of the image used, and thus on the imaging conditions (brightness). According to the Methods part, both Fourier method and proposed PCA method *only depend on the angular intensity distribution* I(theta). In the theoretical tests in this manuscript, this distribution is changed in particular in Fig. 1G-H". The following comparison of Fig. 1G and Fig. 1H-H" suggests that both are inconsistent with one another.
In Fig. 1H-H", the peak polarity in I(theta) appears in two angular intervals centered at angles 0 and pi, where only the width of the angular intervals is changed as the number of units of peak protein is changed. In this case, indeed the polarity magnitudes for both methods depend on the angular distribution *with a similar typical sensitivity*, even though the maxima appear in different places.
We note that the angular intensity distribution I(theta) also changes in Fig. 1G: peak intensity distributes within narrower and narrower intervals centered around angles 0 and pi, which should lead, formally, *to the same kind of distributions* I(theta) as in Fig. 1H-H", where again only the angular width of peak polarity is changed. It is thus very surprising to us that in Fig. 1G the polarity magnitude with the proposed PCA method changes much less than with the Fourier method despite not doing so in Fig. 1H.
This points to a crucial discrepancy that could unfortunately not be further evaluated as the code was not available.

Response 3

Many thanks for the valuable comments.

- The sensitivity of PCA polarity angle to image brightness is addressed in detail in Response 4.

© 2021. Published by The Company of Biologists under the terms of the Creative Commons Attribution License (https://creativecommons.org/licenses/by/4.0/).

- Regarding the PCA method being only dependent on angular protein distribution

  We apologize for the confusion caused. Indeed, in Figure 2C (old Figure 1G) the protein angular distribution changes as the cell elongates (to maintain the amount of protein on both vertical junctions). As the polarity readout from both the Fourier Series and Ratio methods depends solely on the protein angular distribution, this explains why they are sensitive to different cell eccentricities. By depending merely on the protein angular distribution, one disregards the importance of cell geometry itself in the quantification of polarization. However, in contrast to the existing methods, the proposed PCA method takes both the cell geometry and protein angular distribution into consideration, which makes it insensitive to variation in cell geometry. Specifically, a simple cell compression operation allows this method to be independent of the effect of cell eccentricity (Figure 1F). This explains why the PCA readouts in Figure 2C and 2E (old Figure 1H) are different.

  We have revised the Materials and Methods section for the PCA method in the manuscript to explain this (Lines 570-575). Additionally, we have added a schematic to better illustrate all three polarity methods in Figure 1D-F.

- To further illustrate the utility of our PCA-based method, in addition to the *Drosophila* epidermal embryo and pupal wing polarization systems, we also assessed how different polarity methods would behave on a different polarization system, which is the murine epidermal skin polarization system where core polarity proteins localize to the long junctions of elongated cells (Aw et al., 2016). We simulated polarization along the short axis (protein localized to horizontal junctions) of cells with varying eccentricity (Figure 2D-D’). Polarity magnitude computed using the PCA method is independent of varying degrees of cell eccentricity – in the sense that both regular and elongated cells are equally polarized, while polarity magnitudes obtained from both the Ratio and Fourier Series methods are sensitive to varying cell eccentricities (Figure 2C-D). Hence, the PCA method is useful and applicable to these polarization systems.

Comment 4

While we reiterate that proposing alternatives to existing methods and comparing everything is welcome and can only be helpful for everyone in the field, we are afraid the fundamental issues mentioned above prevent us from recommending publication.
In the modification (a) compared to the Fourier method, there seem to be two closely related issues:
A. How is the factor k determined?
B. The PCA polarity definition, and even the polarity angle depends in principle on the scaling of the overall image intensity. This means that the polarity *angle* would depend on the imaging conditions (brightness). To show this, let us say we increase the image intensity by some factor c: I --> c*I. The authors have maybe already realized that the PCA polarity angle in real cells changes when they vary k, which is essentially the same effect.
As discussed above, the PCA polarity angle can be computed using the Fourier method from
$\hat{I}^2 = (\ln[k*c] + \ln I)^2 = (\ln [k*c])^2 + 2(\ln [k*c])(\ln I) + (\ln I)^2$.
The first term being a constant, only the last two terms matter for determining the angle. Thus, in the PCA method, the authors essentially compute the polarity angle defined by the linear combination of the angular distributions (ln I) and (ln I)^2. The angles of either distribution are in general not the same, and so the angle that results from the PCA method will depend on the relative weighting of these two terms, 2(ln [k*c]), which depends on the overall intensity scaling c. Hence, the polarity angle resulting from the PCA method is expected to depend on imaging conditions such as brightness

© 2021. Published by The Company of Biologists under the terms of the Creative Commons Attribution License (https://creativecommons.org/licenses/by/4.0/).

Response 4

We appreciate the valuable comment and the opportunity to explain in more depth.

- Regarding factor k

Factor k is determined empirically via computer simulation. The main reason for introducing factor k is to improve the performance of the PCA method under conditions of extreme low intensities. Below, we provide a simulation to evaluate the performance of the PCA method on varying total cell intensity (extremely low vs high intensity) using different k values (Figure R4A). Note that the relative peak-to-base intensity remains constant at 5-fold. As total protein intensity in a cell is also dependent on simulated cell size, we also plotted polarity magnitude against base intensity instead so that it can be used as a reference for the user. The minimum base intensity value in this simulation is 10 a.u. (Note that even in laser off conditions, image intensity would normally never fall to zero). Since the relative peak-to-base intensities are kept constant for all simulations, ideally polarity magnitude readout should remain constant. However, we observed that polarity magnitude decreases at low base intensity values with decreasing k values (Figure R4A).

With factor k = 1, we found that when base intensity goes below 400 a.u., polarity magnitude gradually decreases (maximum percentage of relative error is 6.12%). In contrast, using factor $k = 10^3$, the maximum relative error is significantly reduced to 0.9% (for base intensity of 10 a.u). When k value is further increased to $5×10^3$ and 10^5 respectively, the relative errors in polarity magnitude further decreased slightly to 0.7% and 0.5% respectively (Figure R4A). It is worth mentioning that the relative errors for factor $k \geq 10^3$ fall within an acceptable range and are in fact are much smaller than the relative errors for all methods due to varying levels of image SNR. Besides that, in real microscopy images, it is not common to have image intensity values range between 0 to 50 a.u.

In short, the purpose of introducing factor k (we chosed $k = 10^3$) is to improve the performance (polarity magnitude) of the PCA method on images with extremely low intensities.

As we explained in the next point, it is also worth mentioning that the polarity angle obtained from the PCA method with different values of k is not affected by varying levels of base intensity (Figure R4A').

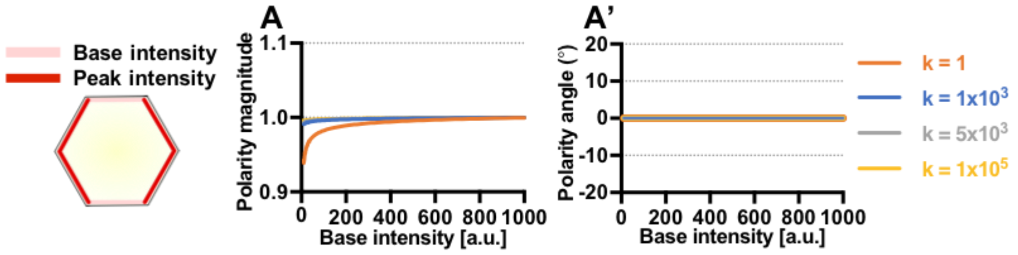

Figure R4: Simulation case to study the effect of varying levels of base intensity on the PCA method with different k values
(A-A') Effects of varying levels of base intensity on different k values with conserved cell geometries, junctional protein distribution and relative peak-to-base intensity. Graphs show quantified polarity magnitudes (A) and polarity angles (A') obtained from the PCA method.

To illustrate the point, we have replotted Figure S1A to consider cases with low total protein intensities (and added panel S1A' for base intensities) and added a paragraph in the "Quantification of planar polarization using a PCA-based method" under Materials and

© 2021. Published by The Company of Biologists under the terms of the Creative Commons Attribution License (https://creativecommons.org/licenses/by/4.0/).

Methods section (Lines 580-585).

- Regarding the independence of polarity angle readout from the PCA method to image brightness

  We are unsure why the reviewer suggests that different distributions ln I_i and (ln I_i)^2 might result in different polarity angles. Possibly the relationship between the two distributions has been overlooked. As demonstrated in Figure S1A'', the polarity angle output from the PCA method is shown to be independent of global intensity scaling/brightness. The complete mathematical proof is as follows:

  Here, instead of I, we denoted the intensities as I_i (so as not to mistaken I as a single scalar value).
  It is true that we are dealing with a linear combination with two distributions, namely ln I_i and (ln I_i)^2, in addition to a uniform distribution (ln kc)^2. As demonstrated by the reviewer, the distribution ln I_i has an additional multiplier of 2 ln kc. While these two distributions are different, the suggestion that the positions of the peaks and therefore the polarity angle are different is incorrect.

  For example, take a closer look into operators (x) = ln x and (x) = x^2 that perform their operation element-wise on a 'vector' quantity. The first distribution ln I_i can be written as (I_i) while the second distribution (ln I_i)^2 can be expressed as $gf(I_i)$

  Intensity values I_i are extracted from standard images, $I_i \in \mathbb{N}^0$. However, as the logarithmic function is not defined when $I_i = 0$, thus the intensity value is set to 1 if it is 0, hence $I_i \in \mathbb{N}^+$. This adjustment should not affect the overall result as even for the lowest bit-depth standard image format (e.g. an 8-bit image) 1/255 ≈ 0.4%. Besides that, there will always be stochastic fluctuation in noise (with intensity >1a.u.) due to the nature of microscope imaging.

  The function f maps positive integers to positive real numbers inclusive of 0:

  $$f = \ln : \mathbb{N}^+ \to \mathbb{R}_{\geq 0}.$$

  On the other hand, the function g maps positive real numbers inclusive of 0 onto itself:

  $$g : \mathbb{R}_{\geq 0} \to \mathbb{R}_{\geq 0}.$$

  It is worth noting that for both functions f, g with the domains defined above, both functions are strict monotonically increasing. This means that if $a > b \Leftrightarrow (a) > (b) \Leftrightarrow (a) > g(b)$

  Hence, although the exact distributions of I_i, ln I_i and (ln I_i)^2 are different, the local and global minima and maxima of all the three distributions are the same. In conclusion, while ln I_i and (ln I_i)^2 are different, the polarity angles determined by their linear combination is independent of intensity scaling.

Comment 5

Last, but not least: There is a conceptual issue with the question "Which polarity quantification method is independent of cell geometry?" as the answer to this question depends on how one defines "independent of geometry". This is very important, because, for example, the authors choose to define "independent of geometry" as depicted in Fig. 1G, where they compare cell polarity between some distribution on an unstretched cell and then stretch the cell while keeping the min and max intensity constant, *even if this means that then the total amount of polarity proteins will increase*. There are other possible choices, e.g. In (i) to require that one needs to compare situations where the total amount of polarity protein remains constant, or (ii) to require that the angular distribution remains the same

© 2021. Published by The Company of Biologists under the terms of the Creative Commons Attribution License (https://creativecommons.org/licenses/by/4.0/).

while changing the shape, etc. All these definitions of shape independence are similarly reasonable but different from one another. Thus, there is no single definition of "independent of geometry", and the answer to "Which polarity quantification method is independent of cell geometry?" is always connected to the definition of shape independence.

Response 5

Many thanks for your comment and suggestions.

- Indeed, we agree with the reviewer that there is no one correct answer to the independence of cell geometry, as it is very much dependent on the specific polarization system being studied. We have also come to this realization in our own internal discussions. We have tried to tackle a wide range of simulated scenarios (trying to be as generalizable as possible) resembling different polarization systems (different proteins and species), to assess how each method performs under different conditions (albeit most of these simulations are designed based on the polarization systems that we are familiar with). As there is no ‘one size fits for all’, this is precisely why we have included all the three methods such that user may choose whichever method that fits their polarization system. We argue that this practical and pragmatic approach is the most helpful. Hopefully, our work will initiate and drive further discussion with explicit simulation of biologically plausible scenarios for each quantification methods, rather than relying solely on theoretical derivation.

- Specifically regarding scenarios where the total amount of protein remains constant: We have performed an additional simulation where we varied cell apical area while conserving the total amount of proteins (the ratio of peak to base intensity is maintained) (Figure R5A-A’’). From our results below, neither the polarity magnitude nor the angle is affected for both Fourier Series and PCA methods. On the other hand, there is slight fluctuation in polarity magnitude (±7%) and angle (±0.5°) obtained from the Ratio method. It is worth noting that the polarity angle readout from the PCA method is not affected by various levels of peak and base intensities (related to Response 4).

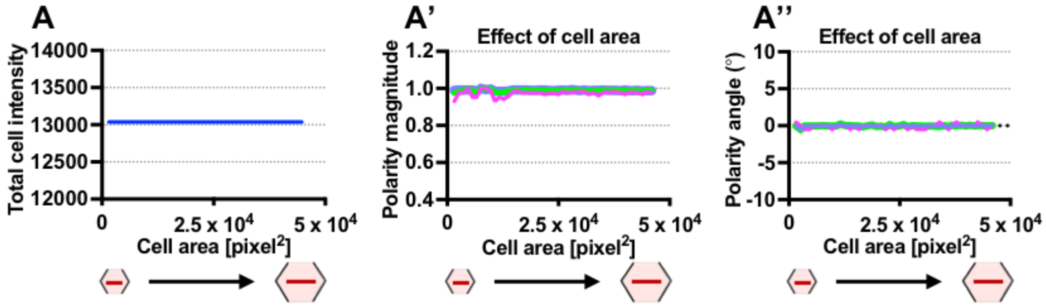

Figure R5: Simulation case to study the effect of varying cell apical area while conserving total amount of protein
(A-A”) Effects of varying cell apical area with conserved cell shape and elongation, junctional protein distribution and relative of peak-to-base intensity. Graphs show quantified polarity magnitudes (A’) and polarity angles (A”) obtained from varying the amount of proteins on cell junctions.

- Regarding simulations where protein angular distribution remains constant: These have been simulated as demonstrated in Figure 2A-B’ of the manuscript. In these simulations, we maintained the protein angular distribution on the cell junctions while varied the cell apical area and shape regularity. Along the same lines as these simulations, we have also performed additional simulations where we conserved the angular distribution of puncta on the cell junctions while varying cell shape and size (Figure 2G-H’). Evidently, polarity readouts obtained from all the polarity methods are robust to different cell sizes and shapes with constant angular distribution – for both continuous or punctate signals.

© 2021. Published by The Company of Biologists under the terms of the Creative Commons Attribution License (https://creativecommons.org/licenses/by/4.0/).

Additionally, we further simulated cells with varying eccentricity while conserving the angular protein distribution on either vertical or horizontal junctions. Simulation results indicate that polarity magnitudes from both the Ratio and Fourier Series methods are unaffected by cell elongation as long as angular protein distribution remained constant for both polarization systems (Figure R6C-D). On the contrary, the polarity magnitude obtained from the PCA method gradually decreases as cells elongate with more protein covering both the vertical and part of horizontal junctions (Figure R6C). Likewise, the PCA's polarity magnitude decreases as there is overall less protein on the horizontal junctions of an elongated cell (Figure R6D). Polarity angles obtained from all methods are not affected by varying cell eccentricities for both cases (Figure 2C'-D', R6C'-D'). However, we don't think that simulations where we conserve the protein angular distribution are very biologically applicable. Below we provided a detailed explanation of why we think this:

Planar polarization of core planar polarity proteins such as Flamingo: Core polarity proteins such as Flamingo accumulate on both vertical junctions of a regular non-elongated cell in fly pupal wings, resulting in the correct positioning of the actin-rich wing hair at the single vertex on the edge of vertical junction (Figure R6A). In the case where we conserved the amount of proteins on specific junctions, a control cell is stretched/elongated with Flamingo localizing only on the vertical junctions (Figure R6A'). We expect this to result in the formation of wing hair at the single vertex on the edge of the vertical junction as well. However, for a case where Flamingo accumulates on both the vertical and part of the horizontal junctions (conserved protein angular distribution) of an elongated cell, this might impair the positioning of the wing hair as there are now three vertices with high protein distribution (Figure R6A'').

Planar polarization of Myosin II: Non-muscle Myosin II is known to exhibit planar polarized localization on specific junctions that drives junctional shrinkage via exertion of contractile force in the gastrulating fly embryo, thereby triggering neighbor exchange or cell intercalation (Bertet et al., 2004; Zallen and Wieschaus, 2004; Simoes Sde et al., 2010). It has been shown that high variance of junctional Myosin levels between neighboring junctions is crucial to drive efficient and oriented neighbor exchange as compared to non-polarized Myosin levels (Curran et al., 2017). Hence, Myosin accumulating on the entire horizontal junctions of an elongated cell (conserved amount of proteins on specific junctions) would result in proper neighbor exchange (Figure R6B'). On the contrary, in the case where Myosin only accumulates on a small part of the horizontal junctions (due to conserved protein angular distribution) could impair the neighbor exchange process (Figure R6A and A''). Hence, high spatial variance and precise localization of Myosin in the former case results in efficient neighbor exchange process, while low spatial variation of Myosin localization in the latter case could impair the process.

Hence, these biological systems emphasize the importance of controlling the amount of proteins on specific junctions to allow proper function rather than necessarily conserving the protein angular distribution for elongated cells. This also explains why we maintained the amount of proteins on vertical junctions while elongating a cell (Figure 2C-D').

© 2021. Published by The Company of Biologists under the terms of the Creative Commons Attribution License (https://creativecommons.org/licenses/by/4.0/).

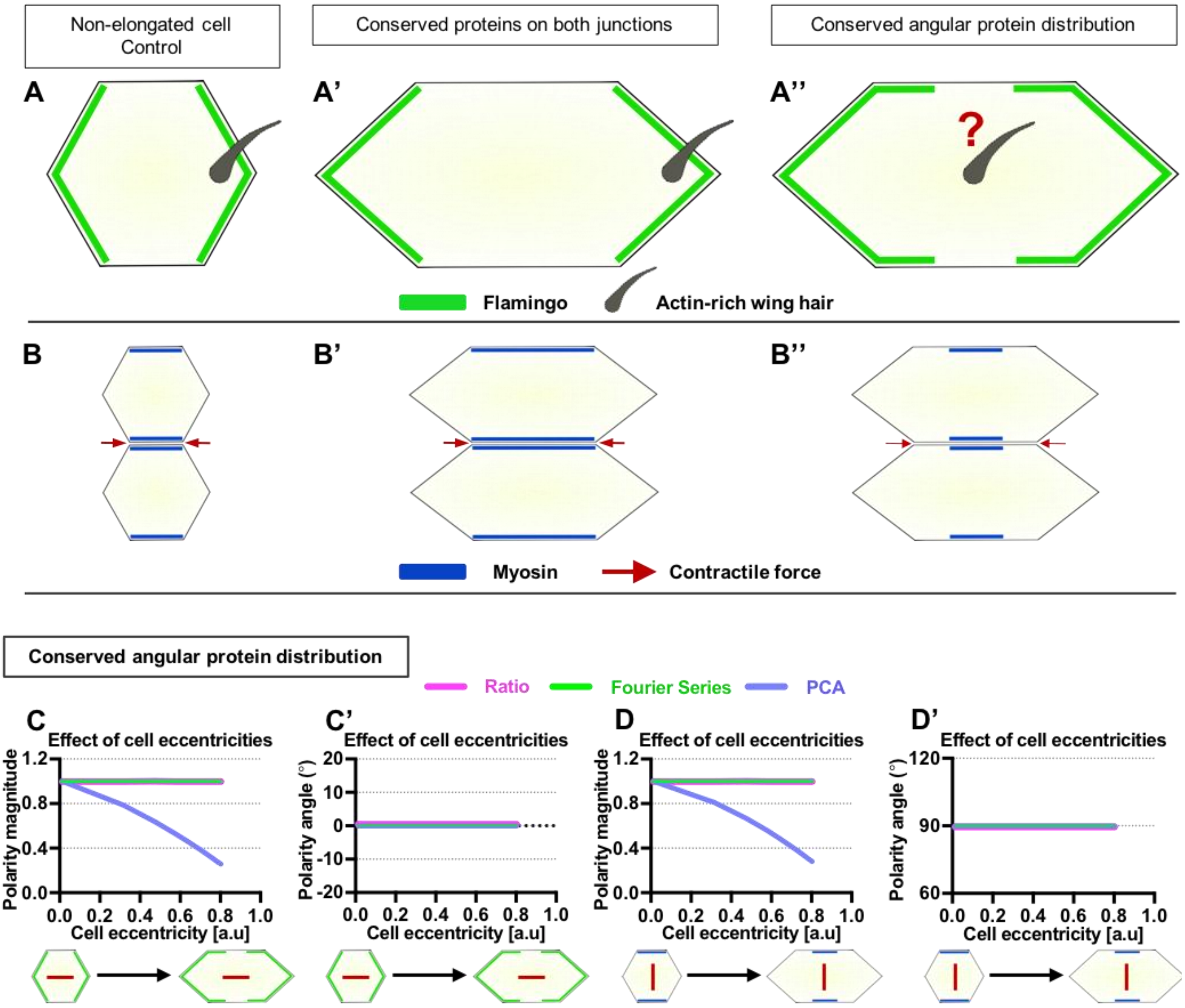

Figure R6: Illustration of two different cases of protein distribution for elongated cells on two different polarization systems

(A-A")Control is a non-elongated cell with Flamingo distributed on both vertical junctions. When the control cell is stretched/elongated, there are two possible scenarios of distributing Flamingo: (A') Preserving the amount of Flamingo on both vertical junctions and (A'') Conserving the Flamingo angular distribution in the elongated cell to be similar to the control cell.

(B-B")Control is a non-elongated cell with Myosin distributed on both horizontal junctions. When the control cell is stretched/elongated, there are two possible scenarios of distributing Myosin: (B') Preserving the amount of Myosin on both horizontal junctions and (B'') conserving the Myosin angular distribution in the elongated cell (similar to the control cell).

(C-C') Protein angular distribution on vertical junctions is conserved in the cell while elongating a cell. Quantified polarity magnitudes (C) and angles (C') of cells with varying eccentricity, from 0 to 0.9.

(D-D') Protein angular distribution on horizontal junctions is conserved in the cell while elongating a cell. Quantified polarity magnitudes (D) and angles (D') of cells with varying eccentricity, from 0 to 0.9.

Comment 6

The theoretical scenarios tested in Fig. 1 are somewhat limited. In particular, the authors test only the effect of two different intensity levels, whereas there are many different

© 2021. Published by The Company of Biologists under the terms of the Creative Commons Attribution License (https://creativecommons.org/licenses/by/4.0/).

intensity levels in real tissues. This is particularly relevant as the biggest difference between the proposed PCA method and the existing Fourier method only lie in the non-linearity applied to the intensity values (point 1a above). Moreover, conceptual arguments for why the PCA method would be independent of cell shape could help beyond comparing examples.

Response 6

We thank the reviewer for the suggestions.

- We understand the point regarding possible shortcomings of our simulations and thus, we have added additional simulations such as the effect of multiple intensity levels (puncta-like) to mimic actual protein distributions more realistically (as suggested by Reviewer 3). With puncta-like distributions, we have found that polarity output from the Fourier Series and PCA methods are least affected by varying cell area, regularity and puncta distribution (Figure 2G-G'', F-F' and S2B-B''). Additionally, we also examined the effect of varying noise levels and tricellular localization on these methods (as suggested by Reviewer 1 and 3) (Figure 2F-F' and S2C-C'').
- While there are potentially endless scenarios we could simulate (for example, varying puncta size, spacing, base-to-peak profile [linear, logistic, …], puncta profile [Gaussian, square, …], and so on), these basic simulations serve the purpose of providing a baseline comparison between different polarity quantification methods and a general understanding of how each individual method behaves under these scenarios. To our best knowledge, this has never been studied, compared and validated in detail in any publications. Hopefully, this will motivate further discussion and comparison of different scenarios in future publications.
- As explained in Response 3, the PCA method uses a simple cell compression operation to compensate for cell eccentricity, making this method insensitive to varying cell eccentricity.

Responses to Reviewer 3

Thank you very much for your time and effort necessary to review the manuscript. We sincerely appreciate all the valuable comments and suggestions, which have helped us to improve the quality of the article. The manuscript has been revised according to the comments provided by you as well as by the other reviewers. Below we detail our responses to your comments, point-by-point, for your convenience.

Comment 1

The polarized localization of proteins within the epithelial plane (planar cell polarity) plays an essential role in developing tissue structure and shape. While the visual appearance of such polarity is striking, quantifying protein distribution at the cellular level is complicated by variations in cell geometries. As such, different quantification methods can have differing outputs due to irregularities in cell geometry.
This manuscript presents a PCA-based method for quantifying planer cell polarity and compares this method to 2 other previously used measurements. The authors directly compare these 3 algorithms for simulated data and in several different tissues: the *Drosophila* pupal wing and wing imaginal discs. I liked how the authors demonstrated the performance of the 3 algorithms for different proteins (e.g. Fz-EGFP, ECadherin-GFP, and Dachsous) and different genotypes (e.g. Fig. S1). Finally, the authors presented a graphical user interface (GUI) that they developed for researchers to quantify planer cell polarity. I really appreciated that they did not just include their PCA algorithm but included the prior 2 algorithms in the software so that users of the GUI can explore for themselves the different performance of each algorithm. The authors provided a thoughtful discussion of the strengths and weaknesses of each algorithm and their appropriateness with different types of data.
Overall, I found the manuscript to be well written, thoughtful, and envision that this

© 2021. Published by The Company of Biologists under the terms of the Creative Commons Attribution License (https://creativecommons.org/licenses/by/4.0/).

QuantifyPolarity tool will be extremely valuable for the field. I think the manuscript could more- or-less be published as is. However, I do have some minor suggestions and corrections for the authors.

Response 1

Thank you for your positive comments about our research and the paper.

Comment 2

I was wondering if the author's simulation could provide insight into how the various algorithms do with different intensity patterns around the perimeter. For example, if a signal is punctate versus continuous? What if the signal localizes to tricellular vertices or only at bicellular junctions, can the algorithm identify polarity as well as a continuous distribution?

Response 2

Many thanks for these helpful suggestions.

- We have now generated new simulations using a punctate protein distribution, rather than a continuous two-level intensity. Along the same lines as the simulation shown in Figure 2A-B, we examined if polarity readouts from all methods is affected by varying cell area and shape regularity and puncta distribution using this distribution (Figure 2G-H' and Figure S2B-B'').
- We have also added a simulation to illustrate how each method behaves given tricellular localization (Figure S2C-C'').
- We have plotted all the new simulation results and provided more discussion in the revised manuscript (Lines 192-204).

Comment 3

p. 9: Here, I thought it would be helpful for the authors to show the correlation between different polarity measurements and eccentricity. Doing this would be a nice way to show that certain algorithms are affected by eccentricity, but others are less dependent. Something like Figure 6 but comparing the different algorithms.

Response 3

Thank you for the suggestion.

- In our simulated cells, we changed one variable at a time – for example, we vary the protein distribution while maintaining the eccentricity and size of the simulated cell and quantify how different polarity methods change in response to this variation. However, unlike simulated cells, *Drosophila* pupal wing cells undergo various changes at the same time, for example, changes in cell eccentricity and junctional protein distribution occur simultaneously over time. Hence, polarity magnitude could be affected by both varying cell eccentricity and protein distribution at the same time, making it difficult to attribute any changes in polarity magnitude output by a specific algorithm to either just cell eccentricity or protein distribution. Thus, although we appreciate the point the reviewer is making, different results with different methods on the biological data couldn't be simply attributed to changes in eccentricity alone.

© 2021. Published by The Company of Biologists under the terms of the Creative Commons Attribution License (https://creativecommons.org/licenses/by/4.0/).

Comment 3

p. 9: The lettering on their figure callouts for Figure 2 are incorrect. They have references for 2E, F, and G and I don't see anything beyond D in the figure.

Response 3

Apologies. We have now corrected the errors in the revised manuscript.

Comment 4

Fig. 2C: It would help to separate the blue, green, and magenta bars rather than plotting them on top of each other so that readers can compare them.

Response 4

We thank the reviewer for this suggestion.

- We appreciate that in the original images it may have been hard to distinguish the different nematics. However, it is hard to compare if we plot the nematics on different images and difficult to separate them spatially on the same image. To try to improve this, we have now displayed higher magnification images of both pupal wings in Figure 3C-D (old Figure 2C and D). We have also replotted both images by overlaying longer and thicker polarity bars. Overall, we still feel that overlaying all polarity nematics from different methods onto the same image does make it easier for visual comparison.

References

Aigouy, B., Farhadifar, R., Staple, D. B., Sagner, A., Roper, J. C., Julicher, F. and Eaton, S. (2010). Cell flow reorients the axis of planar polarity in the wing epithelium of Drosophila. *Cell* **142**, 773-786.

Aw, W. Y., Heck, B. W., Joyce, B. and Devenport, D. (2016). Transient Tissue-Scale Deformation Coordinates Alignment of Planar Cell Polarity Junctions in the Mammalian Skin. *Curr Biol* **26**, 2090-2100.

Bertet, C., Sulak, L. and Lecuit, T. (2004). Myosin-dependent junction remodelling controls planar cell intercalation and axis elongation. *Nature* **429**, 667-671.

Bulgakova, N. A., Grigoriev, I., Akhmanova, A. and Brown, N. H. (2013). Dynamic microtubules produce an asymmetric E-cadherin–Bazooka complex to maintain segment boundaries. *J Cell Biol* **201**, 887-901.

Curran, S., Strandkvist, C., Bathmann, J., de Gennes, M., Kabla, A., Salbreux, G. and Baum, B. (2017). Myosin II controls junction fluctuations to guide epithelial tissue ordering. *Dev Cell* **43**, 480-492. e486.

Devenport, D. (2016). Tissue morphodynamics: Translating planar polarity cues into polarized cell behaviors. In *Sem Cell Dev Biol*, pp. 99-110: Elsevier.

Gonzalez, R. C. and Woods, R. E. (2007). Image processing. *Digital image processing* **2**.

Merkel, M., Sagner, A., Gruber, F. S., Etournay, R., Blasse, C., Myers, E., Eaton, S. and Jülicher, F. (2014). The balance of prickle/spiny-legs isoforms controls the amount of coupling between core and fat PCP systems. *Curr Biol* **24**, 2111-2123.

Simões, S. d. M., Mainieri, A. and Zallen, J. A. (2014). Rho GTPase and Shroom direct planar polarized actomyosin contractility during convergent extension. *J Cell Biol* **204**, 575-589.

Zallen, J. A. and Wieschaus, E. (2004). Patterned gene expression directs bipolar planar polarity in Drosophila. *Dev Cell* **6**, 343-355.

© 2021. Published by The Company of Biologists under the terms of the Creative Commons Attribution License (https://creativecommons.org/licenses/by/4.0/).

Second decision letter

MS ID#: DEVELOP/2020/198952

MS TITLE: QuantifyPolarity, a new tool-kit for measuring planar polarized protein distributions and cell properties in developing tissues

AUTHORS: Su Ee Tan, Weijie Tan, Katherine H Fisher, and David Strutt

I have now received all the referees reports on the above manuscript, and have reached a decision. The referees' comments are appended below, or you can access them online: please go to BenchPress and click on the 'Manuscripts with Decisions' queue in the Author Area.

The overall evaluation is positive, but one of the reviewers (Rev2) still raises a few issues that should be addressed as best you can to clarify before we can proceed with acceptance. Please attend to the reviewers' comments in your revised manuscript and detail them in your point-by-point response. If you do not agree with any of their criticisms or suggestions explain clearly why this is so.

Reviewer 1

Advance summary and potential significance to field

The authors have responded to all of the reviewers' concerns. The revised manuscript has several additions and clarifications that substantially improve the manuscript. Major revisions include 1) a summary figure and table illustrating the differences between the three methods for measuring polarity, 2) additional tests on simulated cells that better mimic real data (punctate, noisy variations in signal intensity), and 3) an elaborated methods section. Overall the side-by-side comparisons of different polarity methods and the graphical user interface that integrates all three methods will be incredibly useful for the polarity field.

Comments for the author

The authors have sufficiently addressed all of our comments.

Reviewer 2

Advance summary and potential significance to field

While we think that the manuscript has somewhat improved, fundamental problems remain. Moreover, checks of the analyses carried out by the authors revealed further problems.
The field has much to gain from tools such as provided by this publication and from a comparison of different polarity methods. However, we feel that the proposed PCA method still has serious flaws, which prevent us from recommending publication.

Comments for the author

While we think that the manuscript has somewhat improved, fundamental problems remain. Moreover, checks of the analyses carried out by the authors revealed further problems.

The field has much to gain from tools such as provided by this publication and from a comparison of different polarity methods. However, we feel that the proposed PCA method still has serious flaws, which prevent us from recommending publication.

To discuss what we perceive as flaws in the proposed PCA method, we will separately discuss its two parts:
1. The eccentricity of the cell to be analyzed is removed by a geometric operation (the authors call this "compression").

© 2021. Published by The Company of Biologists under the terms of the Creative Commons Attribution License (https://creativecommons.org/licenses/by/4.0/).

2. The subsequent quantification of cell polarity using equations 4-14.
We think that both steps are independent of one another.

1. "compression" step:

a) This step has not been explained in the original version of the manuscript, even though we think that it is the most important step in what the authors propose. We also think that this can in principle be a reasonable "preprocessing" step depending on the tissue under study and the scientific question asked.

However, this "preprocessing" step could also be used before any of the two other two methods (Ratio and Fourier) as well. By construction, this would lead to a similar type of shape independence that the authors advertise for their PCA method.

Proposing such a preprocessing step to the community makes sense.

b) However, from the methods part, we could not figure out how exactly it is done by the authors and their toolbox. To "compress" a given cell, is the image data transformed, which would lead to a degradation of the raw data? Or is the transformation carried out purely mathematically on the angles theta (which would not affect image quality)?

We found the corresponding methods section confusing, as it said that after the "compression" step, "intensity of the protein of interest is extracted from original image at an angle on the segmented cell boundary" (lines 575-577). If the intensity of the *original* image is probed at angle *theta*, then how would the compression step matter?

c) We reiterate that it is highly problematic to advertise something as eccentricity/shape independent, even though there can be different definitions of shape independence. We already clarified this in our previous comment number 5 and the authors agreed. So we were surprised that there is still no discussion of different kinds of shape independence in the text.

To demonstrate this issue, we attached results of the authors' toolbox that we obtained on an example cell. Indeed, the authors' proposed compression step can lead to surprises. As an example, take polarity1.png, where almost all of the polarity protein is on the horizontal interfaces. However, the PCA-quantified polarity nematic does not reflect this - due to the "compression" step, the polarity protein on the horizontal interfaces is weighted less, resulting in an almost zero total polarity nematic (compare Fourier example polarity2.png). This would be surprising to many biologists and an unwanted property of the quantification method. In fact, some would even perceive the authors' method as strongly eccentricity dependent. While this property might be desirable in other situations, a clear discussion is required. E.g. the authors choose to keep the polarity *intensity* constant during the compression step, instead of keeping the *total* polarity amount on each bond constant, which corresponds to different definitions of "eccentricity/shape independence". In the current manuscript, it does not become clear that there are these different choices and what their consequences are. However, this would be required for a proper comparison of different polarity quantification methods. Claims like one method is "eccentricity independent" or "shape independent" while another one is not should be accompanied by a discussion that there are different definitions of shape/eccentricity independence, resulting in different answers to the question of which polarity measure is eccentricity independent.

© 2021. Published by The Company of Biologists under the terms of the Creative Commons Attribution License (https://creativecommons.org/licenses/by/4.0/).

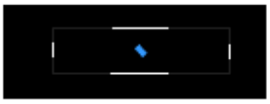

Polarity1.png

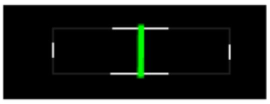

Polarity2.png

2. Subsequent PCA analysis (Eqs. 4-14):

Many of our original concerns remain. Here we focus on the two most relevant ones:

a) As we mentioned before, the polarity angle from the PCA method depends on the image brightness scaling. While the authors tested this with simulations, these simulations were carried out on perfectly mirror symmetric polarity distributions, which will of course give the expected polarity angle of zero always. This changes however as soon as the polarity distribution is not mirror symmetric any more.

We now explicitly tested this, see angles_sketch.png, where we consider a simple toy polarity distribution as indicated by the sketch. The phenomenon we describe is generic and should appear for any distribution (of e.g. puncta) of different intensity (*more* than 2 levels) along the cell outline in a way that is *not* mirror symmetric.

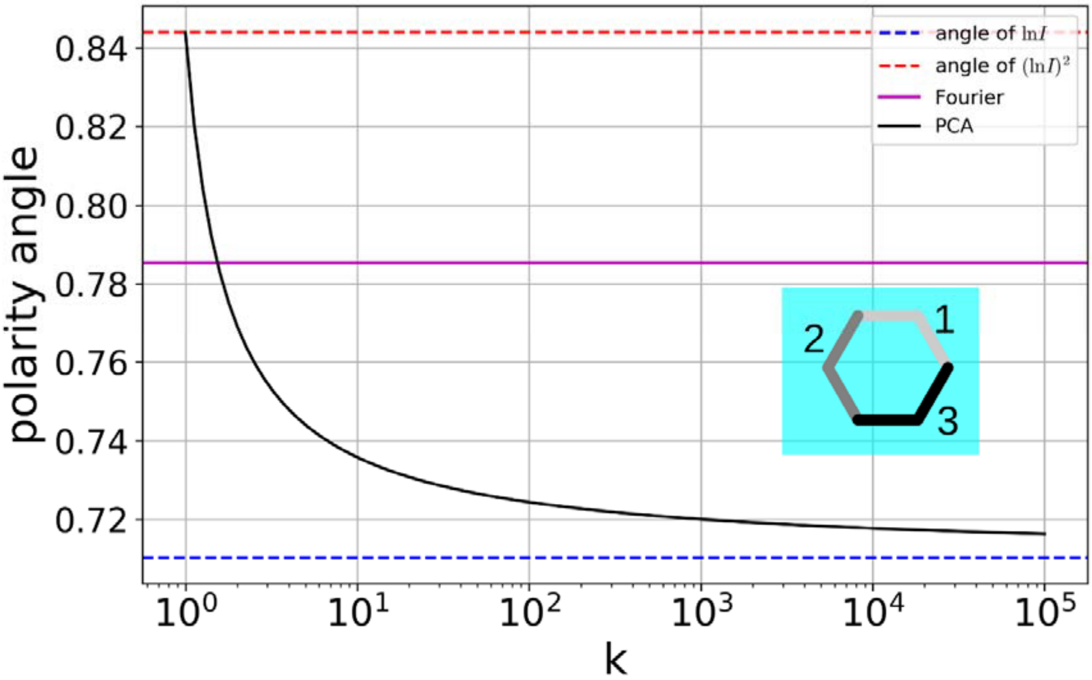

angles_sketch.png

We find that indeed the angle obtained using the proposed PCA method depends on the brightness scaling factor (which is the same as a dependency on k in Eq. 5), and that it interpolates between the angles of the ln I and (ln I)^2 distributions. Regarding the argument of the authors in their reply: both distributions ln I and (ln I)^2 certainly have corresponding maxima and minima. However, the PCA method does not only depend on the maxima and minima, but also on any other values. This is also the reason that this effect only becomes visible for >2 different intensity levels. This stresses again the need for more realistic tests. (The puncta the authors added in their revision also seem to use just 2 different intensity levels.)

© 2021. Published by The Company of Biologists under the terms of the Creative Commons Attribution License (https://creativecommons.org/licenses/by/4.0/).

b) It is still not clear to us in what situations the non-linearities applied in the PCA method as compared to the Fourier method provide advantages. Performance reasons brought forward by the authors are an independence of cell shape (e.g. new Fig. 2C,D), which is clearly just due to the preprocessing step.

The other line of argumentation of the authors is the new Fig. 2E (old Fig 1H), i.e. the position of the peak of the polarity norm. On close inspection, we were a bit surprised by the claimed "symmetry of the polarity strength curve" of the PCA method, as this will clearly depend on the baseline and peak values in these toy examples. Indeed, our own calculations confirm this (compare to the file amplitudes.png). Moreover, while the curve of the normalized Fourier method (Eqs. 2 and 3) is indeed more skewed than the curve for the PCA method, the non-normalized Fourier method (no factor 1/N in Eqs. 2 and 3) is naturally symmetric (with only a very slight deviation due to the hexagonal cell shape), where the curve shape is independent on baseline and peak values.

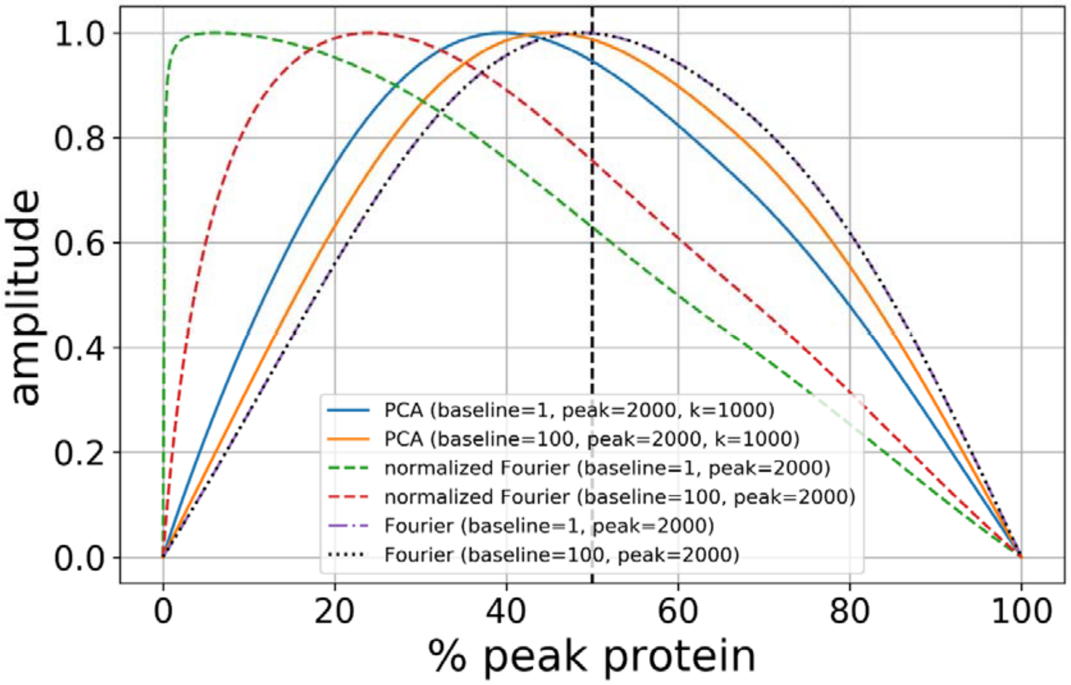

amplitudes.png

Hence, the advertised advantage of the PCA method already disappears when varying the baseline or peak values. Also, if the goal is to have a symmetric curve in this toy experiment, better methods exist already (e.g. non-normalized Fourier, *which corresponds to the PCA method without the non- linearities*, see our previous comment 2).

c) In addition to this, the non-linearities (Eq 5, Eq 14, our previous comment 2) of the proposed PCA method also introduce other problems, e.g. a failing of the method if only even a single pixel along the cell outline has intensity value zero, because of the logarithm in Eq. 5. More generally, the purpose of the log in Eq. 5 remains unclear. The reply to comment 3 of referee 1 in this regard is confusing - what is happening in Eq. 5 is not a normalization. Eq. 5 merely makes the measure more insensitive to the intensity values. More precisely, the log distorts the intensity values along the cell outline before computing the polarity from them. Why this distortion should be desirable is not explained.

Taken together, we believe that providing the computational tool and proposing the preprocessing step as an option could be a welcome contribution to the field *if* the dependence on the definition of "eccentricity/shape independence" is better discussed.

© 2021. Published by The Company of Biologists under the terms of the Creative Commons Attribution License (https://creativecommons.org/licenses/by/4.0/).

However, we think that the remaining part of the PCA method (i) amounts to being a mere modification of the Fourier method without being stated, and (ii) the modifications introduce problems into the method. The authors have tried to clarify where these modifications are useful, but as discussed above, their argumentation is not convincing. Unless it is clear in what situations and why the modifications applied to the Fourier method are useful, we cannot recommend publication.

Minor points:

-We had serious trouble running the tool on windows, we found a solution here:

https://www.mathworks.com/matlabcentral/answers/98050-why-do-i-get-an-error-saying-undefined-function-or-variable-matlabrc-when-executing-a-program-th
we think the authors should either fix their tool or add this link to the README of their github page, so that users can find it.
-We think that in order for reviewers to evaluate a software, they should be given access to the code of the tool, we note that for the second time we were not given access to it.

Reviewer 3

Advance summary and potential significance to field

The authors have systematically examined a number of methods for quantifying planar cell polarity in a tissue.
They have provided a comprehensive comparison of these methods, which will be a useful resource in the field.

Comments for the author

The authors have done a nice job addressing the reviewer comments. I think the manuscript is ready for publication.

Second revision

Author response to reviewers' comments

Response to Reviewer – Overview

General comments

We would like to thank the reviewer once again for engaging so enthusiastically with our work, and helping us to further improve the manuscript.
In their second review, the Reviewer raised important issues, which we agree merited further investigation, as if true, they would cast doubt on the usefulness of the QuantifyPolarity Tool. However, we are happy to report that having looked into them, they seem to have arisen from misunderstandings or possibly mistakes. In particular, we believe our analysis clearly refutes their assertion that *"we think that the remaining part of the PCA method (i) amounts to being a mere modification of the Fourier method without being stated, and (ii) the modifications introduce problems into the method"* which appears to be their main argument against publication.
In response to their feedback we have further considered the ramifications of different definitions of polarity, and more extensively compared the different methods of measuring polarity. We are pleased to say this additional work has further confirmed the importance of defining polarity relative to *average* protein levels on cell junctions (the default route used in the prior literature) and that our new PCA method displays additional favorable characteristics over e.g. the Fourier method, further strengthening the case for its

© 2021. Published by The Company of Biologists under the terms of the Creative Commons Attribution License (https://creativecommons.org/licenses/by/4.0/).

widespread adoption.
A comment-by-comment response to the concerns of the reviewer is provided at the end of this document. Below we provide a high-level summary for the benefit of the editor and other readers, followed by a detailed list of characteristics of our PCA method that make it advantageous over the existing Normalized Fourier Series or Ratio Methods.
We have also added some additional simulation data to the manuscript (Figures 2F-F' and S1F-G') that further demonstrate desirable characteristics of our PCA method. To make space for this, there has also been some other minor editing to the manuscript.

Summarized response to second-round comments of Reviewer 2

• In their feedback, Reviewer 2 reports several simulations to compare the PCA method to the Fourier method by (we believe) recoding the PCA and Fourier Series algorithms. However, none of their results show the same behavior as our QuantifyPolarity GUI, possibly due to differing implementation of both PCA and Fourier methods. In particular, when we perform the same simulations with QuantifyPolarity we did not get same results as provided by the Reviewer (i.e. both analysis and simulation results *polarity1.png* and *angles_sketch.png*). Since no details on simulation parameters or script were provided, it is impossible for us to verify what has gone wrong. We have now provided a set of simulated images (including our simulation results) that we used to generate these results where users can easily run their own tests using our QuantifyPolarity GUI.
https://drive.google.com/drive/u/1/folders/1bBv6Dxf5jOm-VVpk6fNRFIoXGaxmufS4 (The link above is provided in our github page)

• To our minds, a key issue is the repeated assertion of the Reviewer that the non-normalized Fourier Series method is the most appropriate tool, rather than our PCA method or the normalized Fourier Series method (as implemented in our GUI) since it exhibits a symmetrical polarization strength profile independent of low base intensity. Merkel et al., 2013 modified the non-normalized Fourier Series from (Aigouy et al., 2010) to make it insensitive to variation in intensity by normalizing it – henceforth referred to as the Fourier Series method in our QuantifyPolarity GUI. Such normalization is crucial to ensure the robustness of the method against variation in biological image intensity due to different microscope settings, immunolabeling protocols and so on. However, the reviewer suggests use of the non-normalized Fourier Series method while failing to acknowledge that it is adversely affected by intensity scaling and hence wouldn't be a suitable tool even though it possesses a symmetrical polarization profile. In this scenario, the PCA method provides the best of both worlds – being insensitive to image intensity scaling and exhibits a largely symmetrical polarization curve. We also showed that while an extremely low base intensity (1 a.u.) does slightly shift the peak polarity strength of the PCA method, however the symmetrical profile is largely unaffected. As for the Fourier Series method, we found that this method not only exhibits a skewed polarization strength profile but this skewness is further adversely affected by decreasing base intensity.

• Additionally, as a result of further simulations prompted by the comments of the Reviewer, we have found another important property of PCA method which makes it highly preferable over the existing methods. One of the most important properties of a polarity method is being able to detect variation in peak-to-base protein intensity. Given a cell with constant base protein intensity on horizontal junctions, increasing amount of peak protein on both vertical junctions should result in increasing polarity magnitude as it implies more proteins are being asymmetrically localized to the vertical junctions (Figure 1G''). We performed simulations of cells with increasing peak protein intensity while maintaining a constant base intensity, and as expected polarity magnitude from the PCA method increases with increasing relative peak-to-base intensity (new Figure 2F). On the contrary, polarity magnitude output from both the Ratio and Fourier Series method gradually increases and then plateaus even though there is significantly more peak protein (new Figure 2F). Hence the PCA method is more sensitive in detecting different degrees of polarization strength due to varying peak-to-base intensity as compared to both the Ratio and Fourier Series methods.

• In the eyes of the Reviewer, it seems that the most "controversial" modification that we have implemented in the PCA method is the introduction of a logarithmic term, which is

© 2021. Published by The Company of Biologists under the terms of the Creative Commons Attribution License (https://creativecommons.org/licenses/by/4.0/).

necessary for intensity normalization. Moreover, the Reviewer asserted that polarity angle from the PCA method will be affected by varying image intensity scaling. To test this, we have simulated various scenarios with varying intensity scaling (see Table 2) and found no evidence that the polarity angle output from PCA method can be affected by varying intensity scaling. In fact, the PCA method outperformed the Fourier Series method in all simulations, suggesting that the modifications to the PCA method are in fact beneficial for planar polarity quantification.

Responses to further concerns of the Reviewer are listed below:

1) The Reviewer is concerned that *"The puncta the authors added in their revision also seem to use just 2 different intensity levels"*. We are happy to clarify that as stated in the manuscript each punctum intensity ranges from 40 to 255 a.u (Figure 1G' and zoom in of puncta in Figure S2B and S2C).

2) The Reviewer argues that *"the nonlinearities due to logarithm functions introduced other problems"* such as failure of the PCA method given intensity values of 0 a.u. We did actually address this concern in our previous response as follows *"As the logarithmic function is not defined when $I_i = 0$, thus the intensity value is set to 1 if it is 0, hence $I_i \in \mathbb{N}^+$"*. Moreover, the Reviewer claimed that *"the nonlinearities logarithm function is designed to make the PCA method insensitive to variation in intensity and this is not a normalization process"*. We believe that the mathematical formulation of making a method insensitive to variation in intensities simply means normalization – however, this seems to be a semantic issue rather than an issue with the behavior of the PCA method.

3) The Reviewer argues the following *"we think that the remaining part of the PCA method (i) amounts to being a mere modification of the Fourier method without being stated, and (ii) the modifications introduce problems into the method"*. First of all, we have clearly emphasized in our previous Response 2 to Reviewer 2 that while Fourier Series and standard PCA can be converted to each other mathematically (however note that they are not subset of each other), using a standard PCA method would suffer several limitations similar to the Fourier method, which causes biased polarity quantification results. Therefore, in this work, we developed novel equations to further enhance the standard PCA-based method (see previous Response 3 to Reviewer 1), so that it would exhibit more desirable features for planar polarity quantification. In fact, the following statement from the Reviewer also seems to support the view that the PCA method is not a mere modification from the Fourier Series method: *"It is still not clear to us in what situations the non-linearities applied in the PCA method as compared to the Fourier method provide advantages. Performance reasons brought forward by the authors are an independence of cell shape (e.g. new Fig.2C,D), which is clearly just due to the preprocessing step"*. It is important to note that if the cell compression step is added to the Fourier Series method, this method will never behave similarly to the PCA method and will still inevitably suffer from several other limitations – in particular failure to detect different degrees of polarization strength due to varying relative peak-to-base protein intensity and exhibiting an asymmetrical polarization strength curve that can be further skewed by decreasing intensity. (See Table 1 for a summary of side-by-side performance comparison of the Fourier Series and PCA methods.)

Table 1: A summary of performance comparisons between the Fourier Series and PCA methods

Fourier Series	PCA
Fourier Series polarity magnitude is affected by varying cell eccentricity (Figure 2C, 2D)<br>Fourier Series polarity angle is affected by varying cell eccentricity (Figure 4F')	PCA polarity magnitude is not sensitive to varying cell eccentricity (Figure 2C, 2D)<br>PCA polarity angle is not affected by varying cell eccentricity (Figure 4F')

© 2021. Published by The Company of Biologists under the terms of the Creative Commons Attribution License (https://creativecommons.org/licenses/by/4.0/).

Fourier Series fails to detect different degree of polarization strength due to varying junctional protein distribution on elongated cells<br>(Figure R4B or Figure 2F)<br>See Response 1 to Comment 1	PCA method can robustly detect different degrees of polarization strength due to varying junctional protein distribution on elongated cells<br>(Figure R4B or Figure 2F)<br>See Response 1 to Comment 1
Non-normalized Fourier Series polarity magnitude is extremely sensitive to intensity scaling - hence not recommended for usage in polarity quantification<br>(Figure R6)<br>See Response 2 to Comment 2<br>Fourier Series polarity magnitude and angle are not affected by intensity scaling<br>(Figure S1A-A’, Figure R5)<br>See Response 2 to Comment 2	PCA polarity magnitude and angle are not affected by intensity scaling<br>(Figure S1A-A’’, Figure S2A and A’’, Figure R5, Figure R6)<br>See Response 2 to Comment 2
Fourier Series exhibits an asymmetrical or skewed polarization strength curve, that can be further affected by decreasing base intensities<br>(Figure R7)<br>See Response 2 to Comment 2	PCA method yields a symmetrical polarization strength curve - slightly affected by extreme low base intensities (1a.u.)<br>(Figure R7)<br>See Response 2 to Comment 2
Fourier Series fails to detect change in polarization strength/magnitude in response to increasing relative peak-to-base intensity (for high range of relative peak-to-base intensity ratios)<br>(Figure R8B)<br>See Response 2 to Comment 2	PCA polarity magnitude linearly increases in response to increasing relative peak-to-base intensity with same protein distribution<br>(Figure R8B)<br>See Response 2 to Comment 2

Here we provide a complete table of performance comparisons between the Fourier Series and PCA methods. From this comparison, it is evident that the Fourier Series method is (1) sensitive to cell shape/eccentricity, (2) fails to detect different degrees of polarization strength based on different junctional protein distributions on elongated cells and (3) relative peak-to-base protein intensity, and (4) exhibits an asymmetrical polarization strength curve that can be further skewed by decreasing intensity. Based on these measures, we argue that the PCA method provides overall more advantageous characteristics in most usage scenarios.

Response 1 to Comment 1

1. "compression" step:

a) This step has not been explained in the original version of the manuscript, even though we think that it is the most important step in what the authors propose. We also think that this can in principle be a reasonable "preprocessing" step depending on the tissue under study and the scientific question asked. However, this "preprocessing" step could also be used before any of the two other two methods (Ratio and Fourier) as well. By construction, this would lead to a similar type of shape independence that the authors advertise for their PCA method. Proposing such a preprocessing step to the community makes sense.

As explained in the responses to reviewers, this step was indeed omitted by error in our first draft. However, it has been explained in the Materials and Methods section in our second draft.

© 2021. Published by The Company of Biologists under the terms of the Creative Commons Attribution License (https://creativecommons.org/licenses/by/4.0/).

We agree it might be useful to have cell compression as a preprocessing step for the Fourier Series and Ratio methods, in addition to the PCA method, so that these other methods would also enjoy being eccentricity independent. However, their behavior would then differ from the published algorithms, and we think it is useful for users to have access to the previously published methods so that they can compare to our new PCA method all within a single GUI. We will consider adding this feature in the future if we find there is demand. However, as there are other disadvantages to the Fourier and Ratio methods (as described here and in the manuscript), we believe it would be better for users to adopt our new PCA method and only use the Fourier and Ratio methods if they have a specialized application. Also, since this is an open source GUI, users can also modify the script as they wish.

b) However, from the methods part, we could not figure out how exactly it is done by the authors and their toolbox. To "compress" a given cell, is the image data transformed, which would lead to a degradation of the raw data? Or is the transformation carried out purely mathematically on the angles theta (which would not affect image quality)? We found the corresponding methods section confusing, as it said that after the "compression" step, "intensity of the protein of interest is extracted from original image at an angle on the segmented cell boundary" (lines 575-577). If the intensity of the *original* image is probed at angle *theta*, then how would the compression step matter?

We apologize if the cell compression step was not clear. We have now added the entire cell compression equation in our Materials and Methods section. Firstly, each pixel on the cell boundary is extracted as points with corresponding coordinates and intensity value. The compression step is then carried out on these points mathematically. Hence, there is no degradation on the image quality as number of pixels/points on the cell boundary is conserved.

Thank you for noticing the typo (original lines 575-577).

Revised text in Materials and Methods:

In order to compensate for elongated cells, the cell is negatively stretched (or compressed). Each pixel on the cell boundary is represented as $(x_j y_j I_j)$, where $x_j y_j$ represent the x- and y-coordinates of the pixel and I_j the intensity of that pixel. An ellipse is fitted to obtain the orientation φ, major and minor axes a, b of the cell. For each of these points, it undergoes the following transformation:

$$T(x_i, y_i) = \begin{bmatrix} x_i' \\ y_i' \end{bmatrix} = \mathbf{R}(\varphi)\mathbf{C}\left(\frac{b}{a}\right)\mathbf{R}(-\varphi)\left(\begin{bmatrix} x_i \\ y_i \end{bmatrix} - \begin{bmatrix} \bar{x} \\ \bar{y} \end{bmatrix}\right), \quad \text{Eqn 4}$$

where $\begin{bmatrix} x_i' \\ y_i' \end{bmatrix}$ is the transformed (compressed) coordinates relative to the cell centroid $\begin{bmatrix} \bar{x} \\ \bar{y} \end{bmatrix}$, $\mathbf{R}(\theta)$ is the rotation matrix with the rotation angle of θ, and $\mathbf{C}(\alpha)$ is the compression matrix with the compression factor α, with $\alpha < 1$. Both of these matrices can be written as

$$\mathbf{R}(\theta) = \begin{bmatrix} \cos\theta & -\sin\theta \\ \sin\theta & \cos\theta \end{bmatrix}, \quad \text{Eqn 5}$$

$$\mathbf{C}(\alpha) = \begin{bmatrix} \alpha & 0 \\ 0 & 1 \end{bmatrix}. \quad \text{Eqn 6}$$

Note that although this operation reduces the cell area, it has been shown in Fig.2A-A' that polarity readout is not affected by cell area.
Next, angle θ_i is computed based on the transformed coordinates x_i', y_i', with respect to the centroid of the cell

c) We reiterate that it is highly problematic to advertise something as eccentricity/shape

© 2021. Published by The Company of Biologists under the terms of the Creative Commons Attribution License (https://creativecommons.org/licenses/by/4.0/).

independent, even though there can be different definitions of shape independence. We already clarified this in our previous comment number 5 and the authors agreed. So we were surprised that there is still no discussion of different kinds of shape independence in the text.

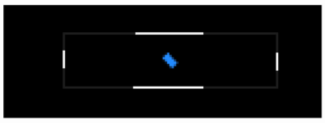

Polarity1.png

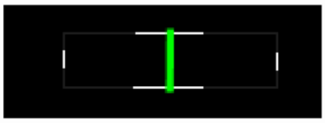

To demonstrate this issue, we attached results of the authors' toolbox that we obtained on an example cell. Indeed, the authors' proposed compression step can lead to surprises. As an example, take polarity1.png, where almost all of the polarity protein is on the horizontal interfaces. However, the PCA-quantified polarity nematic does not reflect this - due to the "compression" step, the polarity protein on the horizontal interfaces is weighted less, resulting in an almost zero total polarity nematic (compare Fourier example polarity2.png). This would be surprising to many biologists and an unwanted property of the quantification method. In fact, some would even perceive the authors' method as strongly eccentricity dependent. While this property might be desirable in other situations, a clear discussion is required. E,g, the authors choose to keep the polarity *intensity* constant during the compression step, instead of keeping the *total* polarity amount on each bond constant, which corresponds to different definitions of "eccentricity/shape independence". In the current manuscript, it does not become clear that there are these different choices and what their consequences are. However, this would be required for a proper comparison of different polarity quantification methods. Claims like one method is "eccentricity independent" or "shape independent" while another one is not should be accompanied by a discussion that there are different definitions of shape/eccentricity independence, resulting in different answers to the question of which polarity measure is eccentricity independent.

Below we provide the definition of polarity magnitude and explanation with examples on why polarity magnitude is determined based on *average* junctional protein and not *total* junctional protein so that the readout is shape independent.

Allow us to first elaborate on the concept of how polarity magnitude or strength is defined in our manuscript and consistent with the extensive prior literature (see refs in manuscript). As defined in our manuscript, polarity magnitude is determined based on the junctional protein distribution and relative peak-to-base intensity. A simpler way to understand polarization strength is based on the concept from the ratio method: polarity magnitude is determined by computing the *average* junctional protein on horizontal versus vertical junctions (Farrell et al., 2017; Duda et al., 2019), where a cell is considered polarized when the *average* amount of protein on horizontal junctions is higher than *average* amount of protein on vertical junction.

Note that, as longer cell junctions usually exhibit higher *total* amount of proteins (e.g. in the unpolarized state), it is therefore essential to compute the *average* total junctional protein per unit junction length so that the polarity readout is independent of junctional length/cell eccentricity. By taking the average junction intensity, the ratio or polarity readout is not be affected by different junction length or elongation (hence, allowing comparison between cells with varying geometry), and thus reflects the polarization state of the cell. However, as explained in our manuscript, such ratio methods as implemented in (e.g. Farrell et al., 2017; Duda et al., 2019) is insufficient to provide an unbiased measure of polarity when junctional proteins are not polarizing along a specific axis or for cells that are irregular in geometry.

In the example of simulated cell provided by the Reviewer, we note that while the *total* or

© 2021. Published by The Company of Biologists under the terms of the Creative Commons Attribution License (https://creativecommons.org/licenses/by/4.0/).

absolute amount of protein on horizontal junctions is more than the vertical junctions, the *average* or relative amount of protein on horizontal and vertical junctions is approximately identical (in the example provided, qualitatively there is approximately 1/3 of both horizontal and vertical junction length are occupied with protein). Therefore, we disagree that the provided example cell should be considered as polarized by the definition most widely adopted in the prior literature. To better illustrate the importance of considering *average* junctional protein over *total* junctional protein in quantification of polarity, we provided several different cases in Figure R1 and R2 using the simple ratio method.

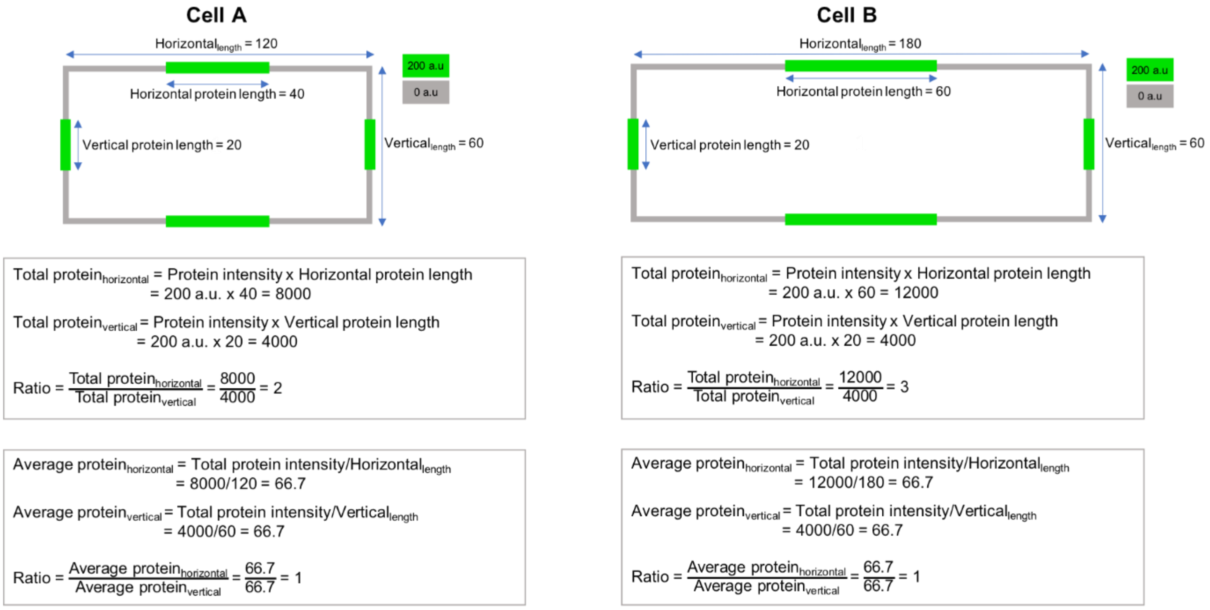

Figure R1: Examples of simulated cells with different elongation where cell B is more elongated than cell A. Each cell exhibits protein (in green) homogenously localized on 1/3 of each junction length - hence by our definition should exhibit zero polarity. Despite, Cell B being more eccentric than Cell A, the average protein for all Cell B junctions are similar to Cell A - where the protein occupied 33.3% of the entire junction length. Hence, these cells should exhibit a similar polarity readout despite being more elongated. However, by computing the ratio of *total* protein on horizontal to vertical junctions, both cells appear polarized, and furthermore cell B exhibits a higher ratio (or polarity) value than cell A. Therefore, without averaging of the total protein intensity to its junction length, the polarity readout is a function of cell elongation. Conversely, by taking the ratio of average protein on horizontal to vertical junctions, cell A and B appear unpolarized, independent of cell elongation.

Hence, just by considering the *total* amount of protein on each junction instead of *average* amount of protein on each junction as suggested by Reviewer 2, it would indeed result in higher polarity readout. However, this polarity readout is biased by different cell elongation because more elongated cells with higher total amount of proteins would appear more polarized as compared to less elongated cells given the same protein distribution. In Figure R2 below, we provided another simple example to illustrate the importance of considering the average protein rather than total amount of proteins per junction.

© 2021. Published by The Company of Biologists under the terms of the Creative Commons Attribution License (https://creativecommons.org/licenses/by/4.0/).

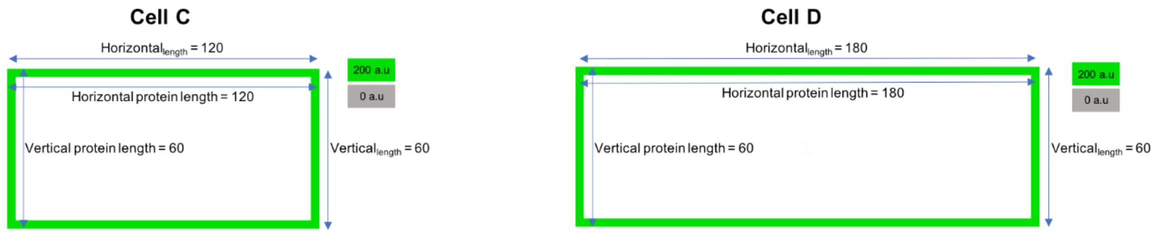

Cell C:

Total protein$_{horizontal}$ = Protein intensity x Horizontal protein length = 200 a.u. x 120 = 24000

Total protein$_{vertical}$ = Protein intensity x Vertical protein length = 200 a.u. x 60 = 12000

$$\text{Ratio} = \frac{\text{Total protein}_{horizontal}}{\text{Total protein}_{vertical}} = \frac{24000}{12000} = 2$$

Cell D:

Total protein$_{horizontal}$ = Protein intensity x Horizontal protein length = 200 a.u. x 180 = 36000

Total protein$_{vertical}$ = Protein intensity x Vertical protein length = 200 a.u. x 60 = 12000

$$\text{Ratio} = \frac{\text{Total protein}_{horizontal}}{\text{Total protein}_{vertical}} = \frac{36000}{12000} = 3$$

Cell C:

Average protein$_{horizontal}$ = Total protein intensity/Horizontal$_{length}$ = 24000/120 = 200

Average protein$_{vertical}$ = Total protein intensity/Vertical$_{length}$ = 12000/60 = 200

$$\text{Ratio} = \frac{\text{Average protein}_{horizontal}}{\text{Average protein}_{vertical}} = \frac{200}{200} = 1$$

Cell D:

Average protein$_{horizontal}$ = Total protein intensity/Horizontal$_{length}$ = 36000/180 = 200

Average protein$_{vertical}$ = Total protein intensity/Vertical$_{length}$ = 12000/60 = 200

$$\text{Ratio} = \frac{\text{Average protein}_{horizontal}}{\text{Average protein}_{vertical}} = \frac{200}{200} = 1$$

Figure R2: Example of simulated cells with homogeneous protein level on all junctions where cell D is more elongated than cell C.

As defined in the manuscript, when proteins are homogenously distributed to all junctions, cells should be non-polarized (with ratio of 1). In these simulated cells, it is evident that by computing the ratio of total protein on horizontal to vertical junctions, cells C and D exhibit polarity readouts of 2 and 3 respectively (Figure R2). In fact, cell D exhibits higher polarity than cell C simply because there is more total protein on its horizontal junctions (due to longer junction length) as compared to cell C. On the contrary, by taking the ratio of average protein on horizontal to vertical junctions, both cells C and D equally exhibit a ratio of 1 which corresponds to a non-polarized state.

In short, we would like to emphasize that polarity magnitude is determined based on *average* junctional protein and not *total* junctional protein. By considering *total* amount of protein, the measure becomes inherently shape dependent, as even for a homogenous protein distribution, longer junctions exhibit more total protein and vice versa.

As the definition of "polarity" that we are using has evidently caused confusion (and arguably we failed to be sufficient explicit on the point), we have added the following statement to clarify our definition of cell shape independence.

Lines 79-87:
The definition of cell shape independence is adopted from the ratio method of quantifying polarity, where polarity magnitude is computed as the ratio of *average* protein on opposite junctions, rendering this method independent of junction length and hence cell elongation. If instead *total* protein on a cell junction is considered, even with a homogeneous protein distribution, more elongated cells will appear more polarized than less elongated ones simply because longer junctions have higher total protein. Similarly, larger cells should not appear more polarized than smaller ones and polarity angle should be oriented on the axis of maximum asymmetry, unaffected by cell geometry.

Below we provide a full simulation of varying cell eccentricity to demonstrate that the Fourier Series method is sensitive to cell elongation, while the PCA method is insensitive to cell elongation and produces the expected polarity based on average protein on junctions.

Given that an epithelial tissue is often comprised of cells with varying eccentricity, it is insufficient to compare and draw the conclusion that the PCA method displayed "eccentricity dependent" behavior as compared to the Fourier Series method merely based on a single

© 2021. Published by The Company of Biologists under the terms of the Creative Commons Attribution License (https://creativecommons.org/licenses/by/4.0/).

elongated example cell.

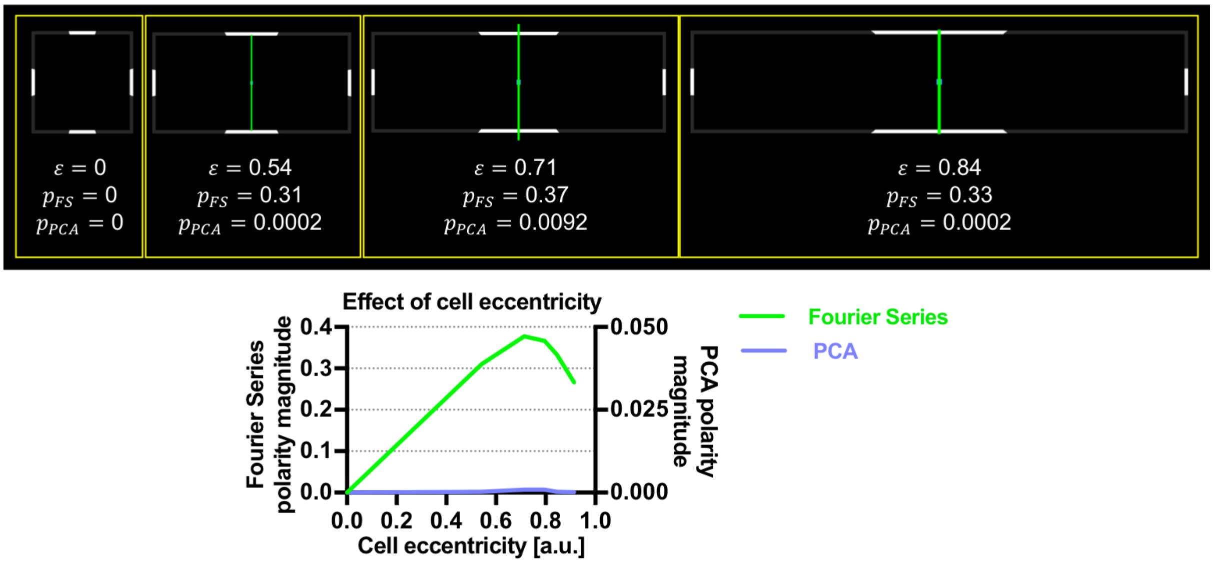

Figure R3: Effect of varying cell eccentricity on polarity magnitude.
Cell eccentricity is denoted by ε and polarity magnitude for Fourier Series and PCA is denoted by p_{FS} and p_{PCA} respectively.

Hence, we have simulated similar cells with varying eccentricity (ε) where we can fairly compare the performance between the Fourier Series and PCA method and draw a more conclusive result as to which method is “eccentricity dependent” (Figure R3). As shown in our simulation, the more elongated a cell is, the higher total amount of protein we can find on the horizontal junctions, consistent with what Reviewer’s observation *“where almost all of the polarity protein is on the horizontal interfaces”*. So if by following the prediction from Reviewer 2, more elongated cells should exhibit higher polarity readout since they have more total proteins on their horizontal junctions as compared to a less elongated cell (although we would like emphasize that we do not agree polarity magnitude should be determined based on total amount of proteins on the junctions as this can be biased by cell eccentricity). However, we found that polarity magnitude obtained from the Fourier Series method (p_{FS}) significantly decreases (30%) in a cell with higher eccentricity of 0.9 as compared to a cell with lower eccentricity of 0.71. From this result, it is evident that having higher “total amount of protein” on specific junctions does not necessarily give higher Fourier Series polarity magnitude that the Reviewer expected.

Furthermore, we contend that the proper and more logical way to interpret polarity is using the *average* amount of protein on each junction. Since, the average amount of protein on each junction remained constant when we stretch the cell, where the protein occupied 33.3% of the entire junction length, these cells should exhibit similar polarity readout (non-polarized) despite being more elongated. The polarity magnitude output from the PCA method (p_{PCA}) indeed remained close to zero, indicating non-polarized.

Below we provide another simulation to show that the sensitivity of both Ratio and Fourier Series methods to cell eccentricity can result in failure to detect changes in junctional protein distribution on elongated cells.

We think the Reviewer is arguing that by conserving total amount of protein while elongating a cell, both Fourier Series and Ratio methods should result in “eccentricity independent” behavior. However, this seems to overlook the fact that junctional protein distribution is also changing/varying during cell elongation. Our new simulation (Figure R4) resembles the simulation in Figure 2E, except that now junctional protein distribution varies on an elongating cell rather than on a regular cell as in Figure 2E. A good polarity method should be able to detect changes in junctional protein distribution independently of cell elongation. While both the Fourier Series and Ratio methods can detect changes in junctional protein

© 2021. Published by The Company of Biologists under the terms of the Creative Commons Attribution License (https://creativecommons.org/licenses/by/4.0/).

distribution on a regular cell (see Figure 2E), evidently these methods failed to detect changes in junctional protein distribution on cells that are at the same time changing their elongation, due to inherent sensitivity to cell eccentricity of these methods (Figure R4).

For simplicity, let us use the same concept from the ratio method explained above on these simulated cells (Figure R4A). If we compute the ratio of *average* protein on vertical to horizontal junctions on these cells, cell 1 would exhibit much higher ratio or polarity magnitude than cell 2 (Figure R4A). This is in line with our definition of polarity strength based on junctional protein distribution, where polarity magnitude decreases when there are more proteins covering both vertical and horizontal junctions as shown in Figure R4C (see Definition of Polarity Magnitude in Line 70-77 and Figure 1G and Figure 2E).

On the contrary, polarity magnitude obtained from both the Ratio and Fourier Series methods remained constant despite changes in junctional protein distribution on elongated cells (Figure R4B). This means for both methods, cell 2 is equally as polarized as cell 1 despite the fact that there are more proteins covering both vertical and horizontal junctions of cell 2 as compared to cell 1 (Figure R4A). Hence, both the Fourier Series and Ratio method failed to detect changes in junctional protein distribution on elongating cells and these results contradict our definition of polarization strength.

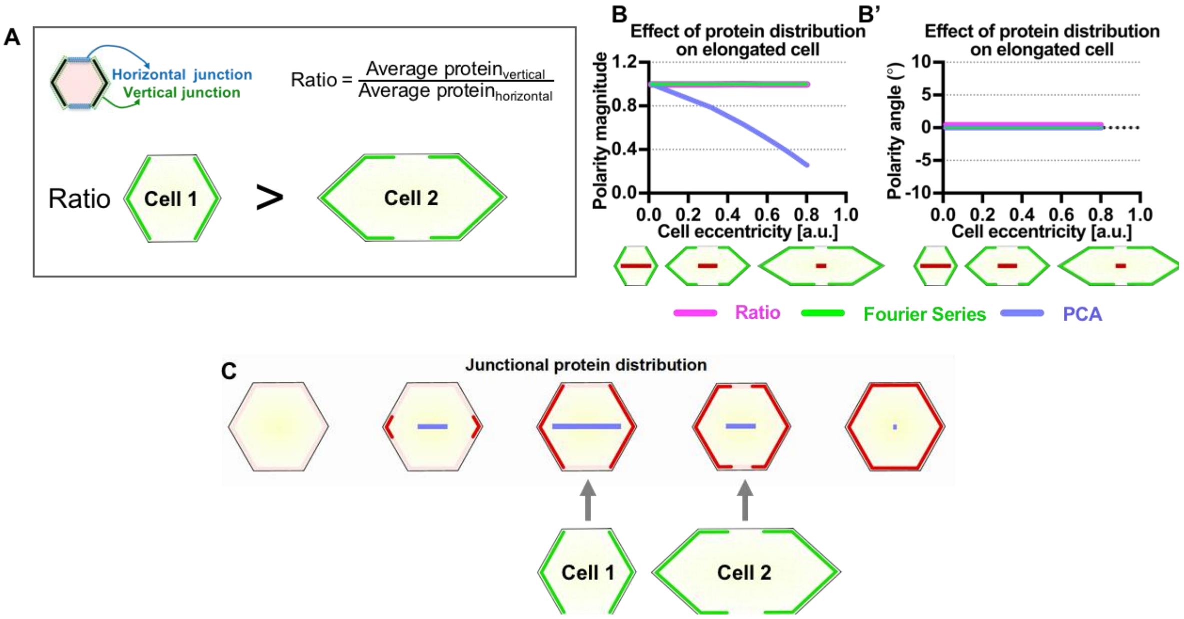

Figure R4: The Ratio and Fourier Series methods failed to detect changes in junctional protein distribution due to sensitivity to elongated cells.
(A) Polarity magnitude is determined by computing the ratio of average protein on vertical to horizontal junctions. Hence, cell 1 would exhibit higher polarity magnitude as compared to cell 2. (B-B') Quantified polarity magnitudes (B) and angles (B') of simulated cells with varying junctional peak protein distribution and cell eccentricity, from 0 to 0.8
(C) Polarity strength is determined based on junctional protein distribution, where polarity magnitude decreases when there are more proteins covering both vertical and horizontal junctions

We have now included Figure R4B into our manuscript – Figure S1F-G'. A new paragraph has been added to describe the simulation results:

Lines 180-185

We further extended these simulations by varying protein distribution while simultaneously elongating cells (Fig.S1F-G'). The PCA method successfully detected changes in protein

© 2021. Published by The Company of Biologists under the terms of the Creative Commons Attribution License (https://creativecommons.org/licenses/by/4.0/).

distribution independent of cell eccentricity. On the contrary, polarity magnitude obtained from both the Ratio and Fourier Series methods remained constant due to the sensitivity of these methods to cell eccentricity. For polarity angle, all methods remain consistently oriented at 0° or 90° (Fig.S1F'-G').

Response 2 to Comment 2

2. Subsequent PCA analysis (Eqs. 4-14):
Many of our original concerns remain. Here we focus on the two most relevant ones:
a) As we mentioned before, the polarity angle from the PCA method depends on the image brightness scaling. While the authors tested this with simulations, these simulations were carried out on perfectly mirror symmetric polarity distributions, which will of course give the expected polarity angle of zero always. This changes however as soon as the polarity distribution is not mirror symmetric any more.

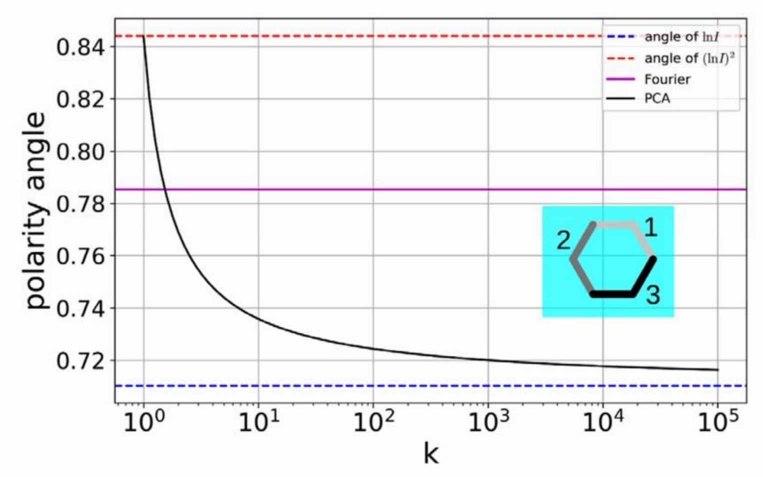

We now explicitly tested this, see angles_sketch.png, where we consider a simple toy polarity distribution as indicated by the sketch. The phenomenon we describe is generic and should appear for any distribution (of e.g. puncta) of different intensity (*more* than 2 levels) along the cell outline in a way that is *not* mirror symmetric. We find that indeed the angle obtained using the proposed PCA method depends on the brightness scaling factor (which is the same as a dependency on k in Eq. 5), and that it interpolates between the angles of the ln I and (ln I)^2 distributions. Regarding the argument of the authors in their reply: both distributions ln I and (ln I)^2 certainly have corresponding maxima and minima. However, the PCA method does not only depend on the maxima and minima, but also on any other values. This is also the reason that this effect only becomes visible for >2 different intensity levels. This stresses again the need for more realistic tests. (The puncta the authors added in their revision also seem to use just 2 different intensity levels.)

There must have been some confusion with this comment *"The puncta the authors added in their revision also seem to use just 2 different intensity levels"* as we have stated in the manuscript that for all the puncta simulations, each punctum exhibits intensity ranges from 40 to 255 a.u. One can clearly see the differences in puncta pixel intensity (represented by different grayscales) by zooming into junctional puncta intensity on simulated cell images in Figure S2B and C.

Besides that, our simulation with varying image signal-to-noise ratios also exhibit a wide range of random intensity levels) and evidently, the PCA's polarity angle is not adversely affected by the noises (consistently oriented at 0° with fluctuation of less than ±2.5°) (See Figure S2A'').

See line 883-885
Simulated cell with non-continuous junctional puncta protein distribution. Each punctum exhibits a junctional intensity profile of a Gaussian function (intensities value ranges from 40 to 255 a.u., puncta spacing of 15° and Gaussian sigma of 4.47).

© 2021. Published by The Company of Biologists under the terms of the Creative Commons Attribution License (https://creativecommons.org/licenses/by/4.0/).

Below we provide another simulation to demonstrate that polarity angle obtained from the PCA method is unaffected by intensity scaling on non-mirror asymmetry.

To demonstrate that the PCA method is indeed insensitive to intensity scaling on non-mirror asymmetry, we carried out the same exact simulation as in angles_sketch.png with varying intensity scaling factor, f (we used 'f' here to avoid confusion with our constant 'k') (Figure R5). Differently from the result shown in angles_sketch.png, our simulation results showed that the polarity angle obtained from the PCA method remained consistently oriented at -18° with maximum error of 0.15° for intensity scaling up to 10^6 (Figure R5). It is worth noting that the polarity angle fluctuation/error obtained from all methods in simulated cells due to varying cell regularity, puncta distribution, image noise and etc can go up to ±10°, while on real biological dataset (pupal wing 32 hAPF) can go up to ±30° despite being consistently oriented at 0°. Therefore, these ranges of fluctuation in polarity angle are negligible as it falls within an acceptable range (see polarity plots in Figure R5), and we concluded that the polarity angle obtained from the PCA method is unaffected by intensity scaling.

We do not understand why the Reviewer's result differs from our simulation result – where they showed the maximum error in polarity angle is ~6.87° *(based on the assumption that polarity angle from the graph provided is in degree and not radian)*. Since no details on simulation units/parameters or implementation of polarity measures script were provided, it is impossible for us to verify what has gone wrong. In any case, we have provided a set of simulated images that we used to generate these results which interested readers can easily test using our QuantifyPolarity GUI.

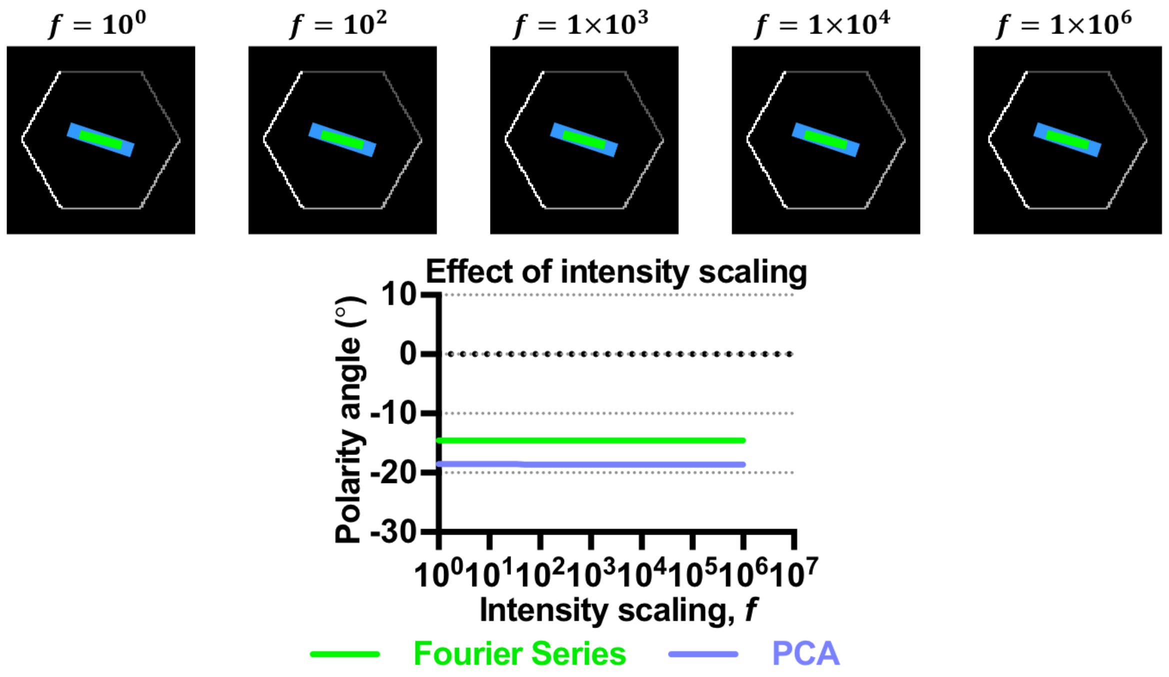

Figure R5: Effect of intensity scaling (f) on polarity angle quantification for non-mirror asymmetry.

Thus far we have provided numerous different simulations which evidently showed that the polarity angle obtained from the PCA method is unaffected by varying image intensity (see Table 2 below for different simulations on the effects of image intensity on polarity angle and related figures).

© 2021. Published by The Company of Biologists under the terms of the Creative Commons Attribution License (https://creativecommons.org/licenses/by/4.0/).

Table 2: Simulations related to the effects of image intensity on polarity angle

Simulation Description	Related Figures
Increasing peak intensity values	Figure 2F' or R8B'
Punctum with multiple intensity values on different cell geometry	Figure 2G'', 2H' and S2B''
Increasing peak and base intensity values (bipolarity)	Figure S1A''
Increasing image noises (random and multiple intensity values)	Figure S2A''
Increasing peak and base intensity values (non-mirror polarity)	Figure R5

b) It is still not clear to us in what situations the non-linearities applied in the PCA method as compared to the Fourier method provide advantages. Performance reasons brought forward by the authors are an independence of cell shape (e.g. new Fig. 2C,D), which is clearly just due to the preprocessing step. The other line of argumentation of the authors is the new Fig. 2E (old Fig 1H), i.e. the position of the peak of the polarity norm. On close inspection, we were a bit surprised by the claimed "symmetry of the polarity strength curve" of the PCA method, as this will clearly depend on the baseline and peak values in these toy examples. Indeed, our own calculations confirm this (compare to the file amplitudes.png). Moreover, while the curve of the normalized Fourier method (Eqs. 2 and 3) is indeed more skewed than the curve for the PCA method, the non-normalized Fourier method (no factor 1/N in Eqs. 2 and 3) is naturally symmetric (with only a very slight deviation due to the hexagonal cell shape), where the curve shape is independent on baseline and peak values.

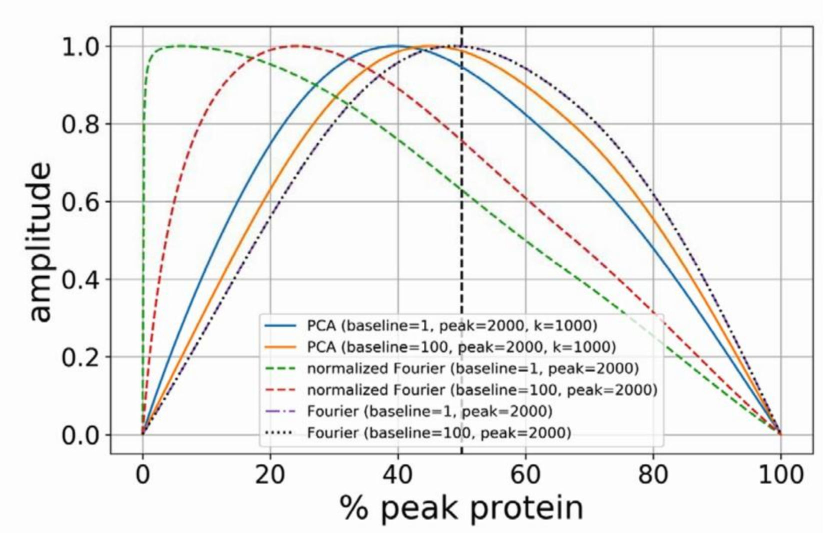

Hence, the advertised advantage of the PCA method already disappears when varying the baseline or peak values. Also, if the goal is to have a symmetric curve in this toy experiment, better methods exist already (e.g. non-normalized Fourier, *which corresponds to the PCA method without the nonlinearities*, see our previous comment 2).

Polarity magnitude from non-normalized Fourier Series is extremely sensitive to varying image intensity scaling while the PCA method is unaffected by varying intensity scaling.

In our manuscript, we have stated that the logarithmic function is crucial for intensity normalization so that the PCA readout is not affected by intensity scaling (See Line 589-590 and simulation Figure S1A). As shown in our simulation results below in Figure R6, where we increased the total junctional protein intensity by an intensity scaling factor, polarity magnitude from the non-normalized Fourier Series method increases exponentially to increasing image intensity scaling. Meanwhile, the PCA method is unaffected by varying image intensity scaling (similar to simulation in Figure S1A). We strongly believe that the intensity sensitivity of the non-normalized Fourier method is an unwanted feature from any

© 2021. Published by The Company of Biologists under the terms of the Creative Commons Attribution License (https://creativecommons.org/licenses/by/4.0/).

quantification methods, as different imaging conditions, microscope settings, and fixation protocols could substantially affect the image intensity, and hence polarity readout.

This is also precisely why Merkel et al., 2013 (the same lab that implemented the Fourier Series method in Aigouy et al., 2010) modified the original non-normalized Fourier Series method to include a normalization term so that the readout is not affected by intensities.

"This normalization step makes the polarity measure insensitive to intensity variations in images."

Merkel et al., 2014

Thus, despite possessing a symmetrical curve response, given the fact that polarity magnitude from the non-normalized Fourier Series would be distorted by images with varying intensity scaling/brightness, we would not recommend anyone to implement this method and have consequently not included it in our GUI.

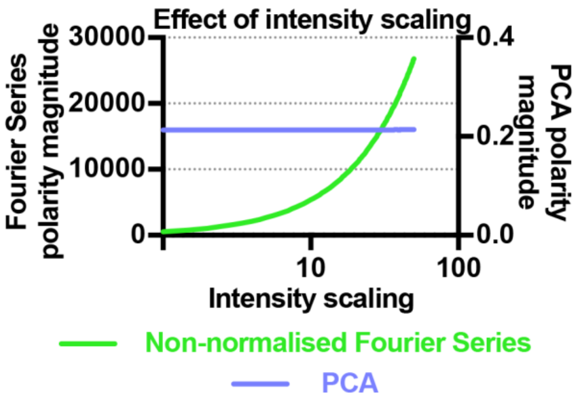

Figure R6: Polarity magnitude readout from non-normalized Fourier Series method is sensitive to varying intensity scaling.

Below we provide a simulation to show that the polarization strength profile from the normalized Fourier Series method is increasingly skewed by decreasing base intensities.

To validate the results from the Reviewer, we performed similar simulations using our QuantifyPolarity GUI (Figure R7A). In terms of PCA polarity magnitude readout, while the maximum polarity is shifted (by 10 units of peak junctional protein extent) when base:peak intensity is 1:2000 a.u. as compared to 100:2000 a.u., however the polarization strength curve remained to some degree symmetrical (Figure R7A'). In particular, for 1:2000 simulation, a cell with 20 units peak protein exhibited a comparable (weak) polarization strength as a cell with 160 units of peak protein. We have stated in the manuscript that polarity magnitude from the PCA method is decreased by 0.9% with extremely low base intensities <10 a.u., while in this example the base intensity is 1 a.u. (see Line 591-593). In line with this, we found that the symmetrical polarization strength profile from the PCA method is unaffected when base:peak intensity is 10:2000 with maximum polarity at cell with 90 units (Figure R7A'). Note that background noise due to imaging is usually above 50 a.u.

On the contrary, the normalized Fourier Series method exhibits a highly asymmetrical polarity strength profile/curve, where with base:peak intensity of 100:2000 a.u., a cell with 20 units peak protein is equally polarized as a cell with 80 units, and it attained maximum magnitude for cell with merely 50 units of peak protein (Figure R7A'). In addition to its asymmetrical curve characteristic, we also discovered that the Fourier Series method exhibits high sensitivity to extreme low base intensity, where the polarization curve would be greatly skewed when base:peak intensity is 1:2000 a.u. (Figure R7A'). In particular, the Fourier method attained maximum polarity when cell has 10 units peak protein and then continuously decreases to zero polarization, even when the units of peak protein gradually increase on both vertical junctions which corresponds to a higher degree of asymmetrical

© 2021. Published by The Company of Biologists under the terms of the Creative Commons Attribution License (https://creativecommons.org/licenses/by/4.0/).

protein localization (for example a cell with 120 units). In fact, the polarization strength profile from the Fourier Series method is skewed with decreasing base intensity, for e.g. cell with base:peak intensity of 10:2000 a.u. obtained maximum polarity at cell with 20 units.

In summary, this result demonstrates that the PCA method still exhibits to some degree a symmetrical polarization curve for extreme low base intensity (1a.u.), while the polarization strength profile from the normalized Fourier Series method is increasingly skewed by decreasing base intensities.

Hence, the PCA method is evidently more advantageous as compared to the non-normalized Fourier Series method in terms of being insensitive to intensity scaling and normalized Fourier Series method in terms of exhibiting a symmetrical polarization strength curve.

See section below for more benefits of the PCA method over the Fourier Series method.

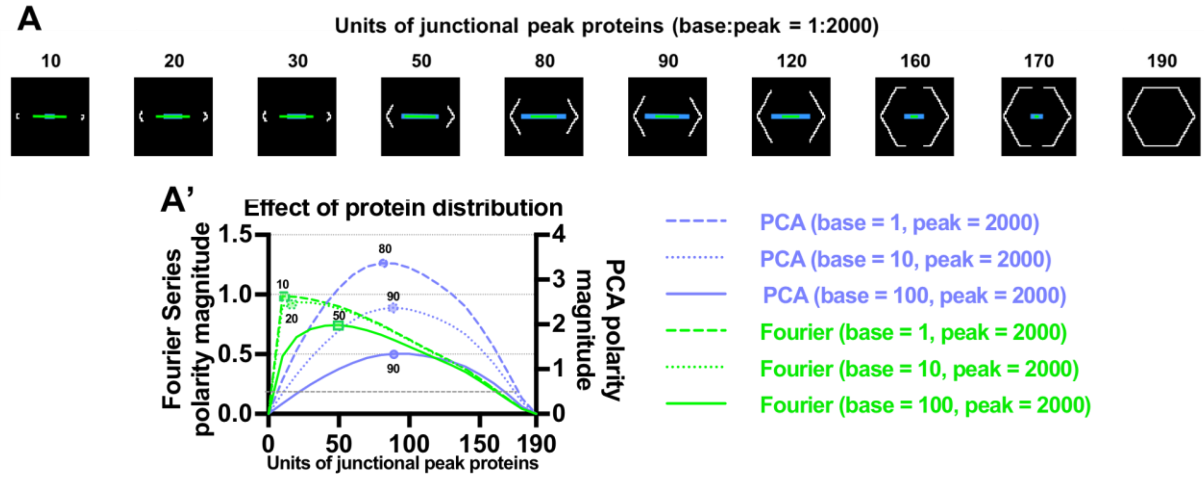

Figure R7: Effect of protein distribution on the Fourier Series and PCA method with varying base:peak intensities.
(A) Simulated cells with varying units of junctional peak proteins (base:peak is 100:2000). Length and orientation of green and blue bars denote the polarity magnitude and angle from the Fourier Series and PCA methods.
(A') Graph showing polarity magnitude obtained from both Fourier Series and PCA methods for different base:peak intensities. Blue circles and green boxes indicate maximum polarity from the PCA and Fourier Series methods respectively.

The Fourier Series method fails to detect changes in relative peak-to-base intensity ratio.

Upon further investigation into the skewness in the Fourier Series polarity strength curve, we uncovered a new insight which further showed that the PCA method exhibits another desirable property as compared to the Fourier Series method, apart from being eccentricity independent and possessing a symmetrical polarization strength profile. One of the most important criterion of a robust polarity method is the ability to detect polarization strength given variation in relative peak-to-base protein intensity (Figure 1G”). As shown in Figure R8A, given a cell with constant base protein intensity on horizontal junctions, increasing amount of peak protein on both vertical junctions should result in increasing polarity magnitude as it implies more proteins are being asymmetrically localized to the vertical junctions (see Definition of polarity strength/magnitude Line 70-77 and Figure 1G''). We performed simulation of cells with increasing peak protein intensity on vertical junctions while maintaining a constant base intensity on horizontal junctions and protein distribution to examine the polarity magnitude readout in response to increasing relative peak-to-base

© 2021. Published by The Company of Biologists under the terms of the Creative Commons Attribution License (https://creativecommons.org/licenses/by/4.0/).

intensity ratio (Figure R8A'). For example, a simulated cell with relative peak-to-base ratio of 1 indicates that the intensity values of peak and base proteins are equivalent, hence the cell is considered non-polarized (with polarity magnitude of zero) and increasing peak protein on the vertical junctions would results in increasing polarity magnitude.

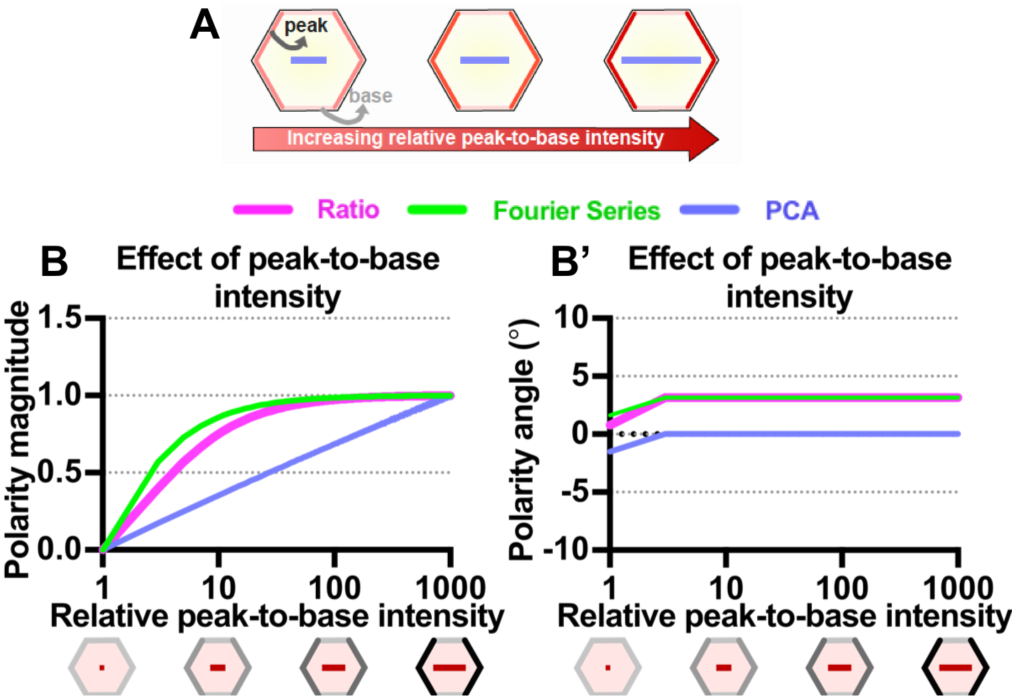

Figure R8: Effect of relative peak-to-base intensity on all polarity methods.
(A) Simulated cells with constant base protein intensity on horizontal junctions and increasing amount of peak protein on both vertical junctions. With increasing relative peak-to-base intensity, this would result in increasing polarity magnitude as more proteins are being asymmetrically localized to the vertical junctions.
(B) Graph showing polarity magnitude obtained from all polarity methods for increasing relative peak-to-base intensities.
(B') Graph showing polarity angle obtained from all polarity methods for increasing relative peak-to-base intensities.

Here, polarity magnitude is plotted against the relative peak-to-base intensity in log scale; in which a straight line indicates that when relative peak-to-base intensity is increased by a fixed percentage, the polarity magnitude increases by a corresponding fixed amount. The PCA method exhibits this characteristic for all relative peak-to-base values (Fig.2F). Unlike the PCA method, both the Ratio and Fourier Series methods only exhibit such characteristic at low relative peak-to-base ratio/values before eventually plateauing (Fig.2F). This means that the Fourier Series (and Ratio) method becomes indifferent to higher range of relative peak-to-base intensity ratio, where it fails to distinguish more polarized cells with higher peak-to-base intensity ratio from less polarized cells. Thus, the PCA method is more reliable in detecting polarization strength for a wide range of relative peak-to-base intensity values.

From this simulation result, we found that PCA method is more robust and sensitive in detecting variation in peak-to-base intensity as compared to both the Ratio and Fourier Series method. This property is essential for polarity strength detection due to increasing asymmetrical localization of protein.

We have added the following simulation along with description into our manuscript - Figure 2F-F':

Line 186-199:
An important criterion for a robust polarity method is the ability to detect different degrees of

© 2021. Published by The Company of Biologists under the terms of the Creative Commons Attribution License (https://creativecommons.org/licenses/by/4.0/).

polarization strength given variation in relative peak-to-base protein intensity (Fig.1G’’). We simulated cells with increasing peak protein intensity on vertical junctions while maintaining a constant base intensity (40 a.u.) on horizontal junctions (Fig.2F). For simulated cells with relative peak-to-base ratio intensity of 1 (equivalent peak and base intensities), the cell is non-polarized while increasing peak protein on the vertical junctions results in increasing polarity magnitude. Polarity magnitude is plotted against the relative peak-to-base intensity in log scale; in which a straight line indicates that when relative peak-to-base intensity increases by a fixed percentage, the polarity magnitude increases by a corresponding fixed amount. The PCA method exhibits this characteristic for all relative peak-to-base values (Fig.2F). Unlike the PCA method, both the Ratio and Fourier Series methods only exhibit such characteristic at low relative peak-to-base values before eventually plateauing (Fig.2F). Thus, the PCA method is more reliable in detecting polarization strength for all relative peak-to-base intensity values.

c) In addition to this, the non-linearities (Eq 5, Eq 14, our previous comment 2) of the proposed PCA method also introduce other problems, e.g. a failing of the method if only even a single pixel along the cell outline has intensity value zero, because of the logarithm in Eq. 5. More generally, the purpose of the log in Eq. 5 remains unclear. The reply to comment 3 of referee 1 in this regard is confusing - what is happening in Eq. 5 is not a normalization. Eq. 5 merely makes the measure more insensitive to the intensity values. More precisely, the log distorts the intensity values along the cell outline before computing the polarity from them. Why this distortion should be desirable is not explained. Taken together, we believe that providing the computational tool and proposing the preprocessing step as an option could be a welcome contribution to the field *if* the dependence on the definition of "eccentricity/shape independence" is better discussed. However, we think that the remaining part of the PCA method (i) amounts to being a mere modification of the Fourier method without being stated, and (ii) the modifications introduce problems into the method. The authors have tried to clarify where these modifications are useful, but as discussed above, their argumentation is not convincing. Unless it is clear in what situations and why the modifications applied to the Fourier method are useful, we cannot recommend publication.

Regarding the effect of logarithm function on intensity with 0 a.u. We have previously addressed this in Response 4 to you as follows:

See Response 4 to Comment 4 for Reviewer 2

“Intensity values I_i are extracted from standard images, $I_i \in \mathbb{N}^+$. However, as the logarithmic function is not defined when $I_i = 0$, thus the intensity value is set to 1 if it is 0, hence $I_i \in \mathbb{N}^+$. This adjustment should not affect the overall result as even for the lowest bit-depth standard image format (e.g. an 8-bit image) $1/255 \approx 0.4\%$. Besides that, there will always be stochastic fluctuation in noise (with intensity >1a.u.) due to the nature of microscope imaging.”

We have already addressed the reason for using the logarithmic function in our manuscript, responses and above. We are ourselves confused by the following statement “*The reply to comment 3 of referee 1 in this regard is confusing - what is happening in Eq. 5 is not a normalization. Eq. 5 merely makes the measure more insensitive to the intensity values.*” To be specific, we question is “making a measure insensitive to intensity value” not equivalent to “normalization”? Again we would like to reiterate that Eq. 5 is an intensity normalization function to make the PCA method insensitive to intensity scaling or brightness, a property evident in many different simulations that we have shown. Despite the Reviewer asserting that the log function in the PCA method is distorting the intensity values, no evidence has been provided to support this. Importantly, our simulation results (Figure S1A-A’’ and Figure R5) show that the PCA method is not affected by intensity scaling.

From all our simulation results, it is evident that both the Ratio and Fourier Series methods exhibit vastly different behaviors from the PCA method given different cell geometry, junctional protein distribution, relative peak-to-base intensity, image condition scenarios, quite apart from being eccentricity dependent. Hence, solving the eccentricity dependence

© 2021. Published by The Company of Biologists under the terms of the Creative Commons Attribution License (https://creativecommons.org/licenses/by/4.0/).

problem by including the cell compression step does not make either methods behave similarly to the PCA method. It is precisely because of this we have provided a detailed description of their behaviors given certain scenarios to cater for a wide variation in polarization systems. We believe that in this way the GUI is beneficial to serve a wide variety of different polarization systems, not just limited to *Drosophila* epithelial tissues.

We disagree with the claim that the PCA method is a mere modification of the Fourier method as it clearly behaves differently from the Fourier Series as well as the non-normalized Fourier (and this would be so even with the cell compression step), as shown in our simulation results. We note that even with cell compression step, the Fourier Series method will never behave similar to the PCA method and will still inevitably suffer from several other limitations, in particular failure to detect different degrees of polarization strength due to varying relative peak-to-base protein intensity and exhibiting an asymmetrical polarization strength curve that can be further skewed by decreasing intensity. Please see Table 1 for the performance comparison between the PCA and Fourier Series methods.

Secondly, in the eye of the Reviewer, it seems that the most “controversial” modification that we have implemented in the PCA method is the introduction of a logarithmic term, which is necessary for intensity normalization. However, the Reviewer suspected that this will result in polarity angle from the PCA method being sensitive to image intensity scaling, without any substantial evidence for this. To prove this, we have simulated various scenarios with varying intensity scaling (see Table 2) and found no evidence that the polarity angle output from PCA method can be affected by varying intensity scaling. In fact, the PCA method outperformed the Fourier Series method in all simulations, suggesting that the modifications to the PCA method are in fact beneficial for planar polarity quantification.

Response 3 to Minor points

Minor points:
-We had serious trouble running the tool on windows, we found a solution here: https://www.mathworks.com/matlabcentral/answers/98050-why-do-i-get-an-error-sayingundefined- function-or-variable-matlabrc-when-executing-a-program-th
we think the authors should either fix their tool or add this link to the README of their github page, so that users can find it.
-We think that in order for reviewers to evaluate a software, they should be given access to the code of the tool, we note that for the second time we were not given access to it.

We appreciate the Reviewer taking the trouble to use our QuantifyPolarity GUI. In fact, the GUI has been widely used by our research group members (>10 people) on computers with varying operating systems (Windows and macOS), however we have not come across any errors. We are happy to receive any feedback for further improvement of this GUI – indeed if the Reviewer had been unable to get the GUI to correctly function, we would have been happy for them to send detailed information via the editorial staff so that we could assist them. It would be helpful if Reviewer would list out the encountered problems for us to troubleshoot.

As mentioned in the responses, we will make QuantifyPolarity open source once published, to allow qualified users to adapt and improve as they wish. (We note that in comparison other commonly used GUIs such as PackingAnalyzer and the newer version TissueAnalyzer are not open source.)

Unlike others script-based software, the QuantifyPolarity is an interactive and user-friendly GUI where users can easily and automatically evaluate the GUI by running a batch of images on it. Hence, we have included images of simulated cells for various scenarios for evaluating the performance of our GUI. We strongly advise using our GUI to be consistent with our reported simulation results.

© 2021. Published by The Company of Biologists under the terms of the Creative Commons Attribution License (https://creativecommons.org/licenses/by/4.0/).

Third decision letter

MS ID#: DEVELOP/2020/198952

MS TITLE: QuantifyPolarity, a new tool-kit for measuring planar polarized protein distributions and cell properties in developing tissues

AUTHORS: Su Ee Tan, Weijie Tan, Katherine H Fisher, and David Strutt

I have now received the review from reviewer 2.
While other reviewers support publication, there appears some persistent disagreement which may be hard to resolve in full. I suggest that to move forward, you discuss openly the issues and in particular you discuss the potential limits of the approach proposed and the concept of shape-independence as detailed under their point 3. It is important that readers understand the value of different approaches and may use both QuantifyPolairty and Tissue Analyser and compare results. The comparison of both approaches should prove very useful to the community. The manuscript will not be sent back to this reviewer but I will look at the response myself.

Reviewer 2

Advance summary and potential significance to field

We will stop reviewing this manuscript, as we feel there is no convergence between the authors and us on the main points.

There are many comments to make, but we are unfortunately constrained by time.
Here we focus just on what we regard as the major problems.

Comments for the author

We will stop reviewing this manuscript, as we feel there is no convergence between the authors and us on the main points.

There are many comments to make, but we are unfortunately constrained by time. Here we focus just on what we regard as the major problems.
1. The brightness dependence of the PCA polarity angle is a fundamental problem that prevents us fro recommending publication. We now attach our python code used to show this and its output (see th angles_PCA.py code at the end of our review and Fig. R1; up to an angle flip same output as before). I their simulation, the authors don't indicate the values of the intensity levels (they are possibly not 1, and 3 as in our example).

© 2021. Published by The Company of Biologists under the terms of the Creative Commons Attribution License (https://creativecommons.org/licenses/by/4.0/).

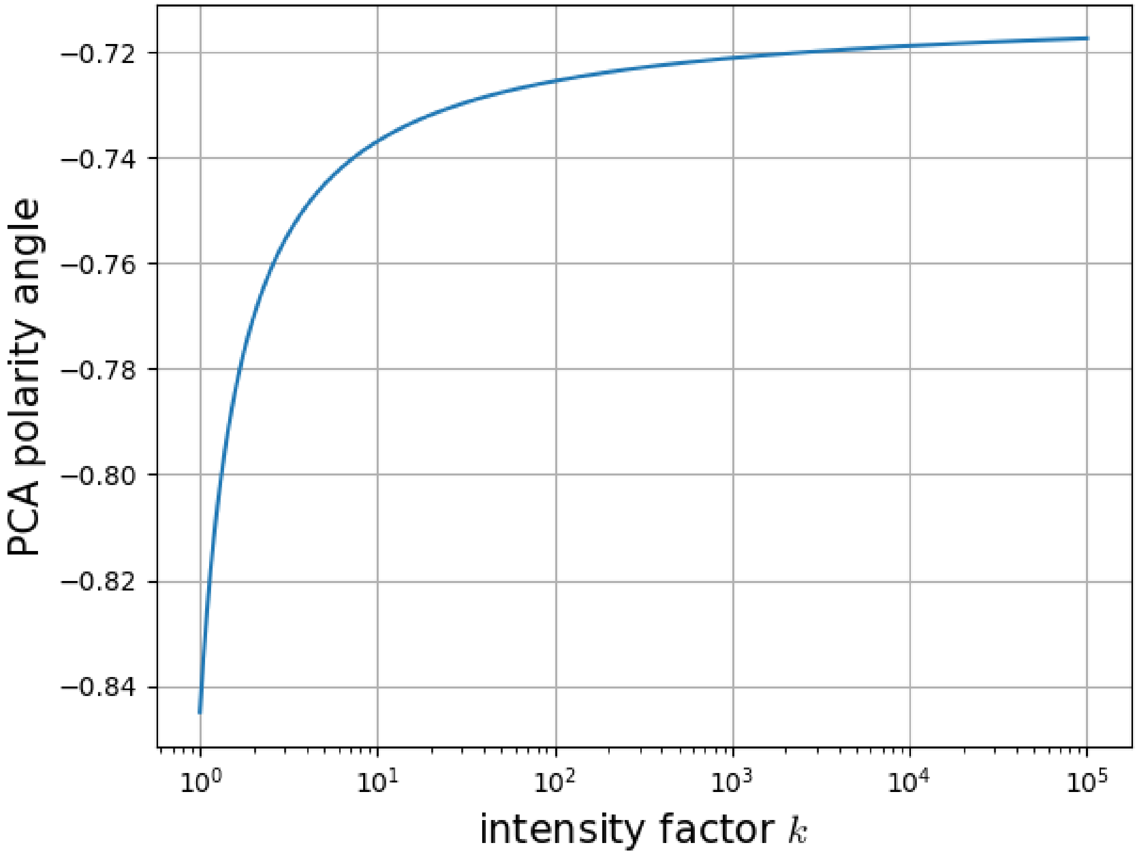

Fig. R1 Brightness dependence of the PCA polarity angle

We stress again that this brightness dependence can also be clarified analytically (see previous round of review). The authors' maximum-intensity argument is not sufficient to refute our analytical argument as the whole intensity distribution counts, and the distributions (ln I) and (ln I)^2 are different and will have different angles (see previous round of review).

While this angle dependency can be decreased by increasing the overall intensity (and k), it makes the method more brittle (i.e., the user needs to make sure in which parameter range she/he is). Moreover, we just took a simple toy example, and so far, nobody knows how strong the angle deviations can become. In any case, we feel that the authors should be up-front to the reader about this property of their method.

2. Moreover, recent tests that we carried out with the authors' software revealed problems, where polarity appears wrongly quantified for simple examples (e.g., see Fig. R2). The output of the authors' software is shown in blue (PCA) and green (Fourier). We compare to the Tissue Analyzer implementation of the Fourier polarity (yellow). As the sample cell is symmetric across the edge with increased intensity, the quantified polarity should point right to its middle, which is not the case for the output of the authors' software.

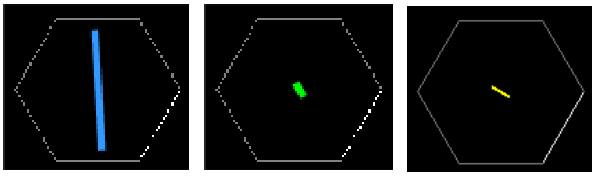

Fig. R2

© 2021. Published by The Company of Biologists under the terms of the Creative Commons Attribution License (https://creativecommons.org/licenses/by/4.0/).

PCA with compression/Quantifypolarity Fourier implementation/Original Fourier implementation

3. Regarding the compression step, we reiterate the point that we made since the first round of review: Whether a given method is independent of geometry depends on the definition of "independent of geometry". While in their last revision, the authors said more about their definition of geometry independence, it should be still made very clear that there are many reasonable definitions of shape independence, and so there is no "best" method, as this depends on the needs of the user.

We maintain that a change in total protein amounts as created by the authors' compression step will be surprising to a substantial fraction of users. In this context, check marks as in table 1, or overly general claims are profoundly misleading. We propose instead to say in which sense the respective method is shape independent, as this allows the user to decide.

We stress again that if not correctly discussed, the effect of the compression step can lead to unexpected biases of the polarity by cell eccentricity. E.g., if a user expects that sum intensity matters then using the average-intensity-based compression step will lead to a polarity that is biased, tending to be oriented parallel to cell eccentricity (because interfaces aligned with the stretch axis will count less in the final polarity, see Fig. R3).

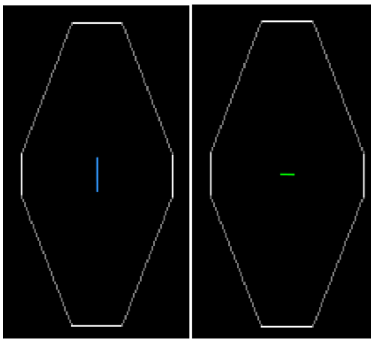

Fig. R3 Compression applied prior to running the PCA method aligns polarity angle with cell eccentricity PCA with compression/Quantifypolarity Fourier implementation

While the authors use an average-intensity-based paradigm to justify their compression step, a total-intensity-counts paradigm could also be reasonable. This is because it asserts that actual protein amounts induce signalling, while the length of planar-polarity-protein-free membrane is potentially less important. However, until we fully know how the respective planar polarity systems work on a precise quantitative level, nobody can know what the "right" definition of e.g. shape independence is. Thus, the need to be open here and transparent towards the user.

This issue is also closely linked to the kinds of tests the authors carry out to benchmark their method. For example, the eccentricity simulation by the authors in Fig. 2C assumes that during cell deformation, the average protein concentration on a stretched interface remains constant, instead of the total protein amount. This would mean that protein binding/unbinding needs to be coupled to cell geometry change in a very specific way. This very specific assumption is probably not true in most cases. However, the authors use this as a criterion to judge about shape independence and distribute check marks in table 1, which we find highly problematic.

Note that there is also a definition for shape independence for which even the Fourier method (and equally the PCA method without compression step) are shape independent (i.e. keeping the angular intensity distribution constant during the cell deformation).

4. We also note that Table 1 appears biased towards the PCA method quite generally. This does not only apply to the questions related to cell eccentricity (see previous point). For example, for 'Varying relative peak-to-base intensity' the authors give only their PCA method a check mark even though all three methods show varying polarity magnitude. Just the specific non-linear way in which the magnitude depends on the peak-to-base ratio differs between the methods (with their

© 2021. Published by The Company of Biologists under the terms of the Creative Commons Attribution License (https://creativecommons.org/licenses/by/4.0/).

method it varies logarithmically, meaning it too becomes less sensitive with increasing ratio). Generally, checks should be replaced by an unbiased description explaining in which sense the respective method is perturbation independent, as this allows the user to decide.

Python code of 'angles_PCA.py' used to illustrate the brightness dependence of the PCA polarity angle

```
from numpy import *

from pylab import *


N = 3000


# intensity distribution

dtheta = 2*pi/N

theta = linspace(0, 2*pi-dtheta, N).reshape(1, -1)

I = where(theta<2*pi/3, 1.0, where(theta<4*pi/3, 2.0, 3.0))


# PCA method

w = dtheta

k = 10**linspace(0, 5, 251).reshape(-1, 1)

I_hat = log(k*I)

x_hat = I_hat*cos(theta)

y_hat = I_hat*sin(theta)


w_sum = w*N

sigma_xx = sum(w*x_hat**2, axis=1)/w_sum

sigma_xy = sum(w*x_hat*y_hat, axis=1)/w_sum

sigma_yy = sum(w*y_hat**2, axis=1)/w_sum


angle = 0.5*arctan2(2*sigma_xy, sigma_xx-sigma_yy)


#plot

subplots_adjust(left=0.14, bottom=0.13, top=0.95, right=0.95)

plot(k, angle)

xlabel("intensity factor $k$", fontsize=15)

xscale("log")

ylabel("PCA polarity angle", fontsize=15)

grid()

savefig("angle.png")
```

© 2021. Published by The Company of Biologists under the terms of the Creative Commons Attribution License (https://creativecommons.org/licenses/by/4.0/).

Third revision

Author response to reviewers' comments

Response 1 to Comment 1

The brightness dependence of the PCA polarity angle is a fundamental problem that prevents us from recommending publication. We now attach our python code used to show this and its output (see the angles_PCA.py code at the end of our review and Fig. R1; up to an angle flip same output as before). In their simulation, the authors don't indicate the values of the intensity levels (they are possibly not 1, 2 and 3 as in our example). We stress again that this brightness dependence can also be clarified analytically (see previous round of review). The authors' maximum-intensity argument is not sufficient to refute our analytical argument as the whole intensity distribution counts, and the distributions (ln I) and (ln I)^2 are different and will have different angles (see previous round of review). While this angle dependency can be decreased by increasing the overall intensity (and k), it makes the method more brittle (i.e., the user needs to make sure in which parameter range she/he is). Moreover, we just took a simple toy example, and so far, nobody knows how strong the angle deviations can become. In any case, we feel that the authors should be up-front to the reader about this property of their method.

Regarding the discrepancy between the reviewer's analysis and ours, we have found a mistake in the python code that Reviewer provided. One obvious reason why their implementation gives different outputs as compared to our QuantifyPolarity method is because factor k is missing from their code implementation (See Eqn 8 in our manuscript). Note that in their simulation in Figure R1, they used k as intensity factor/scaling of a cell, ranging from 1 to 10^5 a.u, to test the sensitivity of polarity angle to different level of image intensity scaling. This should not be confused with our factor k as a normalization constant (where k = 1000) for the PCA method (See Line 596-598).

If factor k is properly implemented in their code as shown below, then one would find that polarity angle output from PCA method is insensitive to image brightness. Corrected implementation should be as follows:

Original Reviewer's code: I_hat = log(k*I)
Corrected code: I_hat = log(1000*k*I)
Note that 1000 here represents our normalization constant k (See Eqn 8 in our manuscript)

See Figure R5 from previous response where we have shown that the PCA method remained consistent with an extremely low fluctuation at ±0.15° for intensity scaling ranges from 1 a.u. to 10^6 a.u. (Figure R5). Note that this range of intensity in our simulation, which is greater than their toy problem, covers all possible ranges of image intensity (the max intensity for a 16-bit image is only 65536 a.u.). Even for such an extreme range of intensity scaling, we have shown that the polarity angle fluctuation is negligible at ±0.15°. Thus, we conclude that the polarity angle obtained from the PCA method is not significantly affected by intensity scaling.

Response 2 to Comment 2

Moreover, recent tests that we carried out with the authors' software revealed problems, where polarity appears wrongly quantified for simple examples (e.g., see Fig. R2). The output of the authors' software is shown in blue (PCA) and green (Fourier). We compare to the Tissue Analyzer implementation of the Fourier polarity (yellow). As the sample cell is symmetric across the edge with increased intensity, the quantified polarity should point right to its middle, which is not the case for the output of the authors' software.

Regarding the discrepancy on Fourier Series polarity outputs from the QuantifyPolarity and TissueAnalyzer sofware, we are somewhat puzzled as looking at the results provided by the Reviewer they appear to be using different simulated images to do the comparison and so it's not surprising the results are different. On close inspection, both the left and middle images

© 2021. Published by The Company of Biologists under the terms of the Creative Commons Attribution License (https://creativecommons.org/licenses/by/4.0/).

differ from the right image in terms of the protein intensity discontinuity on the cell boundary (although possibly this is something to do with the way the figure was made?).

To clarify the issue, we simulated a unipolarized cell and analyzed it using both tools. As shown in the Figure R1(A) below, the polarity angle from the PCA method gives the expected output, similar to the polarity angle obtained from the Fourier Series method from both QuantifyPolarity and TissueAnalyzer. Moreover, we found that polarity angles obtained from the Fourier Series method in both software packages are indeed similar (we have included this simulated cell for verification if necessary - link is available in our github page).

Besides that, we analyzed simulated cells with varying eccentricity using TissueAnalyzer and compared it to the Fourier Series method in QuantifyPolarity. The results obtained from the QuantifyPolarity and TissueAnalyzer software are similar (albeit with very slight differences in polarity readout) (Figure R1(B)). As expected, TissueAnalyzer also suffers similar shape-dependency as seen for the Fourier method implemented in QuantifyPolarity. The small differences in polarity readout are possibly due to the image preprocessing step. One of the main distinctions between the two software packages is the differences in binary segmented masks connectivity between QuantifyPolarity (4-connectivity) and TissueAnalyzer (8-connectivity), which would result in slight deviations in polarity readout on these binarized simulated cells. All mathematical formulations for the Fourier Series method are implemented as in the published studies (Aigouy et al., 2010; Merkel et al., 2016). Due to TissueAnalyzer not being open source, it is impossible for us to find out the real answer. Overall, we have shown that Fourier Series methods implemented in both tools are similar (although not 100% possibly due to differences in preprocessing) and exhibit similar sensitivity to cell eccentricity on identical set of images. We believe our comparison is unbiased and objective as we accessed all polarity methods using the same simulated cells.

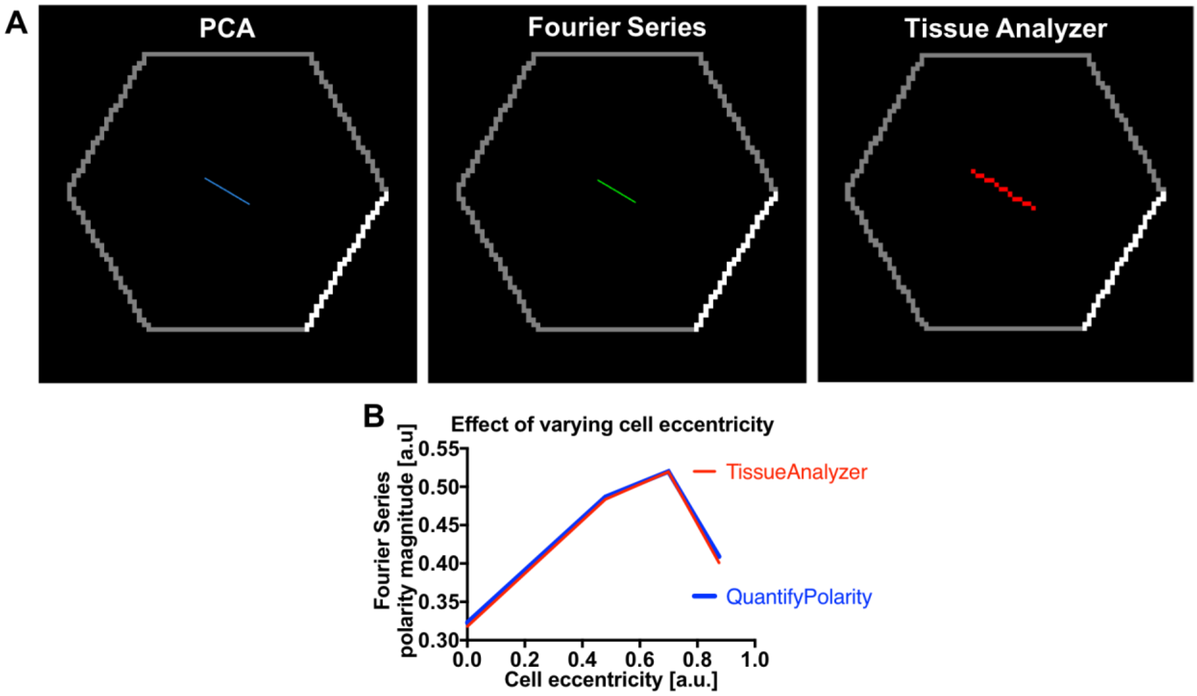

Figure R1: A direct comparison between QuantifyPolarity and TissueAnalyzer.
(A) Simulation results of a unipolarized cell obtained from QuantifyPolarity (right and middle) and TissueAnalyzer (left). Polarity angles obtained from all methods are similar.
(B) Polarity magnitude obtained from the Fourier Series method output from QuantifyPolarity and TissueAnalyzer software with varying cell eccentricity. Polarity readout from the Fourier Series method in TissueAnalyzer is sensitive to cell eccentricity, similar to QuantifyPolarity.

Additionally, we would like to point out that the examples of simulated cells provided by the Reviewer (in Comment 1 and 2) are unipolarized rather than bipolarized – where protein is localized on only on side of the junction. While we have not done extensive simulations on

© 2021. Published by The Company of Biologists under the terms of the Creative Commons Attribution License (https://creativecommons.org/licenses/by/4.0/).

the behaviour of all the methods for unipolarization cases, however mathematically we think that all polarity methods described in this paper are only useful for bipolarization quantification. For unipolarization quantification, one could possibly attempt to use the method as described in Tetley et al., 2016, which uses the first component of Fourier Series as a unipolarity measure. However, the described method is also sensitive to cell elongation and possibly exhibits similar weaknesses to the Fourier Series method.

While we did mention that polarity magnitude computed using the PCA method measures bipolarization in our manuscript (Line 607), to make it clearer, we have added the following in our manuscript:

Line 78-82
Note that although in principle unipolarity could also be measured (asymmetric localization of a protein to one side of a cell), within an epithelium the tight apposition of neighboring cell junctions generally makes it impossible to distinguish a unipolarized distribution from a bipolarized distribution. In this work, we consider methods designed to measure bipolarity.

Response 3 to Comment 3

Regarding the compression step, we reiterate the point that we made since the first round of review: Whether a given method is independent of geometry depends on the definition of "independent of geometry". While in their last revision, the authors said more about their definition of geometry independence, it should be still made very clear that there are many reasonable definitions of shape independence, and so there is no "best" method, as this depends on the needs of the user. We maintain that a change in total protein amounts as created by the authors' compression step will be surprising to a substantial fraction of users. In this context, check marks as in table 1, or overly general claims are profoundly misleading. We propose instead to say in which sense the respective method is shape independent, as this allows the user to decide. We stress again that if not correctly discussed, the effect of the compression step can lead to unexpected biases of the polarity by cell eccentricity. E.g., if a user expects that sum intensity matters then using the average-intensity-based compression step will lead to a polarity that is biased, tending to be oriented parallel to cell eccentricity (because interfaces aligned with the stretch axis will count less in the final polarity, see Fig. R3). While the authors use an average-intensity-based paradigm to justify their compression step, a total intensity-counts paradigm could also be reasonable. This is because it asserts that actual protein amounts induce signalling, while the length of planar-polarity-protein-free membrane is potentially less important. However, until we fully know how the respective planar polarity systems work on a precise quantitative level, nobody can know what the "right" definition of e.g. shape independence is. Thus, the need to be open here and transparent towards the user.

We agree with the reviewer that there are myriad possible definitions of planar polarization and it is important that the user understands the definition being used before embarking on any analysis.

For what it is worth, the current definition of planar polarization in our paper is based on many well-published studies for various systems over the decade.

Specifically, in our manuscript, we have been very careful to make it clear how we define planar polarization and what properties a useful polarity measurement method should possess. We have also clearly stated the reasoning behind our method of polarity quantification, which is adopted from the ratio method (based on previous published works) so that the polarity readout would essentially be shape independent:

Line 84-90
The definition of cell shape independence is adopted from the ratio method of quantifying polarity, where polarity magnitude is computed as the ratio of average protein on opposite junctions, rendering this method independent of junction length and hence cell elongation. If instead total protein on a cell junction is considered, even with a homogeneous protein distribution, more elongated cells will appear more polarized than less elongated ones

© 2021. Published by The Company of Biologists under the terms of the Creative Commons Attribution License (https://creativecommons.org/licenses/by/4.0/).

simply because longer junctions have higher total protein.

As shown in the example in Fig.R3 that Reviewer 2 provided, the PCA method performs correctly in quantifying polarity – where polarity angle aligns parallel to cell elongation as there is indeed higher average protein on horizontal junctions than vertical junctions. This is consistent with our definition of polarity which is based on the *average* junctional intensity.

Reviewer 2 argues that the PCA method would results in biased results if the user expects that *total* intensity matters more than *average* intensity.

Allow us to highlight two major reasons why polarization is better defined based on *average* junctional intensity, rather than *total* intensity:

1) **We emphasize that in our view, the fundamental definition of polarization is having protein asymmetrically segregated to opposite side of the cell junctions.** This also means that a cell should not be polarized when protein is homogenously distributed on all cell junctions. One highly problematic issue arises if *total* protein is considered, as an elongated cell with homogenously distributed junctional protein would result in a strong polarity magnitude even though this cell should be non-polarized (See Figure R2 below for polarity magnitude of Cell C and Cell D where ratio is 2 and 3 respectively). **Hence, while there might be other potential definitions of polarization, we argue that considering *total* intensity contradicts the fundamental definition of polarization.**

2) By taking *average* junctional intensity, the polarity magnitude isn't affected by cell elongation and therefore is shape independent. For example, even if these simulated cells are further stretched/elongated (which is not demonstrated in their example), polarity magnitude would not be affected such that more elongated cells wouldn't appear to be more polarized simply because longer junctions have higher total protein. **Hence, polarity definition based on *average* intensity is crucial for being shape independence**.

Once again, this has been discussed in our previous response to reviewers - whether *total* intensity or *average* intensity matters, and also in the manuscript (see above).

Below we have copied our previous explanation (in blue text) for convenience:

In Figure R2 from previous response, we provided another simple example to illustrate the importance of considering the *average* protein rather than *total* amount of proteins per junction.

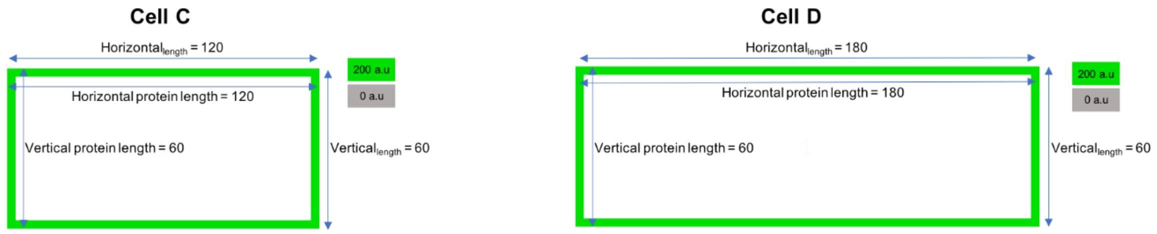

Cell C

$\text{Total protein}_{horizontal}$ = Protein intensity x Horizontal protein length = 200 a.u. x 120 = 24000

$\text{Total protein}_{vertical}$ = Protein intensity x Vertical protein length = 200 a.u. x 60 = 12000

$$\text{Ratio} = \frac{\text{Total protein}_{horizontal}}{\text{Total protein}_{vertical}} = \frac{24000}{12000} = 2$$

$\text{Average protein}_{horizontal}$ = Total protein intensity/$\text{Horizontal}_{length}$ = 24000/120 = 200

$\text{Average protein}_{vertical}$ = Total protein intensity/Vertical_{length} = 12000/60 = 200

$$\text{Ratio} = \frac{\text{Average protein}_{horizontal}}{\text{Average protein}_{vertical}} = \frac{200}{200} = 1$$

Cell D

$\text{Total protein}_{horizontal}$ = Protein intensity x Horizontal protein length = 200 a.u. x 180 = 36000

$\text{Total protein}_{vertical}$ = Protein intensity x Vertical protein length = 200 a.u. x 60 = 12000

$$\text{Ratio} = \frac{\text{Total protein}_{horizontal}}{\text{Total protein}_{vertical}} = \frac{36000}{12000} = 3$$

$\text{Average protein}_{horizontal}$ = Total protein intensity/$\text{Horizontal}_{length}$ = 36000/180 = 200

$\text{Average protein}_{vertical}$ = Total protein intensity/Vertical_{length} = 12000/60 = 200

$$\text{Ratio} = \frac{\text{Average protein}_{horizontal}}{\text{Average protein}_{vertical}} = \frac{200}{200} = 1$$

© 2021. Published by The Company of Biologists under the terms of the Creative Commons Attribution License (https://creativecommons.org/licenses/by/4.0/).

Figure R2: Example of simulated cells with homogeneous protein level on all junctions where cell D is more elongated than cell C.

As defined in the manuscript, when proteins are homogenously distributed to all junctions, cells should be non-polarized (with ratio of 1). In these simulated cells, it is evident that by computing ratio of *total* protein on horizontal to vertical junctions, cells C and D exhibit polarity readouts of 2 and 3 respectively which imply that these cells are actually polarized even with homogenous protein distribution (Figure R2). In fact, cell D exhibits higher polarity than cell C simply because there is more total protein on its horizontal junctions (due to longer junction length) as compared to cell C. On the contrary, by taking the ratio of *average* protein on horizontal to vertical junctions, both cells C and D equally exhibit a ratio of 1 which corresponds to a non-polarized state.

In short, we would like to emphasize that polarity magnitude is determined based on *average* junctional protein and not *total* junctional protein. By considering *total* amount of protein, the measure becomes inherently shape dependent, as even for a homogenous protein distribution, longer junctions exhibit more total protein and vice versa.

We note that if a user insists on a polarity measurement that considers *total* cell intensity rather than *average* intensity, **unfortunately none of the methods would give the desired results** (e.g. see previous response Figure R3 where the Fourier Series method's polarity magnitude drops despite continuous increment of total proteins).

In summary, we believe this issue has already been addressed in the manuscript and (more extensively) in the previous response to reviewers. In particularly, we are explicit with regard to defining planar polarization on the basis of *average* junctional intensities.

This issue is also closely linked to the kinds of tests the authors carry out to benchmark their method. For example, the eccentricity simulation by the authors in Fig. 2C assumes that during cell deformation, the average protein concentration on a stretched interface remains constant, instead of the total protein amount. This would mean that protein binding/unbinding needs to be coupled to cell geometry change in a very specific way. This very specific assumption is probably not true in most cases. However, the authors use this as a criterion to judge about shape independence and distribute check marks in table 1, which we find highly problematic. Note that there is also a definition for shape independence for which even the Fourier method (and equally the PCA method without compression step) are shape independent (i.e. keeping the angular intensity distribution constant during the cell deformation).

The purpose of simulation in Figure 2C is to demonstrate that cells with similar average protein but different cell elongation should exhibit similar polarity magnitude. Similar to the concept explained in Figure R2 above, where both Cell C and D with **equal average protein** should exhibit **similar polarity magnitude** despite **differences in cell elongation**. We believe that this simulation is essential to illustrate both the fundamental definition of polarization and shape independence properties of a polarity method.

We are unsure if the Reviewer missed one of the simulations where we provided and discussed simulated cells with different elongation while maintaining total amount of protein (to test different assumptions). In fact, we have already provided an in-depth response to this comment regarding keeping total protein constant during cell elongation and we have also discussed this simulation in our manuscript (see Fig.S1F-G' and Lines 185-190). For convenience, we have copied and pasted the entire previous response (in blue text) below:

Below we provided another simulation to show that the sensitivity of both Ratio and Fourier Series methods to cell eccentricity can result in failure to detect changes in junctional protein distribution on elongated cells:

We think the Reviewer is arguing that by conserving total amount of protein while elongating a cell, both Fourier Series and Ratio methods should result in "eccentricity independent" behavior. However, this seems to overlook the fact that junctional protein distribution is also

© 2021. Published by The Company of Biologists under the terms of the Creative Commons Attribution License (https://creativecommons.org/licenses/by/4.0/).

changing/varying during cell elongation. Our new simulation (Figure R4) resembles the simulation in Figure 2E, except that now junctional protein distribution varies on an elongating cell rather than on a regular cell as in Figure 2E. A good polarity method should be able to detect changes in junctional protein distribution independently of cell elongation. While both the Fourier Series and Ratio methods can detect changes in junctional protein distribution on a regular cell (see Figure 2E), but evidently these methods failed to detect changes in junctional protein distribution on cells that are at the same time changing their elongation, due to inherent sensitivity to cell eccentricity of these methods.

For simplicity, let us use the same concept from the ratio method explained above on these simulated cells (Figure R4A). If we compute the ratio of *average* protein on vertical to horizontal junctions on these cells, cell 1 would exhibit much higher ratio or polarity magnitude than cell 2 (Figure R4A). This is in line with our definition of polarity strength based on junctional protein distribution, where polarity magnitude decreases when there are more proteins covering both vertical and horizontal junctions as shown in Figure R4C (see Definition of Polarity Magnitude in Line 84-90 and Figure 1G and Figure 2E).

On the contrary, polarity magnitude obtained from both the Ratio and Fourier Series methods remained constant despite changes in junctional protein distribution on elongated cells (Figure R4B). This means for both methods, cell 2 is equally as polarized as cell 1 despite the fact that there are more proteins covering both vertical and horizontal junctions of cell 2 as compared to cell 1 (Figure R4A). Hence, both the Fourier Series and Ratio method failed to detect changes in junctional protein distribution on elongating cells and these results contradict our definition of polarization strength.

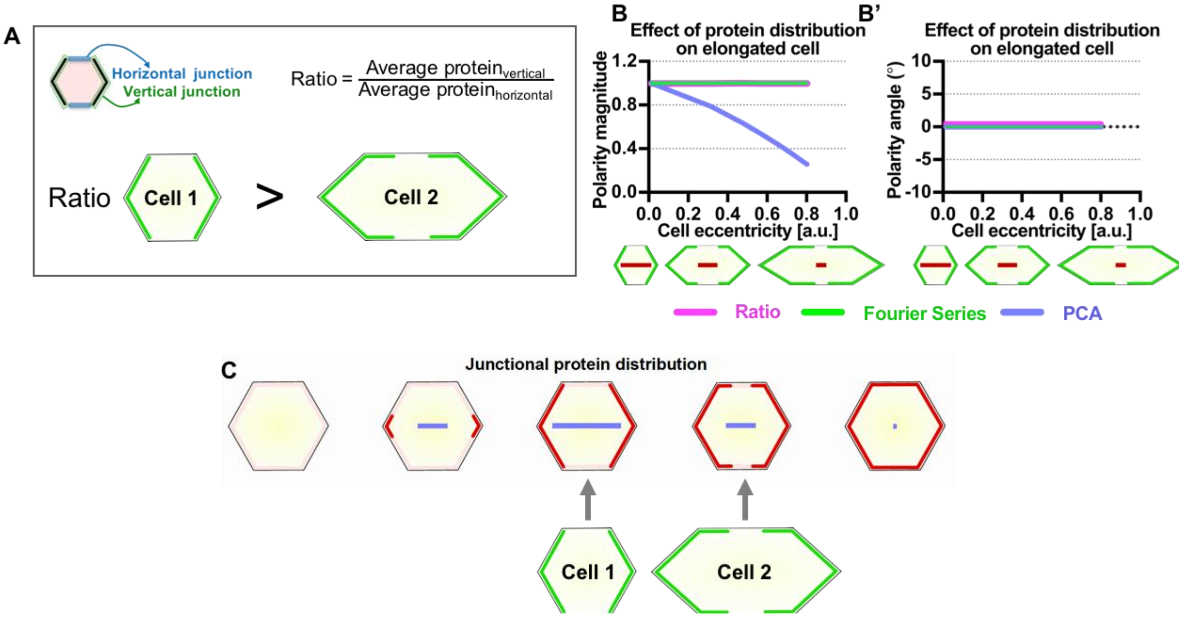

Figure R4: The Ratio and Fourier Series methods failed to detect changes in junctional protein distribution due to sensitivity to elongated cells.
(A) Polarity magnitude is determined by computing the ratio of average protein on vertical to horizontal junctions. Hence, cell 1 would exhibit higher polarity magnitude as compared to cell 2.
(B-B') Quantified polarity magnitudes (B) and angles (B') of simulated cells with varying junctional peak protein distribution and cell eccentricity, from 0 to 0.8.
(C) Polarity strength is determined based on junctional protein distribution, where polarity magnitude decreases when there are more proteins covering both vertical and horizontal junctions.

We have now included Figure R4B into our manuscript – Figure S1F-G'. A new paragraph has been added to describe the simulation results:

© 2021. Published by The Company of Biologists under the terms of the Creative Commons Attribution License (https://creativecommons.org/licenses/by/4.0/).

Line 185-190

We further extended these simulations by varying protein distribution while simultaneously elongating cells (Fig.S1F-G'). The PCA method successfully detected changes in protein distribution independent of cell eccentricity. On the contrary, polarity magnitude obtained from both the Ratio and Fourier Series methods remained constant due to the sensitivity of these methods to cell eccentricity. For polarity angle, all methods remain consistently oriented at 0° or 90° (Fig.S1F'-G').

Response 4 to Comment 4

We also note that Table 1 appears biased towards the PCA method quite generally. This does not only apply to the questions related to cell eccentricity (see previous point). For example, for 'Varying relative peak-to-base intensity' the authors give only their PCA method a check mark even though all three methods show varying polarity magnitude. Just the specific non-linear way in which the magnitude depends on the peak-to-base ratio differs between the methods (with their method it varies logarithmically, meaning it too becomes less sensitive with increasing ratio). Generally, checks should be replaced by an unbiased description explaining in which sense the respective method is perturbation independent, as this allows the user to decide.

We can see where the reviewer is coming from here. In general, we believe we have made a clear description on this matter in our manuscript, but the tick in the table may seem misleading (See Line 197-200):
Polarity magnitude is plotted against the relative peak-to-base intensity in log scale; in which a straight line indicates that when relative peak-to-base intensity increases by a fixed percentage, the polarity magnitude increases by a corresponding fixed amount

Hence the way to interpret a straight line in this graph is that given a fixed percentage increment in relative peak-to-base intensity ratio, the polarity magnitude would increase by a corresponding fixed amount – this is true for the PCA method for all increasing peak-to-base ratio values.
We have replaced it with ✓* and provide a fuller description in the table legend.
Line 983-984
✓* indicates when relative peak-to-base intensity increases by a fixed percentage, the polarity magnitude increases by a corresponding fixed amount.

<u>Fourth decision letter</u>

MS ID#: DEVELOP/2020/198952

MS TITLE: QuantifyPolarity, a new tool-kit for measuring planar polarized protein distributions and cell properties in developing tissues

AUTHORS: Su Ee Tan, Weijie Tan, Katherine H Fisher, and David Strutt

ARTICLE TYPE: Techniques and Resources Article

I have looked at your response to the comments from one of the reviewers and in light of your thorough answer I am happy to tell you that your manuscript has been accepted for publication in Development, pending our standard ethics checks.

© 2021. Published by The Company of Biologists under the terms of the Creative Commons Attribution License (https://creativecommons.org/licenses/by/4.0/).
